# Characterisation of the Cullin-3 mutation that causes a severe form of familial hypertension and hyperkalaemia

**DOI:** 10.15252/emmm.201505444

**Published:** 2015-08-18

**Authors:** Frances-Rose Schumacher, Keith Siew, Jinwei Zhang, Clare Johnson, Nicola Wood, Sarah E Cleary, Raya S Al Maskari, James T Ferryman, Iris Hardege, Nichola L Figg, Radoslav Enchev, Axel Knebel, Kevin M O’Shaughnessy, Thimo Kurz

**Affiliations:** 1 MRC Protein Phosphorylation and Ubiquitylation Unit, College of Life Sciences, University of DundeeDundee, UK; 2 Division of Experimental Medicine and Immunotherapeutics, University of CambridgeCambridge, UK; 3 Division of Cardiovascular Medicine, Department of Medicine, University of CambridgeCambridge, UK; 4 Institute of Biochemistry, ETH ZürichZürich, Switzerland

**Keywords:** cullin, CUL3, monogenic hypertension syndromes, proteasome, ubiquitin, WNK/SPAK/OSR1 pathway

## Abstract

Deletion of exon 9 from Cullin-3 (CUL3, residues 403–459: CUL3^Δ403–459^) causes pseudohypoaldosteronism type IIE (PHA2E), a severe form of familial hyperkalaemia and hypertension (FHHt). CUL3 binds the RING protein RBX1 and various substrate adaptors to form Cullin-RING-ubiquitin-ligase complexes. Bound to KLHL3, CUL3-RBX1 ubiquitylates WNK kinases, promoting their ubiquitin-mediated proteasomal degradation. Since WNK kinases activate Na/Cl co-transporters to promote salt retention, CUL3 regulates blood pressure. Mutations in both KLHL3 and WNK kinases cause PHA2 by disrupting Cullin-RING-ligase formation. We report here that the PHA2E mutant, CUL3^Δ403–459^, is severely compromised in its ability to ubiquitylate WNKs, possibly due to altered structural flexibility. Instead, CUL3^Δ403–459^ auto-ubiquitylates and loses interaction with two important Cullin regulators: the COP9-signalosome and CAND1. A novel knock-in mouse model of CUL3^WT^^/Δ403–459^ closely recapitulates the human PHA2E phenotype. These mice also show changes in the arterial pulse waveform, suggesting a vascular contribution to their hypertension not reported in previous FHHt models. These findings may explain the severity of the FHHt phenotype caused by CUL3 mutations compared to those reported in KLHL3 or WNK kinases.

See also: **FC Luft** (October 2015)

## Introduction

Blood pressure is controlled in part by the WNK kinase pathway, which acts to modulate the activity of key cation–chloride co-transporters in the kidney (Kahle *et al*, 2008; Alessi *et al*, 2014). Considerable insight into this pathway has come from exploring the molecular basis for mutations described in patients with a rare monogenic form of hypertension called familial hypertension and hyperkalaemia [FHHt, also known as pseudohypoaldosteronism (PHA2); OMIM: 145260]. Mutations in the genes encoding WNK kinases have been shown to increase protein levels of WNK1 and WNK4 in the kidney, leading to increased activation of this pathway (Wilson *et al*, 2001; Kahle *et al*, 2008). When activated by phosphorylation, the WNK kinases phosphorylate and activate the downstream kinases, SPAK (SPS1-related proline/alanine-rich kinase) and OSR1 (oxidative stress–responsive kinase 1), which in turn phosphorylate and activate cation–chloride co-transporters in the kidney, such as NCC (Na^+^/Cl^−^ co-transporter), which leads to increased sodium ion reabsorption (Vitari *et al*, 2005; Delpire & Gagnon, 2008; Richardson *et al*, 2008, 2011). It is this up-regulation of the WNK signalling pathway that is responsible for the salt retention that drives the hypertension in FHHt. These patients also present with disturbed plasma electrolytes especially hyperkalaemia and a metabolic acidosis, partly driven by NCC activation (Boyden *et al*, 2012; Osawa *et al*, 2013; Tsuji *et al*, 2013; Glover *et al*, 2014; McCormick *et al*, 2014). As FHHt patients are salt-loaded through NCC activation, the hypertension and electrolyte disturbance respond to either salt restriction or low doses of thiazide diuretics (Gordon & Hodsman, 1986; Mayan *et al*, 2002).

More recently, the ubiquitin–proteasome system (UPS) was shown to have a critical upstream role in regulating blood pressure by modulating WNK kinase levels (Mori *et al*, 2013; Ohta *et al*, 2013; Wakabayashi *et al*, 2013; Wu & Peng, 2013). This was first suggested when patients with severe forms of FHHt were described with mutations in Cullin-3 (CUL3; PHA2E), and one of its substrate adaptors, KLHL3 (PHA2D) (Boyden *et al*, 2012; Louis-Dit-Picard *et al*, 2012; OMIM:145260). CUL3 belongs to the Cullin-RING ligase (CRL) family that form the largest class of ubiquitin E3 ligases in the cell. Cullins are elongated proteins, which confer an extended structure to active CRL complexes (Zheng *et al*, 2002; Duda *et al*, 2011). Through their C-terminus, Cullins constitutively bind to a small RING finger protein, either RBX1 or RBX2, which forms the catalytic core of the ligase complex. The binding to various substrate-adaptor proteins at the N-terminal domain enables CUL3-RBX1 to appropriately ubiquitylate a plethora of cellular substrates (Wee *et al*, 2005; Harper & Tan, 2012). In this way, CUL3 acts as an active scaffold to direct ubiquitylation, as C-terminally bound RBX1 binds ubiquitin-conjugating E2 enzymes that are charged with ubiquitin, while simultaneously binding to the substrate adaptor, in this instance KLHL3. The overall structural rigidity of the Cullin, in particular between the elongated N-terminus and the globular C-terminus, is important for the ubiquitin E3 ligase function of the complex. Alterations in the connecting sequence between the Cullin N- and C-terminus are thought to change the relative positioning of the RING domain towards the bound substrate, which can prevent substrate modification (Zheng *et al*, 2002; Liu & Nussinov, 2011). Efficient CRL ubiquitylation also requires the modification of a conserved C-terminal lysine residue (Lys 721 in CUL3) by a single molecule of the ubiquitin-like protein Nedd8 (Pintard *et al*, 2003). This modification, auto-catalysed by CUL3-RBX1, is aided by Nedd8-E3 ligases (Kurz *et al*, 2005; Rabut *et al*, 2011) and alters the flexibility of the C-terminal Cullin-RBX1 module to activate E3 ligase activity (Duda *et al*, 2008; Scott *et al*, 2014). While neddylation is required for efficient ubiquitylation, studies show that the cycling between a neddylated and deneddylated state is crucial in a cellular context (Cope *et al*, 2002; Pintard *et al*, 2003). As such, Cullin interactions with the deneddylation complex COP9-signalosome (CSN), a metalloprotease, are also required for activity. Another regulator of CRLs, CAND1, interacts with the N- and C-terminus of Cullins only in their deneddylated state to promote the exchange of substrate adaptors (Liu *et al*, 2002; Goldenberg *et al*, 2004; Pierce *et al*, 2013; Wu *et al*, 2013; Zemla *et al*, 2013).

In the context of PHA2, KLHL3 is the critical adaptor that links the WNK kinases to CUL3-RBX1, enabling CUL3 to directly ubiquitylate the bound WNK kinase. In earlier work, we and others showed the KLHL3 mutations described in FHHt (PHA2D) patients disrupt the formation of the complex and prevent WNK ubiquitylation, leading to WNK stabilisation and increased flux through the WNK kinase pathway (Mori *et al*, 2013; Ohta *et al*, 2013; Shibata *et al*, 2013; Wakabayashi *et al*, 2013; Wu & Peng, 2013; Schumacher *et al*, 2014; Susa *et al*, 2014). Some patients with FHHt have mutations in WNK kinases, which promote WNK stabilisation by disrupting WNK-KLHL3 interactions (Wakabayashi *et al*, 2013; Schumacher *et al*, 2014) or, in some cases, by the increased expression of WNK mRNA (Wilson *et al*, 2001).

While FHHt patients with CUL3, KLHL3 or WNK mutations present with the same disease, those with mutations in CUL3 display a more severe phenotype, evident in terms of both an earlier age-of-onset and the degree of hypertension and electrolyte disturbance reported (Boyden *et al*, 2012; Osawa *et al*, 2013; Tsuji *et al*, 2013). To date, all the reported CUL3 PHA2E mutations are heterozygous, transmit as autosomal dominant traits and result in the deletion of the amino acids encoded for by exon 9 of the CUL3 mRNA (residues 403–459) (Boyden *et al*, 2012; Grimm *et al*, 2012; Osawa *et al*, 2013; Tsuji *et al*, 2013; Glover *et al*, 2014). Nevertheless, precisely why and how this truncated form of CUL3 (hereafter referred to as CUL3^Δ403–459^) causes a more severe phenotype is currently unclear. We have investigated this question using a biochemical *in vitro* approach to understand the molecular defects caused by the mutation and a mouse model of PHA2E to better understand the physiological basis of the effects of CUL3^Δ403–459^. This has uncovered a number of critical molecular and physiological factors that enhance our understanding of Cullin-RING ligase mechanisms, the physiology of CUL3, and importantly its pathophysiology in FHHt patients.

## Results

### CUL3^Δ403–459^ forms an active Cullin-RING ligase complex

**Figure 1:**
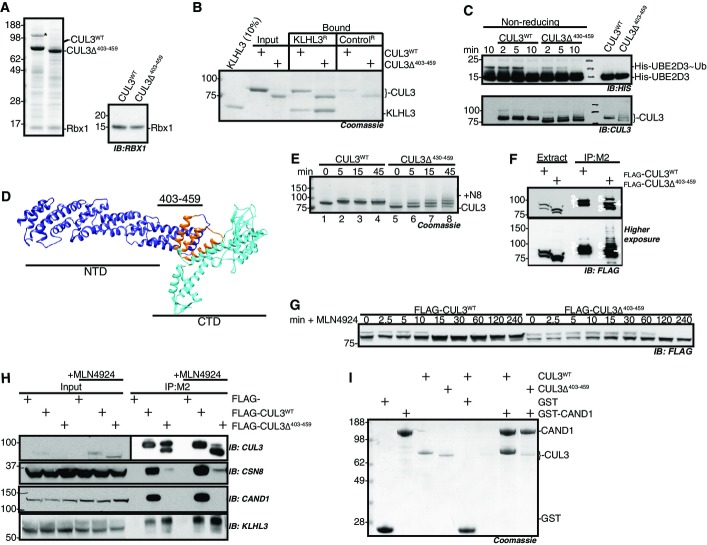
CUL3^Δ403–459^ forms a functional Cullin-RING ligase with altered activity and is unable to interact with CRL regulators
Purified CUL3^WT^ and CUL3^Δ403–459^ analysed by SDS–PAGE and Coomassie blue staining, following expression in SF21 cells, and DAC-tag-affinity purification and tag cleavage (see Materials and Methods). Bands migrating between 62- and 98-kDa protein markers are CUL3^WT^ and CUL3^Δ403–459^, respectively, while the band below 17 kDa is Rbx1. The immunoblot for RBX1 is shown on the right. The band in CUL3^WT^ denoted with * is co-purified insect cell CAND1, which was later removed by SEC. All protein identities were confirmed by mass spectrometry.
KLHL3 binding to CUL3^WT^ and CUL3^Δ403–459^ is comparable as shown by pull-down assays with a KLHL3 resin generated from cross-linking anti-KLHL3 antibody to Protein G sepharose, and then binding saturating amounts of KLHL3 to the anti-KLHL3 resin (denoted KLHL3^R^), the control resin (Control^R^) is Protein G sepharose (see Materials and Methods for further detail). CUL3^WT^ or CUL3^Δ403–459^ was incubated with either KLHL3^R^ or Control^R^ at 4°C for 1 h and then washed thrice with 1× PBS, resin volumes were minimised, and SDS–Laemmli buffer was used to elute protein from resin prior to analysis by SDS–PAGE and staining with colloidal blue.
UBE2D3˜ubiquitin-release assays analysed by non-reducing SDS–PAGE and immunodetection. His-tagged UBE2D3 was charged with ubiquitin by incubation with ATP and UAE, and charging was stopped by ATP depletion with apyrase. No CUL3 (negative control, lane 1), CUL3^WT^ or CUL3^Δ403–459^ was then added to the charged E2 and the activity of the RING domain, RBX1, monitored by the hydrolysis of ubiquitin from UBE2D3, visualised by immunodetection with anti-His antibody (upper panel, “˜” denotes thioester bond). The lower panel is an immunoblot for CUL3, showing equivalent amount of both CUL3^WT^ and CUL3^Δ403–459^ was added to the reaction mix; over time, the appearance of higher molecular weight bands represents the modification of CUL3 by covalent attachment of ubiquitin. The right two lanes are control lanes incubated for 10 min with E2˜Ubi but then treated with reducing agent prior to SDS–PAGE.
Structural model of CUL3^WT^ based on the structure of CUL1 (PDB:1LDK), highlighting the residues deleted in the PHA2E mutant which are located close to the N-terminal domain (NTD) and C-terminal domain (CTD) border, using Phyre2 and Chimera (see Materials and Methods). The NTD is coloured purple, the CTD cyan, and residues 403–459 orange.
Time course of *in vitro* neddylation reactions. Purified NAE, UBE2M and Nedd8 were mixed with either CUL3^WT^ or CUL3^Δ403–459^ and incubated at 30°C for the time indicated. Due to the covalent attachment of Nedd8 (8.6 kDa), modified proteins have decreased mobility. The band shift observed for CUL3^WT^ (lane 1 versus lane 2) is representative of a single Nedd8 modification, while the multiple bands in the CUL3^Δ403–459^ reaction reflect the attachment of up to three Nedd8 molecules (lane 5 versus lane 8).
HEK-293 cells over-expressing either FLAG-CUL3^WT^ or FLAG-CUL3^Δ403–459^ were immunoprecipitated with M2 Flag-binding sepharose, in the presence of a deneddylase inhibitor (OPT). Extract, cells were lysed in the presence of OPT and clarified by centrifugation. IP:M2, clarified supernatant samples were incubated for 1 h at 4°C with M2 (anti-Flag) sepharose, then washed with PBS and eluted with SDS–Laemmli buffer, prior to SDS–PAGE and immunodetection with anti-FLAG antibody. Bands are labelled as follows: 1. CUL3^WT^, 2. CUL3^WT^-N8, 3. CUL3^Δ403–459^, 4. CUL3^Δ403–459^-N8, 5. CUL3^Δ403–459^-2(N8).
Neddylation in HEK-293 cells over-expressing FLAG-CUL3^WT^ or FLAG-CUL3^Δ403–459^ was blocked by treatment with a Nedd8-E1-enzyme inhibitor, 3 μM MLN4924 (Millennium Pharmaceuticals), in cells over-expressing FLAG-CUL3^WT^ and FLAG-CUL3^Δ403–459^ respectively. Cell extracts were sampled over time in the presence of OPT to prevent de-neddylation and subjected to SDS–PAGE and immunodetection of the FLAG tag.
FLAG-CUL3^WT^ or FLAG-CUL3^Δ403–459^ was immunoprecipitated with M2-sepharose as described in (F). +MLN4924 indicates cell culture media supplementation with 3 μM MLN4924 for 5 h prior to cell lysis to achieve complete deneddylation of CUL3. Following SDS–PAGE, immunoblotting with COP9 Signalosome (CSN) or CAND1 antibodies determined the interaction between these Cullin regulators and FLAG-CUL3^WT^ and FLAG-CUL3^Δ403–459^, respectively.
GST-CAND1 pull-down assays with CUL3 proteins. Purified GST or GST-CAND1 immobilised on glutathione–sepharose was mixed with either purified CUL3^WT^ or CUL3^Δ403–459^ and incubated at 4°C for 1 h. The sepharose was then washed thrice and eluted from the resin by incubation with SDS–Laemmli buffer prior to SDS–PAGE and Coomassie staining. CUL3^WT^ but not CUL3^Δ403–459^ interacts with CAND1. Source data are available online for this figure.

To investigate the molecular defect of CUL3^Δ403–459^, we determined whether this form of CUL3 was able to build a CRL complex. First, we established that CUL3^Δ403–459^ binds RBX1 similar to CUL3^WT^, by showing that both versions of CUL3 form a stable complex with RBX1 when co-expressed in insect cells (Fig 1A). A second critical interaction for a functional CRL is the ability to bind substrate-adaptor proteins, and in the context of WNK kinase modification, CUL3 must interact with KLHL3 (Lamark & Johansen, 2012; Ji & Privé, 2013; Canning & Bullock, 2014). The 57-residue deletion in CUL3^Δ403–459^ lies in a structurally distinct domain of CUL3 and would not be expected to perturb binding to substrate adaptors. We confirmed that CUL3^Δ403–459^ indeed retains the ability to bind KLHL3 by *in vitro* pull-down assays with purified proteins (Fig 1B). We also showed this interaction is maintained in a cellular system, using ectopically expressed FLAG-tagged CUL3 to co-precipitate endogenous KLHL3 in FLAG immunoprecipitates (Fig 1H, lower panel). Thus, CUL3^Δ403–459^ maintains crucial interactions important for ubiquitin-ligase function. Given this, we tested whether CUL3^Δ403–459^-RBX1 also maintains the ability to hydrolyse the thioester bond between the catalytic cysteine residue of a recruited E2 enzyme and ubiquitin (E2∼UB), a critical step in ubiquitylation and a measure for catalytic activity. Using ubiquitin-release assays from charged E2 enzymes, we show that CUL3^Δ403–459^ hydrolyses E2∼UB more efficiently than CUL3^WT^, demonstrating a functionally intact CUL3^Δ403–459^-RBX1 catalytic core, while also suggesting potential hyper-activity (Fig 1C). Together, these results demonstrate that CUL3^Δ403–459^ maintains interactions and basic functionality critical for CRL activity.

### Structural modelling suggests CUL3^Δ403–459^ is more flexible than CUL3^WT^

To better understand how the deletion in CUL3^Δ403–459^ may affect the CRL, we predicted the structure of CUL3, using closely related Cullin-1 (CUL1; Fig 1D; Appendix Fig S1). The region deleted in CUL3^Δ403–459^ likely forms a three-helical bundle juxtaposed to the globular C-terminal domain. Removal of these helices would fuse together two short unstructured regions to create a longer loop, which, as well as shortening the elongated structure of the CRL complex, may increase the flexibility between the two domains. If correct, this model explains how RBX1 and KLHL3 binding is maintained (Munir *et al*, 2007; Calabrese *et al*, 2011), while also suggesting the function of the ligase may be compromised due to the altered relative orientation of the CRL components towards each other. Studies investigating the structure of CUL1 demonstrated the importance of a rigid Cullin conformation for substrate ubiquitylation, as increasing the flexibility of CUL1, in an area that corresponds to the CUL3 deletion, led to strongly decreased substrate ubiquitylation (Zheng *et al*, 2002; Appendix Fig S1). It is therefore possible that due to the presence of a predicted unstructured flexible loop, CUL3^Δ403–459^ may similarly sample non-productive conformations.

### CUL3^Δ403–459^ has altered Nedd8-ligase activity and is unable to interact with the CRL regulators COP9-signalosome and CAND1

**Figure EV1:**
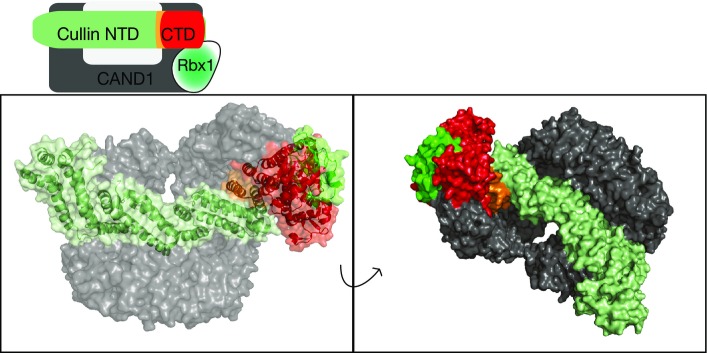
Structural context of the area deleted by the disease-causing mutation in CUL3 Top: Schematic representation of the interactions between CRL and CAND1. Bottom: Representation of CAND1 in complex with CUL1-RBX1, PDB 1u6g (Goldenberg *et al*, 2004). Coloured as shown in the schematic representation. Residues 437–493 of CUL1 (equivalent to 403–456 CUL3) are coloured orange.

Available structural data of CRL proteins bound to Cullin regulators suggest the deleted region in CUL3^Δ403–459^ may also influence neddylation and binding of the CSN (Lingaraju *et al*, 2014) and of CAND1 (Goldenberg *et al*, 2004; Fig EV1). This prompted us to examine whether these interactions are affected by the PHA2E mutation.

To investigate the ability of CUL3^Δ403–459^ to be auto-modified with Nedd8, we used *in vitro* neddylation assays with purified CUL3, Nedd8 E1 (APPBP1-UBA3), Nedd8 E2 (UBE2M) and Nedd8. As Nedd8 forms a covalent isopeptide bond with CUL3, neddylation can be visualised as a slower-migrating band on SDS–PAGE. We found CUL3^Δ403–459^ retains the ability to auto-neddylate (Fig 1E, Appendix Fig S2A). However, CUL3^Δ403–459^ appears less efficient at transferring Nedd8, as unlike for CUL3^WT^, some unmodified CUL3^Δ403–459^ remained even 45 min after neddylation was initiated. Moreover, while CUL3^WT^ was only mono-neddylated, multiple Nedd8 molecules were attached to CUL3^Δ403–459^ (Fig 1E). Both of these differences in neddylation are consistent with increased structural flexibility of CUL3^Δ403–459^.

As reported (McCormick *et al*, 2014), we also noticed that CUL3^Δ403–459^ neddylation is elevated in cells (Fig 1F). CRLs cycle between neddylated and de-neddylated states to maintain activity (Pintard *et al*, 2003). Therefore, the increased cellular neddylation of CUL3^Δ403–459^ could reflect a decrease in de-neddylation by the isopeptidase CSN. The structure of the CSN bound to CUL1 supports this idea, as the Cullin C-terminal domain, including the region equivalent to residue 403–459 in CUL3, contacts the CSN subunits directly (Lingaraju *et al*, 2014). To investigate this possibility, we blocked neddylation using an inhibitor of the Nedd8-E1 enzyme (MLN4924; Millennium Pharmaceuticals) and monitored CUL3 de-neddylation over time. Consistent with decreased rates of deneddylation, CUL3^Δ403–459^ remained neddylated for longer than CUL3^WT^ (Fig 1G) and also showed markedly reduced binding to CSN subunits (Fig 1H, Appendix Fig S2B). As such, the observed elevated Nedd8 modification of CUL3^Δ403–459^ in cells is likely due to a combination of Nedd8 ligation to non-physiological lysine residues and of reduced CSN de-neddylation.

**Figure 2:**
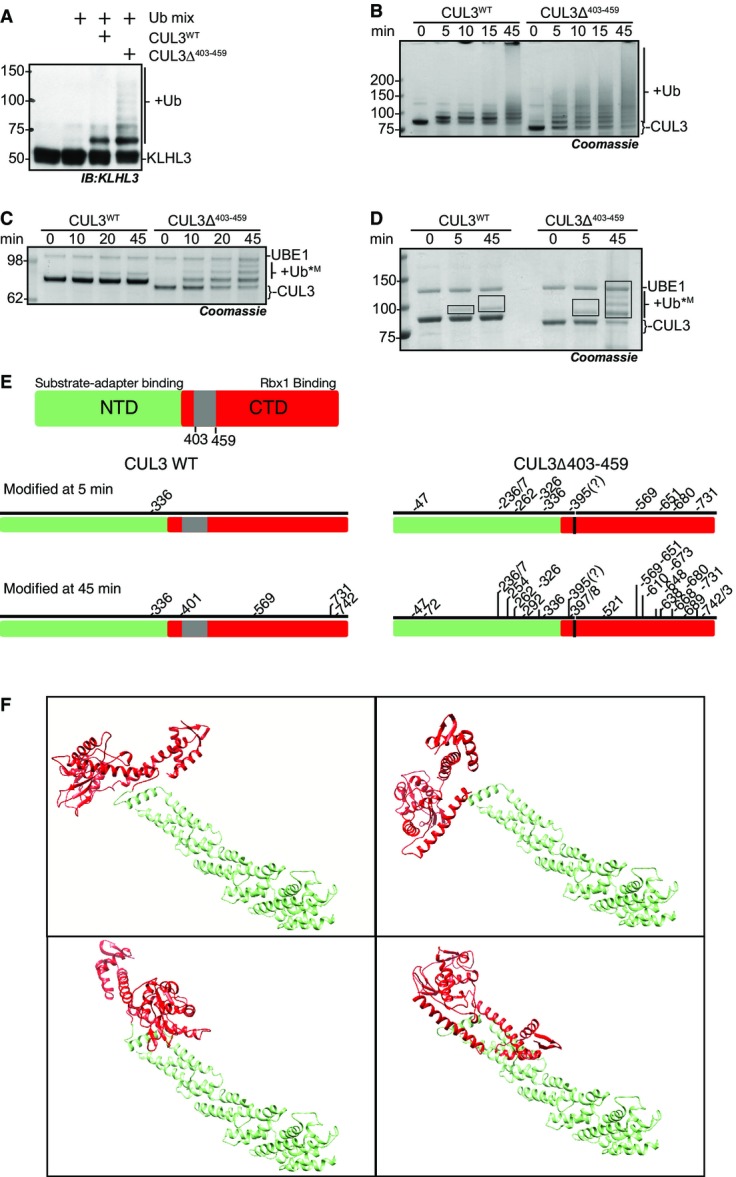
CUL3^Δ403–459^ displays increased auto-ubiquitylation and ubiquitylation of KLHL3 A–D *In vitro* ubiquitylation assays with purified proteins. As ubiquitin is covalently attached to the substrate lysine residue, the appearance of higher molecular weight protein bands reflects the modification of the protein with ubiquitin. All assays contain purified UBE1, UBE2D3, ubiquitin, 0.1 mM ATP, 1 mM MgCl_2_ and were buffered in 50 mM HEPES, 150 mM NaCl and incubated at 30°C for the time indicated_._ Reactions were stopped by the addition of SDS–Laemmli buffer to a concentration of 1×. SDS–PAGE, staining with Coomassie blue, or detection with the indicated antibody following immunoblotting enabled the visualisation of ubiquitylation. (A) to determine the relative modification of KLHL3 by CUL3^WT^ and CUL3^Δ403–459^, KLHL3 was included into the reaction at 2× molar concentration over CUL3^WT^ and CUL3^Δ403–459^. (B, C) Reactions serve to determine basal auto-ubiquitylation of CUL3^WT^ or CUL3^Δ403–459^ and do not contain KLHL3 or other potential substrate proteins. Lysine residues on CUL3^WT^ or CUL3^Δ403–459^ act as the substrate. (B) High molecular weight bands reflect ubiquitin chain linkages on CUL3 or the multiple mono-ubiquitylation of a number of CUL3 lysine residues. (C) Activity assays contain methylated ubiquitin, a form of ubiquitin incapable of forming ubiquitin chains; as such, higher band shifts reflect the attachment of mono-ubiquitin to one more lysine residue on CUL3^WT^ or CUL3^Δ403–459^, respectively. (D) Activity assay performed as in (C) with methyl-ubiquitin, the boxes shown on the gel are indicative of the gel pieces excised for mass spectrometry analysis in (F).
E Schematic representation (to linear residue scale) highlighting the domains of CUL3 and schematic representation of the lysine residues of CUL3^WT^ or CUL3^Δ403–459^, modified at 5 and 45 min, respectively, in the *in vitro* ubiquitylation assay shown in (D) and as determined by mass spectrometry.
F Structural docking model of CUL3^Δ403–459^ based on the structure of full-length CUL1 (1LDK) using Chimera (see Materials and Methods). The NTD is coloured green, while the CTD is red, and the four panels sample possible positioning of the CTD relative to the NTD in CUL3^Δ403–459^, assuming full flexibility of the linker after deletion of three helices encoded by exon 9. Source data are available online for this figure.

CRLs must be de-neddylated for a productive interaction with the substrate-adaptor exchange factor CAND1. Furthermore, the structure of CUL1 bound to CAND1 suggests that, regardless of neddylation state, CUL3^Δ403–459^ may be unable to form the required critical interactions (Goldenberg *et al*, 2004; Pierce *et al*, 2013; Wu *et al*, 2013; Zemla *et al*, 2013). We therefore investigated whether CUL3^Δ403–459^ was indeed defective in binding to CAND1. We treated cells expressing either FLAG-CUL3^WT^ or FLAG- CUL3^Δ403–459^ with the neddylation inhibitor MLN4924 to force all of CUL3 into the non-neddylated state and then determined interaction with CAND1 by immunoprecipitation. While CUL3^WT^ strongly bound to CAND1 in these assays, CUL3^Δ403–459^ was unable to interact with CAND1 (Fig 2H, Appendix Fig S2B). We confirmed these findings using *in vitro* assays with purified proteins (Fig 1I). These data advocate that structural perturbations prevent CUL3^Δ403–459^ from binding to CAND1. In combination with the impairment of CSN binding, such defects suggest that the regulation of CUL3^Δ403–459^ in cells is likely severely disrupted.

### CUL3^Δ403–459^ displays enhanced auto-ubiquitylation

Our data indicate that the regulation of CUL3^Δ403–459^ is defective in cells, and we wanted to test whether its ubiquitylation activity is similarly affected. McCormick *et al* suggested that CUL3^Δ403–459^ may stabilise WNK kinases by ectopically degrading the substrate adaptor KLHL3, preventing the formation of functional CUL3-KLHL3 complexes (McCormick *et al*, 2014). Some CRLs are known to ubiquitylate bound substrate adaptors (Wee *et al*, 2005), and it is feasible that altering the CUL3 backbone structure could indeed lead to erroneous ubiquitylation of KLHL3. In our *in vitro* system, we confirmed that CUL3^Δ403–459^ directly modified KLHL3 with a greater efficiency than CUL3^WT^ (Fig 2A, Appendix Fig S2C). Interestingly, we observed that CUL3^Δ403–459^ also exhibited markedly increased auto-ubiquitylation (Fig 2B, Appendix Fig S2D).

**Figure EV2:**
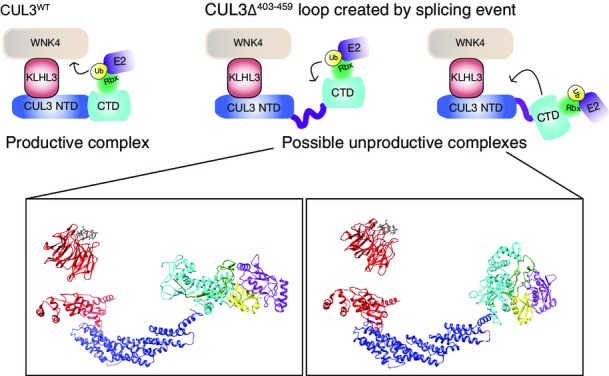
Schematic representation of the possible structural effects of the mutation The top schematics represent a functional CUL3^WT^ complex and show two contrasting positionings for an active CUL3 ^Δ403–456^ that is unable to modify the WNK kinases. The lower structural representations correspond to the schematics above. These were generated in Chimera, and known structures were utilised as docking references to enable the possible orientations to be explored. KLHL3-KLECH domain (red) bound to a WNK peptide (grey) PDB: 4CH9. KLHL3-BTB domain (red) bound to CUL3-N-terminal domain (blue), CUL3-C-terminal domain (cyan) (based on CUL1 CTD) PDB: 1LDK. RBX1 (green), UBE2D (purple) and ubiquitin (yellow) were docked based on the complex RNF4-E2-UB structure PBD: 4AP4.

Auto-ubiquitylation is often utilised as a read-out for ubiquitin-ligase activity. In a cellular context, self-modification can lead to auto-degradation of the ligase (Silke *et al*, 2005). If CUL3^Δ403–459^ modifies lysine residues inaccessible to CUL3^WT^, this could explain the increased auto-ubiquitylation. Alternatively, CUL3^Δ403–459^ could build longer ubiquitin chains on the same lysine residues that are also targeted by CUL3^WT^. To explore these possibilities, we performed *in vitro* ubiquitylation assays with methylated ubiquitin, a form of ubiquitin unable to build ubiquitin chains (Kirisako *et al*, 2006). In these assays, CUL3^Δ403–459^ was auto-modified on multiple lysine residues, while CUL3^WT^ only mono-ubiquitylated one residue (Fig 2C). Therefore, the observed increase in auto-ubiquitylation is most likely due to erroneous modification of multiple lysine residues on CUL3^Δ403–459^, which is consistent with an increase in structural flexibility as suggested by our model. Alternatively, the mutation may change the active site environment of CUL3, exposing more lysine residues close to the E2∼UB binding site. To distinguish between these possibilities, we mapped the lysine residues modified in our *in vitro* assay system using mass spectrometry. We performed *in vitro* auto-ubiquitylation reactions of CUL3 with methylated ubiquitin for either 5 or 45 min and determined the ubiquitylated lysine residues by analysing which lysine residue(s) carry di-glycine remnants after trypsin digest (Fig 2D and E; Kim *et al*, 2011). Using this approach, we found that after 5 min, CUL3^WT^ was only auto-ubiquitylated on a single lysine residue (K336), while ten lysine residues on CUL3^Δ403–459^ were modified (Fig 2D and E). After running the reaction for 45 min, the modification sites on CUL3^WT^ had increased to five, all of which were C-terminal to the original site and contained within the globular C-terminal Cullin domain. The ubiquitylation sites on CUL3^Δ403–459^, however, had increased to 25 sites distributed along the entire length of the protein (most N-terminal: K47; most C-terminal: K743). These results support our model that the CUL3 deletion induces increased structural flexibility, leading to the erroneous auto-ubiquitylation of sites normally not accessible to CUL3^WT^ (Figs 2E and F and EV2).

### CUL3^Δ403–459^ cannot ubiquitylate WNK kinases

**Figure 3:**
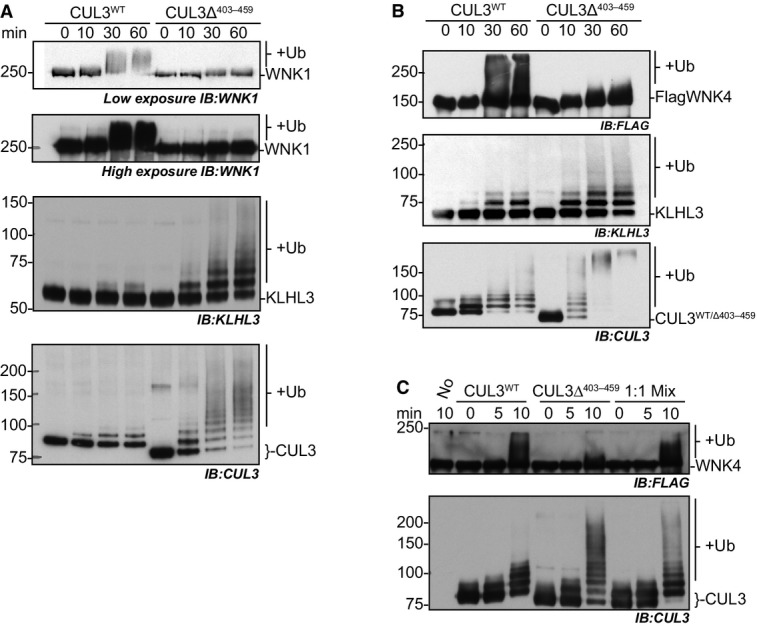
CUL3^Δ403–459^ is unable to ubiquitylate WNK1 or WNK4 kinases in an *in vitro* system, and this deficiency cannot be rescued by the presence of CUL3^WT^ A–C *In vitro* ubiquitylation assays were performed as described in Figure 2, but with the addition of immunoprecipitated WNK1 in (A), or immunoprecipitated over-expressed FLAG-WNK4 in (B, C) (see Materials and Methods). The WNK kinases are modified by CUL3^WT^-KLHL3, with the higher molecular weight smear observed in anti-WNK1 and anti-FLAG panels representative of multiple ubiquitin molecules being covalently attached to the WNK protein. CUL3^Δ403–459^ is unable to modify WNKs. Samples from the same assay reactions were divided to allow immunodetection of the different protein components modified within the same assay reaction. (C) CUL3^WT^, CUL3^Δ403–459^ and an equimolar solution CUL3^WT^:CUL3^Δ403–459^ (1:1 Mix) were incubated with KLHL3 and immunoprecipitated FLAG-WNK4 in ubiquitylation reactions to determine the influence of CUL3^Δ403–459^ on the ubiquitylation activity of CUL3^WT^. Notably, the presence of CUL3^Δ403–459^ does not inhibit WNK ubiquitylation by CUL3^WT^.

As CUL3^Δ403–459^ is able to more efficiently auto-ubiquitylate itself and KLHL3, we next asked whether it similarly shows increased ubiquitylation towards a bound WNK substrate in *in vitro* ubiquitylation assays using purified components. As we previously showed, CUL3^WT^ efficiently ubiquitylates WNK kinases *in vitro* (Fig 3; Ohta *et al*, 2013). CUL3^Δ403–459^, however, while maintaining the ability to efficiently modify itself and KLHL3, was not able to modify either WNK1 or WNK4 (Fig 3A and B). As described for CUL1 (Zheng *et al*, 2002), increasing the flexibility of the Cullin backbone can lead to a decrease in substrate modification, while promoting excessive modification of other CRL components (Zheng *et al*, 2002). Thus, increased structural flexibility of CUL3^Δ403–459^ may lead to similar defects. If so, then it becomes important to understand how this *in vitro* finding can explain the increased WNK stabilisation found *in vivo*. It is possible that only one of the three defects, the lack of substrate ubiquitylation, the increased Cullin auto-ubiquitylation or the increased KLHL3 ubiquitylation, is the major driver for the observed phenotypes. Decisively, KLHL3 levels are not decreased in the kidney of CUL3^WT/Δ403–459^ mice, while the overall CUL3 levels are lower. This suggests that KLHL3 degradation does not contribute to the observed PHA2E phenotype, and instead, we propose that CUL3^Δ403–459^ stabilises WNK levels in two ways: firstly, CUL3^Δ403–459^ excessively auto-ubiquitylates, leading to its own degradation; and secondly, any CUL3^Δ403–459^ protein that escapes degradation cannot promote the ubiquitylation of the WNK kinases. Importantly, all PHA2E patients are heterozygous for the mutation, suggesting that with respect to blood pressure regulation either CUL3^Δ403–459^ behaves as a dominant-negative or CUL3^WT^ is haploinsufficient. Our data demonstrate that the mutation causes a loss-of-function, so a dominant-negative effect seems less likely. We also find that *in vitro*, equimolar amounts of CUL3^Δ403–459^ do not inhibit CUL3^WT^ (Fig 3C), further supporting the concept of haploinsufficiency.

### CUL3^WT/Δ403-459^ mice have increased flux through the WNK kinase pathway and typical FHHt phenotypic features of electrolyte imbalance

**Figure 4:**
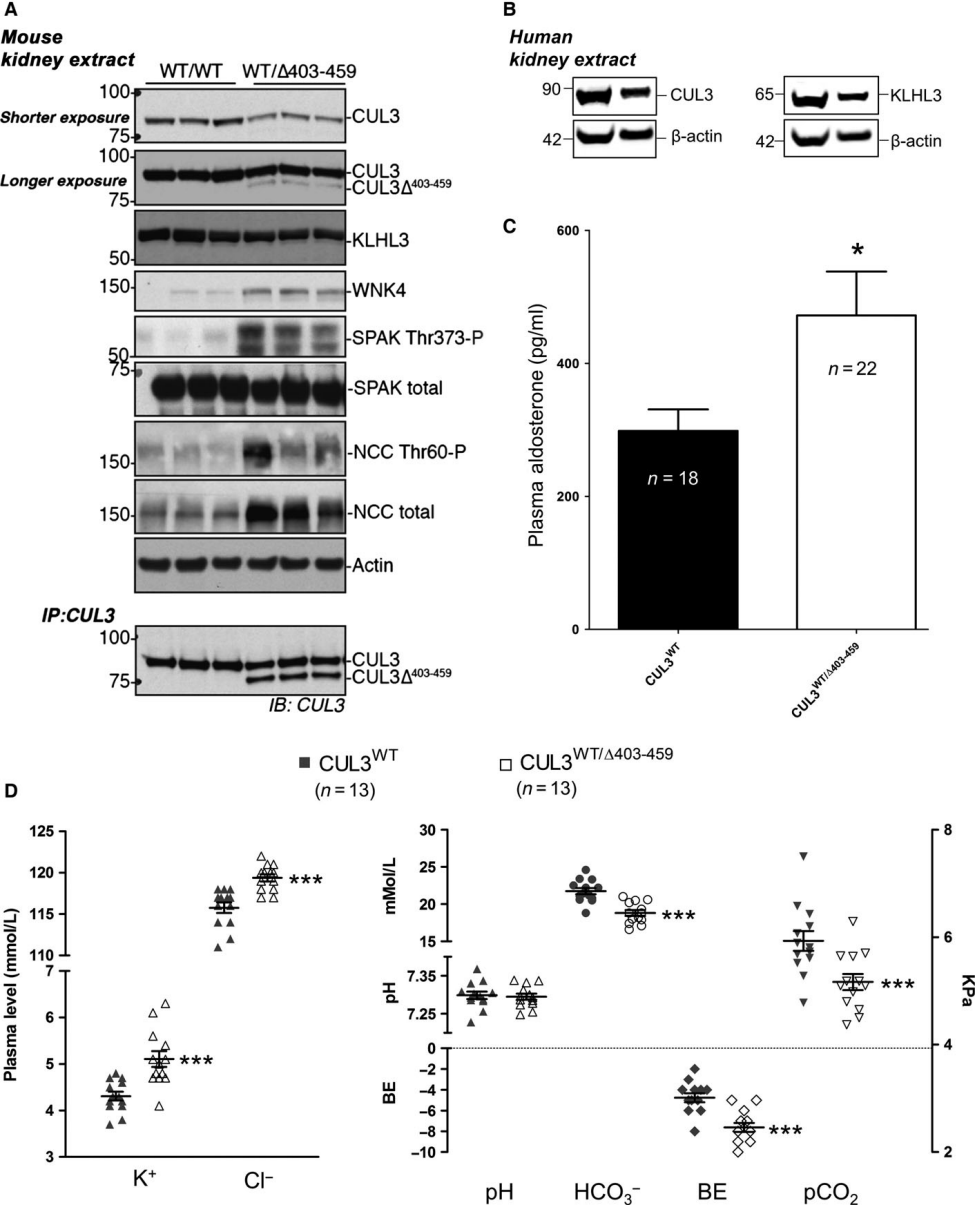
CUL3^WT^^/Δ403–459^ mice recapitulate PHAII electrolyte imbalances due to over-activation of the renal WNK4-SPAK pathway Western blot of whole kidney lysates from mice culled after a minimum 4-h fast. Following exsanguination after surgery, mouse tissues were rapidly harvested and stored, samples were then homogenised, clarified and quantified prior to SDS–PAGE. Immunodetection with the antibodies shown highlights elevated signalling through the WNK kinase pathway in CUL3^WT^^/Δ403–459^ versus CUL3^WT^ mice. The lowest panel is an anti-CUL3 immunoprecipitation of the kidney lysate samples, whereby the CUL3 antibody was cross-linked to Protein G sepharose and used to affinity purify CUL3^WT^ and CUL3^Δ403–459^ from kidney lysates. The samples were incubated together overnight to allow deneddylation of CUL3 proteins to occur and maximise CUL3 binding to the anti-CUL3 resin. Samples were thoroughly washed and eluted from the resin prior to SDS–PAGE and immunodetection for CUL3. The IP highlights that CUL3^Δ403–459^ is indeed present within the kidney lysate.
Fresh frozen kidney tissues from transplant patient and donor cadavers were homogenised, clarified and quantified prior to SDS–PAGE. Western blot analysis confirmed the expression of KLHL3 and CUL3 in normal healthy human kidneys.
Plasma aldosterone after a minimum 4-h fast was calculated by HTRF (homogeneous time-resolved fluorescence) aldosterone assay. The average aldosterone level per mouse was calculated from duplicate samples run in parallel on the assay. Blood was rapidly harvested in heparin-coated plasma extraction tubes following exsanguination after surgery, and samples were snap-frozen for storage. A 58% increase in aldosterone was detected in CUL3^WT^^/Δ403–459^ versus CUL3^WT^ mice (*P = 0.0245). Two-tailed unpaired Student’s *t*-test; data are mean ± SEM.
Arterial blood biochemistries after a minimum 4-h fast. Under anaesthesia, the right carotid artery was cannulated to minimise atmospheric exposure of samples collected for iSTAT blood gas and electrolyte measurements. CUL3^WT^^/Δ403–459^ mice present with abnormal electrolyte homoeostasis compared to CUL3^WT^ mice, exhibiting hyperkalaemia (***P = 0.0004) and hyperchloraemia (***P = 9.5 × 10^−5^) with a compensated metabolic acidosis (*P* = 0.7766), marked by a decrease in bicarbonate (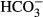) (***P = 3.4 × 10^−5^), base excess (BE) (***P = 9.1 × 10^−5^) and partial pressure of carbon dioxide (pCO_2_) (***P = 0.0038). Two-tailed unpaired Student’s *t*-test; data are mean ± SEM. Source data are available online for this figure.

To complement our *in vitro* findings and to determine more accurately what occurs in PHA2E patients, we engineered a knock-in (KI) mouse carrying the same exon 9 deletion of CUL3 as reported in PHA2E pedigrees (CUL3^Δ403–459^; Appendix Fig S3). Despite setting up heterozygous mating pairs, no homozygous KI mice were born suggesting that, like deletion of the entire coding region of CUL3, the homozygous deletion of residues 403–459 is lethal (Singer *et al*, 1999). Nevertheless, all the reported PHA2E pedigrees are heterozygous, so we phenotyped the heterozygous KI mice. Utilising both immunoblotting of whole kidney lysates and immunofluorescence confocal microscopy of kidney sections, we determined the relative abundance and distribution of proteins involved in the WNK signalling pathway. Whole kidney lysate was analysed by immunoblotting confirming the expression of CUL3 and the presence of an additional band corresponding to the expected PHA2E form, CUL3^Δ403–459^, in the heterozygous mice (Fig 4A). Increased levels of WNK4 and the downstream components of the WNK kinase pathway, reported in other mouse models of FHHt, were also observed (Fig 4A). Specifically, there were striking increases in both total and phosphorylated forms of SPAK and NCC (Fig 4A). We also confirmed the expression of KLHL3 and CUL3 in normal human kidney lysates (Fig 4B). In the context of our *in vitro* data, we were especially interested in examining the levels of KLHL3 and CUL3 in the mouse kidney (Fig 4A). Definitively, the overall level of KLHL3 was comparable between CUL3^WT/WT^ and CUL3^WT/Δ403–459^ mice. In contrast, CUL3^Δ403–459^ appears to be less abundant than CUL3^WT^ in the heterozygous mice. These observations suggest that CUL3^Δ403–459^ does not promote KLHL3 degradation *in vivo* and, moreover, that the lower levels of CUL3^Δ403–459^ may be due to the propensity of CUL3^Δ403–459^ to self-modify presumably causing degradation through the UPS. The lower level of observable CUL3^Δ403–459^ may also be due to non-proteolytic hyper-modification of CUL3^Δ403–459^ with either Nedd8 or ubiquitin. Indeed, this may in part be the case, as incubation of kidney extract at room temperature to promote de-conjugation of Nedd8 or ubiquitin increased the abundance of the faster migrating CUL3^Δ403–459^ band, but did not restore it to CUL3^WT^ levels (Fig 4A, lower panel). Regardless of precisely why, the stabilisation of WNK4 and increased signalling through the WNK cascade is consistent with a PHA2E model.

**Figure EV3:**
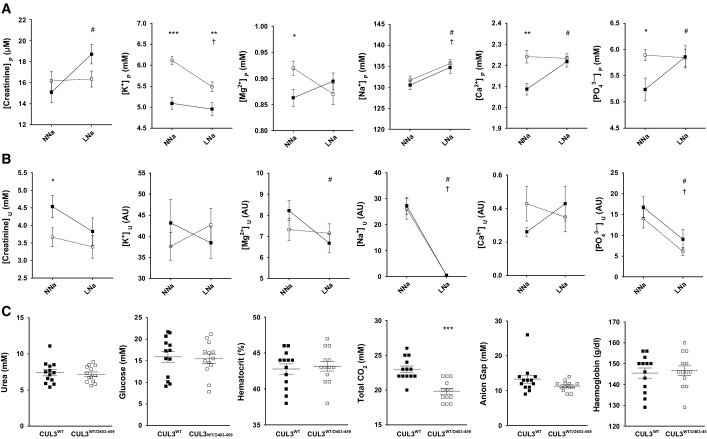
Plasma and urine electrolyte response to normal and low sodium diets A–C The upper (A) and middle panels (B) show plasma (P) and urinary (U) electrolytes, respectively, for CUL3^WT^ mice (▀) versus CUL3^WT^^/Δ403–459^ (□) on either a normal-salt (NNa) (0.3%) or low-salt (LNa) (0.03%) diet measured by ICP-OES analysis. The urinary values (AU) are individually ratioed to the urinary concentration of creatinine ([analyte]_U_/[creatine]_U_). The lower panel (C) shows blood biochemistries not reported in Figure D taken after a minimum 4-h fast by iSTAT analysis. The differences between genotypes that are significantly different are shown as *, the differences between NNa and LNa for CUL3^WT^ that are significantly different are shown as #, and the differences between NNa and LNa for CUL3^WT^^/Δ403–459^ that are significantly different are shown as † (*n*-values as follows: CUL3^WT^: plasma NNa = 16; plasma LNa = 17; urine NNa = 18; urine LNa = 16; blood biochemistry = 13; CUL3^WT^^/Δ403–459^: plasma NNa = 23; plasma LNa = 23; urine NNa = 21; urine LNa = 19; blood biochemistry = 13). Two-tailed unpaired Student’s *t*-test for comparisons between genotypes and two-tailed paired Student’s *t*-test for comparisons between diets within the same genotype; data are mean ± SEM. A full table of *P*-values for this figure is shown in Appendix Table S1. Source data are available online for this figure.

Over-activation of the WNK pathway would be expected to perturb electrolyte homoeostasis. We confirmed this by measuring urine electrolytes, blood biochemistries and aldosterone levels in the mice (Figs 4C and D and EV3). The CUL3^WT/Δ403–459^ mice showed the typical electrolyte disturbance of FHHt with hyperkalaemia, hyperchloraemia and a compensated metabolic acidosis, with the pH maintained by an increased respiratory drive to increase CO_2_ removal and hence reduced total CO_2_ and pCO_2_ levels. The mice also had elevated aldosterone levels (Fig 4C) driven by the hyperkalaemia without pronounced hypervolaemia as evidenced by similar haematocrit levels (Fig EV3). Hypermagnesaemia and hyperphosphataemia were noted on detailed electrolyte analysis of plasma and urine, which are the converse of the abnormalities reported in Gitelman syndrome (Fig EV3; Akhtar & Hafeez, 2009; Rafiqi *et al*, 2010; Pathare *et al*, 2012; Ali *et al*, 2013; Zhang *et al*, 2015). Additionally, we also found that the CUL3^WT/Δ403–459^ mice have significantly lower body weight and body length relative to the WT mice (Appendix Fig S4). This is consistent with Gordon’s original observation that children with PHA2 may present with a low percentile body weight and height for their age (Gordon, 1986) and more recent reports that CUL3^WT/Δ403–459^ patients are growth retarded (Osawa *et al*, 2013; Tsuji *et al*, 2013).

### WNK4 and SPAK accumulate and form puncta in the distal convoluted tubule of CUL3^WT/Δ403–459^ mice

**Figure EV4:**
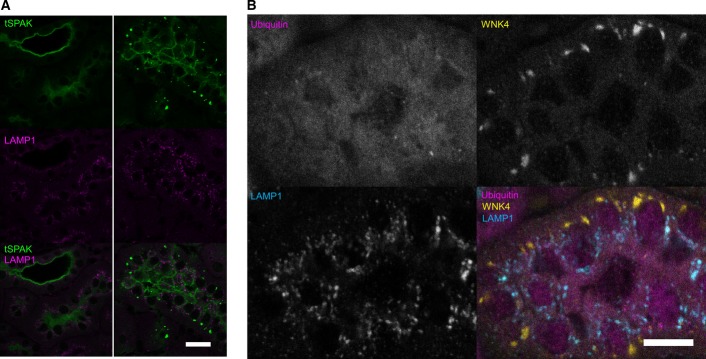
WNK4 and SPAK puncta do not colocalise with LAMP1 or form ubiquitin-containing aggregates Representative pseudocoloured confocal single focal plane images of immunofluorescently stained kidney sections (*n *= 4 per genotype). WNK4 and SPAK form discrete puncta in the distal convoluted tubule of CUL3^WT^^/Δ403–459^ mice. These puncta do not colocalise with lysosomes (LAMP1) or form ubiquitylated aggregates. The puncta have a predominantly basolateral preference with several large juxta-nuclear puncta per cell. Immunolocalisation of total SPAK protein (tSPAK) puncta and LAMP1 in the distal convoluted tubule of CUL3^WT^^/Δ403–459^ versus CUL3^WT^ mice at a 4-h fasting baseline. Scale bar, 20 μm.
Immunolocalisation of total WNK4 puncta, ubiquitin and LAMP1 in the distal convoluted tubule of CUL3^WT^^/Δ403–459^ versus CUL3^WT^ mice at a 4-h fasting baseline. Scale bar, 10 μm.

**Figure 5:**
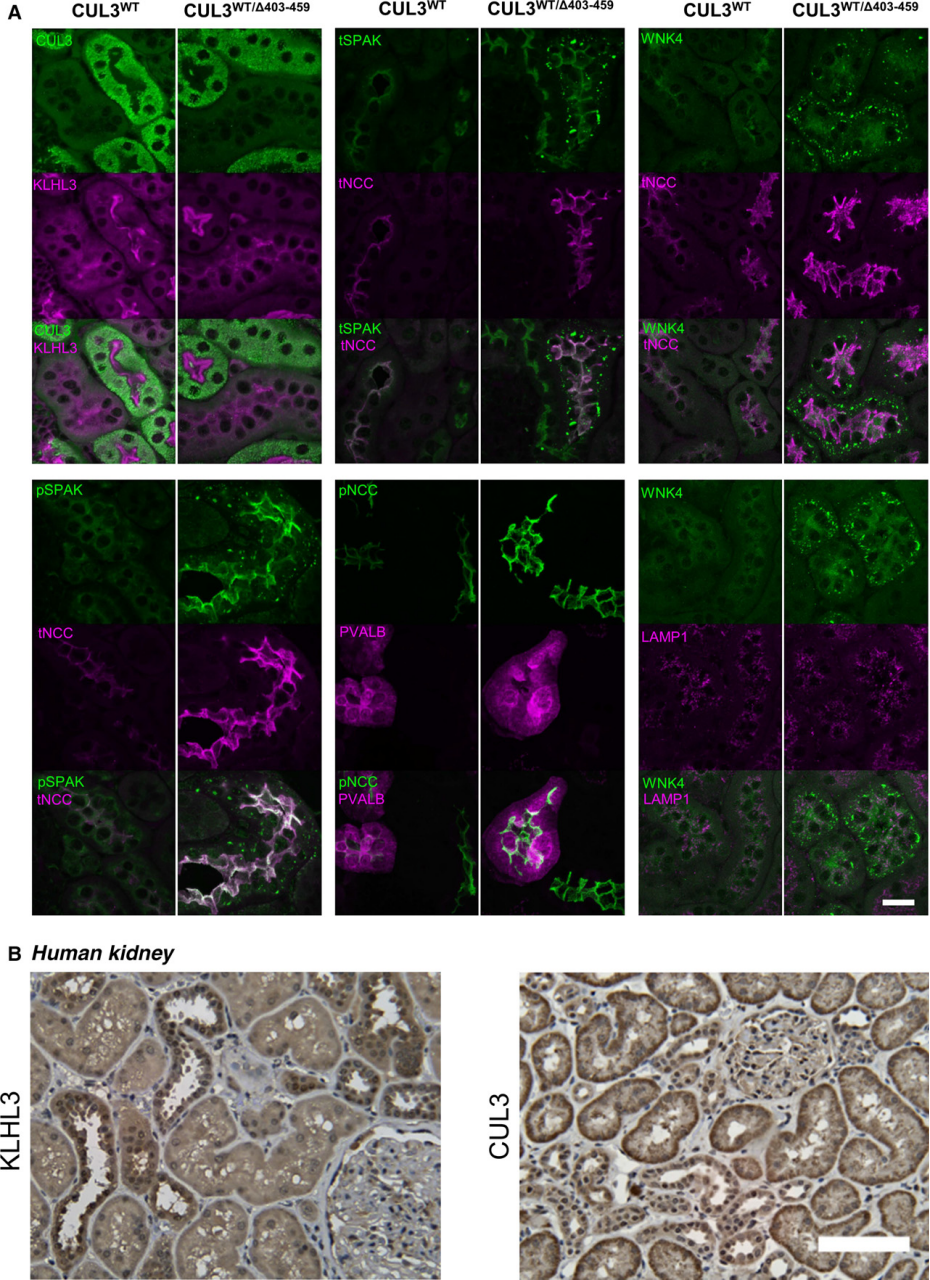
WNK4 and SPAK specifically accumulate and form puncta in the distal convoluted tubule of CUL3^WT^^/Δ403–459^ mice Representative pseudocoloured maximum-intensity *z* projections of immunofluorescently stained kidney sections showing the distribution of the WNK/SPAK pathway components between CUL3^WT^^/Δ403–459^ and CUL3^WT^ mice at a minimum 4-h fasting baseline (*n *= 4 per genotype). CUL3 and KLHL3 are comparable between genotypes, with significantly higher levels of CUL3 in the proximal convoluted tubule (PCT) compared to the weak staining in distal convoluted tubule (DCT) and thick ascending limb of the loop of Henle (TAL), while KLHL3 shows higher expression in the DCT/TAL cytosol with staining of the PCT confined to the apical membrane. Total (t) and phospho (p) NCC Thr44 and SPAK Thr243 show increased apical membrane expression in the parvalbumin (PVALB)-positive early and late (PVALB-negative) DCT of CUL3^WT^^/Δ403–459^ mice. Unexpectedly, the increased levels of WNK4 and SPAK resulted in the formation of discrete puncta specifically in the DCT of CUL3^WT^^/Δ403–459^ mice. It is possible that the autophagy–lysosomal system may attempt to compensate and degrade these excess proteins although the WNK4 puncta do not colocalise with the lysosomal marker LAMP1. Scale bar, 20 μm.
Representative immunohistochemical staining of KLHL3 and CUL3 in human kidney sections (*n *= 6). KLHL3 shows preferential DCT/TAL cytosolic staining similar to the mouse despite no evidence of PCT apical staining as seen in (A). CUL3 exhibits preferential basolateral cytosolic staining of the PCT with similarly low levels of diffuse staining in the DCT/TAL to that of the mouse. Scale bar, 100 μm.

Using immunofluorescence confocal microscopy of kidney sections, we studied the distribution of proteins involved in the WNK kinase cascade in both CUL3^WT/WT^ and CUL3^WT/Δ403–459^ mice (Fig 5A). CUL3^WT/Δ403–459^ mice showed a similar CUL3 distribution compared to both the WT mice, and to the WT human kidney (Fig 5A and B). This was in keeping with the abundance observed in the kidney immunoblots (Fig 4A). In contrast, staining for WNK4 and SPAK in the CUL3^WT/Δ403–459^ mice revealed these proteins to have a striking punctate appearance in the DCT, but not in adjacent segments of the nephron including the thick ascending limb (TAL; Figs 5A and EV4). While WNK1 and OSR1 puncta have been reported in the DCT of SPAK knockout mice, these were smaller in size and far less numerous (Grimm *et al*, 2012). As these puncta do not co-localise with the lysosomal marker LAMP1 or form ubiquitin-containing aggregates (Figs A and EV4), they may represent an accumulation of WNK and SPAK proteins within a protein-processing compartment, such as the trans-Golgi network or activation of secondary protein degradation pathways or storage compartments, due to failed proteasomal clearance (D’Urso *et al*, 1998; Lamark & Johansen, 2012; Wolff *et al*, 2014).

### CUL3^WT/Δ403–459^ mice have hypertension and a novel vascular phenotype

**Figure 6:**
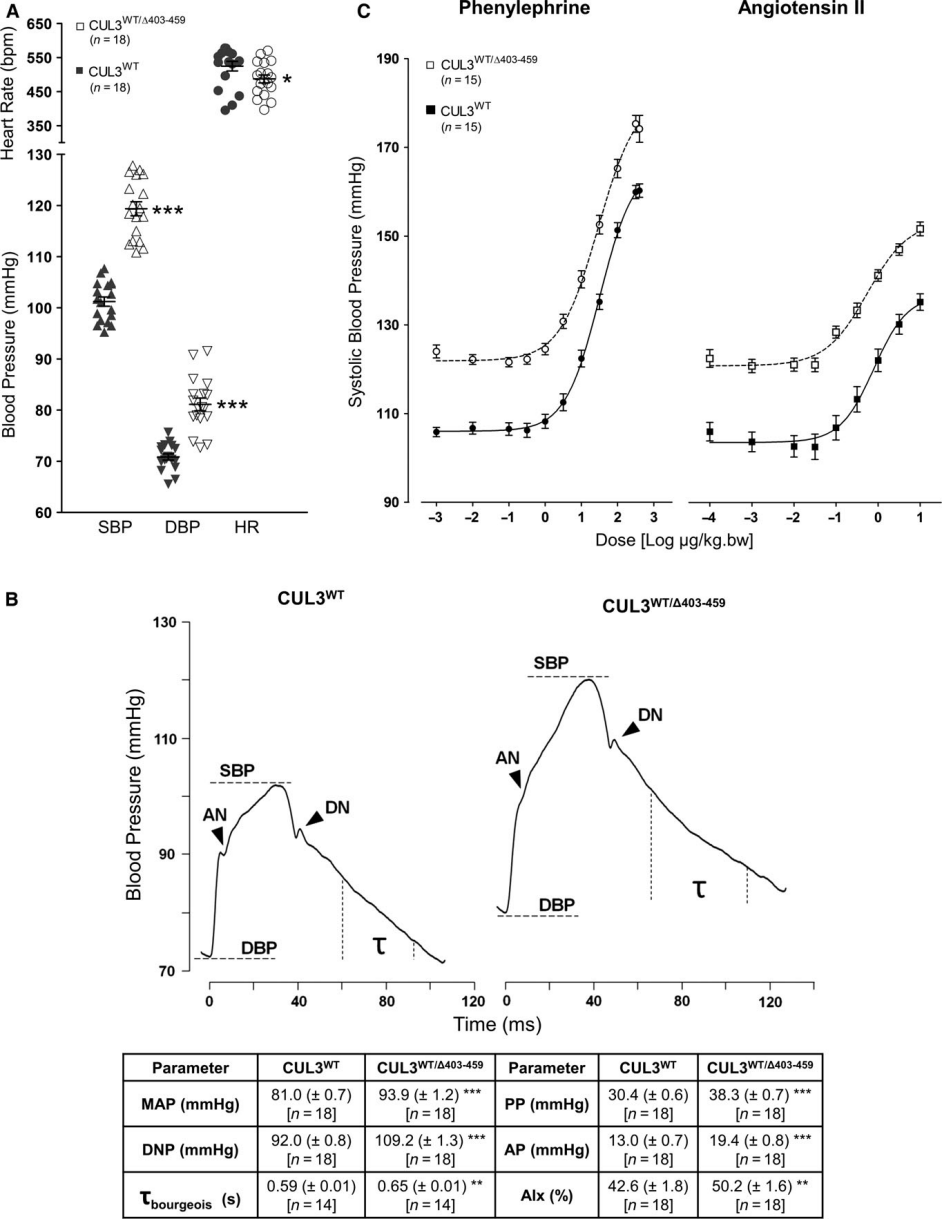
Increased arterial stiffness contributes towards the hypertension of CUL3^WT^^/Δ403–459^ mice Continuous blood pressure measurements were taken in mice under general anaesthesia with thermostatically controlled body temperature, right carotid artery pressure transducer catheterisation and ECG Lead II heart rate monitoring. CUL3^WT^^/Δ403–459^ mice have elevated systolic (SBP) (***P = 1 × 10^−12^) and diastolic (DBP) blood pressure (***P = 8.5 × 10^−8^), although they also present with 7% lower heart rates (*P = 0.0485) when compared to CUL3^WT^ mice. Two-tailed unpaired Student’s *t*-test; data are mean ± SEM.
Pulse waveform analysis of blood pressure traces obtained in (A) reveals that CUL3^WT^^/Δ403–459^ mice have an increased pulse pressure (PP) [SBP – DBP] (***P = 2.1 × 10^−10^), augmentation pressure (AP) [SBP – anacrotic notch (AN) pressure] (***P = 3.5 × 10^−6^), dicrotic notch (DN) pressure (***P = 4.7 × 10^−13^) and mean arterial pressure (MAP) [1/3 × SBP + 2/3 × DBP] (***P = 1.3 × 10^−9^). This hypertensive phenotype is in part due to changes in vascular contractility in CUL3^WT^^/Δ403–459^ mice as evidenced by their higher augmentation index (AIx) [AP/PP] (**P = 0.0096), a marker of arterial stiffness, and is further supported by an increase in their diastolic pressure decay time constant (τ_bourgeois_) [1/slope of diastolic pressure decay; measured 30 ms after DN and 20 ms before end DBP] (**P = 0.0083), a surrogate marker of increased vascular resistance. Two-tailed unpaired Student’s *t*-test; data are mean ± SEM
*In vivo* dose–responses to phenylephrine and angiotensin II. After baseline measurements were obtained from mice in (A), the right external jugular vein was cannulated for administration of bolus doses of vasopressors in increasing half-log steps. Dose–response curves were generated and data analysed using a logistical function to determine the estimated dose producing half-maximal response (ED_50_) and maximum response (*E*_max_). The fitted *E*_max_ for phenylephrine was increased in CUL3^WT^^/Δ403–459^ versus CUL3^WT^ (183.9 ± 2.5 versus 164.9 ± 1.4 mmHg) (***P = 1 × 10^−6^) indicating an increased vasoconstrictor response to adrenergic stimulation elevating systolic blood pressure substantially above the CUL3^WT^ maximum. Similarly, the fitted *E*_max_ for angiotensin II stimulation was higher in CUL3^WT^^/Δ403–459^ versus CUL3^WT^ (155.5 ± 1.8 versus 138.3 ± 2.2 mmHg) (***P = 1.7 × 10^−6^). However, there was no change between CUL3^WT^^/Δ403–459^ versus CUL3^WT^ sensitivity (as measured by ED_50_) to phenylephrine (36.6 ± 5.1 versus 31.4 ± 2.8 μg/kg bw) (*P *= 0.3778) or angiotensin II (0.77 ± 0.16 versus 0.93 ± 0.13 μg/kg bw) (*P* = 0.4401). Two-tailed unpaired Student’s *t*-test; data are mean ± SEM. Source data are available online for this figure.

Over-activation of the WNK pathway as observed in our CUL3^WT/Δ403–459^ mice (Figs 4 and 5) should lead to salt retention and elevated blood pressure. We therefore measured the blood pressure (BP) of the CUL3^WT/Δ403–459^ mice using a carotid pressure transducer catheter under general anaesthesia. Both male and female CUL3^WT/Δ403–459^ mice exhibited significantly higher BP relative to CUL3^WT/WT^ littermates (Fig 6A).

Strikingly, we also observed a previously unreported haemodynamic change in the blood pressure trace of the CUL3^WT/Δ403–459^ mice. The systolic aortic pressure wave of the heterozygous mice is augmented, while the diastolic relaxation (τ) is significantly slowed (Munir *et al*, 2007; Fig 6B). These changes suggest early wave reflection of the pressure wave and compliance changes consistent with a stiffened vascular tree. This stiffening could reflect a primary change in the contractile state of vascular smooth muscle, so to explore this we studied the *in vivo* vasoconstrictor responses to intravenous administration of phenylephrine and angiotensin II (Fig 6C; Bergaya *et al*, 2011). The resulting dose–response curves showed that the maximal constrictor response to both vasopressor agents was substantially higher in the CUL3^WT/Δ403–459^ mice.

### CUL3 and KLHL3 are expressed in the mouse and human aorta, and CUL3^WT/Δ403–459^ mice have aortic wall thickening

**Figure 7:**
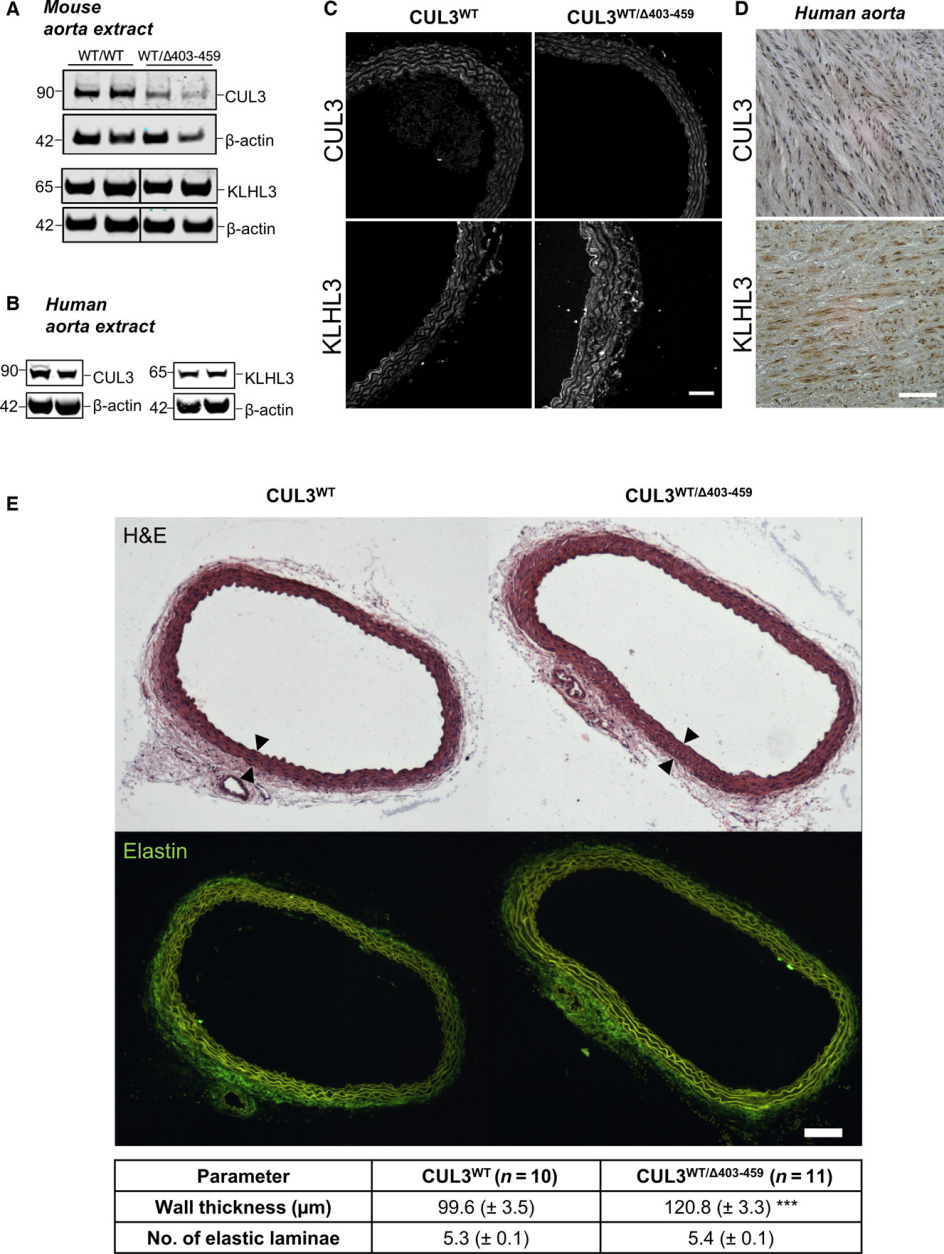
CUL3 and KLHL3 are present in mouse and human aorta, and CUL3^WT^^/Δ403–459^ mice undergo aortic vascular remodelling Western blot of tunica media-intima thoracic aorta lysates from mice culled after a minimum 4-h fast. Following exsanguination after surgery, mouse tissues were rapidly harvested and the tunica adventitia removed before storage, and later, the samples were homogenised, clarified and quantified prior to SDS–PAGE. Western blot analysis confirmed the expression of KLHL3 and CUL3. Similar to the kidney, the aorta of CUL3^WT^^/Δ403–459^ showed slightly lower levels of CUL3 compared to CUL3^WT^ without any change in KLHL3 levels.
Fresh frozen thoracic aorta tissues from donor cadavers were homogenised, clarified and quantified prior to SDS–PAGE. Western blot analysis confirmed the expression of KLHL3 and CUL3 in normal healthy human aorta.
Representative maximum-intensity *z* projections of immunofluorescently stained thoracic aorta sections showing the distribution of CUL3 and KLHL3 between CUL3^WT^^/Δ403–459^ and CUL3^WT^ mice at a minimum 4-h fasting baseline (*n *= 4 per genotype). CUL3 and KLHL3 localisation is comparable between genotypes. The highest levels were detected in the vascular smooth muscle cells and endothelium, with a minimal expression in the perivascular adipose tissue of the adventitia. Scale bar, 50 μm.
Representative immunohistochemical staining of KLHL3 and CUL3 in human thoracic aorta sections (*n *= 6). Similar to the mouse staining seen in (C), vascular smooth muscle cells in the tunica media of the aortic wall are positive for KLHL3 and CUL3. Scale bar, 50 μm.
Morphometric analysis of thoracic aortae reveals vascular remodelling in CUL3^WT^^/Δ403–459^ mice. There is an increase of ˜21% in the vessel wall intima-media thickness (demarcated by arrows) of CUL3^WT^^/Δ403–459^ compared to CUL3^WT^ mice (***P = 0.0003). However, there is no change in the number of elastin laminae (*P* = 0.1458) and therefore no increase in the number of medial muscle layers between genotypes. Two-tailed unpaired Student’s *t*-test; data are mean ± SEM. Source data are available online for this figure.

We also looked for evidence of biochemical changes in the vasculature and confirmed the expression of CUL3 and KLHL3 in mouse and human aorta by immunoblot analysis (Fig 7A and B, Appendix Fig S5). Similar to the kidneys (Fig 4A), no differences were seen in KLHL3 expression between CUL3^WT/Δ403–459^ and CUL3^WT^ mice, while the levels of CUL3 WT protein were lower in CUL3^WT/Δ403–459^ aorta. To address the altered contractility in the vessel wall *in vivo*, we also measured the level of pMYPT1 in aortic vessel lysates and showed a significant 1.7-fold increase (Feng *et al*, 1999; Somlyo & Somlyo, 2003; Appendix Fig S6). Image analysis of mouse and human aortae confirmed that both have strong CUL3 staining of the vascular endothelium and smooth muscle cells of the intimal and medial layers of the vessel wall (Fig 7C and D). Finally, to explore further the vascular phenotype, we performed a morphometric analysis of the aortae of CUL3 mice that confirmed significant thickening of the vessel wall in the heterozygote mice (Fig 7E). This probably reflects hyperplasia/hypertrophy of the vascular smooth muscle cells in the medial layer, as the numbers of elastic laminae were not significantly different (Fig 7E).

## Discussion

This study describes the first successful PHA2E mouse model involving CUL3 and highlights the molecular differences and defects of the mutant CUL3 protein. The deletion of 57 amino acids in CUL3^Δ403–459^ does not affect the ability of the bound RING domain to hydrolyse E2∼UB, and as such CUL3^Δ403–459^ is still an active E3 ligase. Instead, our structural predictions and experimental findings suggest that the CUL3^Δ403–459^ CRL scaffold lacks a level of rigidity required for substrate ubiquitylation. Our results are consistent with a previous report that demonstrated ubiquitylation of the Cullin-1 substrate (p27) was abrogated when a linker was inserted between the NTD and CTD of the closely related CUL1 protein (Zheng *et al*, 2002). Thus, the CUL3^Δ403–459^ mutation is a novel physiological example of the importance of this CRL rigidity and to our knowledge the first example of a human mutation that impedes the scaffolding function of a Cullin. Our data are consistent with the working hypothesis that the CUL3^Δ403–459^ mutation has increased structural flexibility. The deletion of three helices fuses together two unstructured regions, which likely allows greater movement between the N-terminal and C-terminal domains of the Cullin. This prevents the complex from successfully directing ubiquitin towards a bound substrate, and instead, leads to increased auto-ubiquitylation of CUL3 and KLHL3. Importantly, it appears as if *in vivo* this auto-ubiquitylation triggers degradation of the mutant form of the Cullin, as only relatively low amounts of CUL3^Δ403–459^ are detectable in tissue from the mouse model, while the levels of KLHL3 are unaffected. *In vivo*, CUL3 auto-ubiquitylation possibly precedes ubiquitylation of KLHL3, leading to rapid proteasomal degradation of CUL3^Δ403–459^ leaving KLHL3 untouched. The auto-degradation of CUL3 is likely to be the major driver for the associated phenotype in patients. While, some CUL3^Δ403–459^ remains in cells, we show that this remaining protein is unable to ubiquitylate WNK substrates. Taken together, our data suggest that the disease-associated deletion of exon 9 from CUL3 is a loss-of-function mutation with respect to substrate ubiquitylation. Importantly, all PHA2E patients are heterozygotes, so they retain a functioning copy of CUL3^WT^. Thus, the CUL3^Δ403–459^ mutation either behaves as a dominant-negative to inhibit CUL3^WT^ or is haploinsufficient in the context of blood pressure regulation with the single functional copy of CUL3 unable to sustain the physiological need for WNK ubiquitylation. Our data support a haploinsufficiency model, as a large proportion of the mutant protein is removed from the cell by auto-degradation and *in vitro* CUL3^Δ403–459^ does not inhibit CUL3^WT^. However, it is possible that *in vivo* the remaining CUL3^Δ403–459^ may still sequester KLHL3 from CUL3^WT^ in a dominant-negative fashion, as in cells we observed increased binding between CUL3^Δ403–459^ and KLHL3 (Fig 1H) and the reduced affinity for CAND1 may prevent the release of KLHL3 from non-functional CUL3^Δ403–459^ complexes. These two possibilities could be addressed in a heterozygous mouse model carrying a full-length CUL3 deletion; under the haploinsufficiency model, it should show WNK stabilisation.

The CUL3^WT/Δ403–459^ mice have high blood pressure and up-regulated signalling in the context of the WNK kinase pathway, which parallels the WNK4 (D568E; Yang *et al*, 2007; Chowdhury *et al*, 2013) and KLHL3 (R528H; Mori *et al*, 2013) PHA2 mouse models previously described. Mechanistically, our data suggest CUL3^Δ403–459^ promotes its own degradation, as it has a heightened propensity to self-ubiquitylate and is less abundant in mouse kidney extract. Hence, we would predict that PHA2E CUL3^WT/Δ403–459^ patients would similarly have lower overall CUL3 levels. As CUL3 ubiquitylates a number of other proteins, it seems remarkable then that PHA2E patients do not have other phenotypic manifestations. However, this may not be surprising if considered in the context of a haploinsufficiency model. BP homoeostasis requires reactive and precise control of ion fluxes in the distal nephron, especially the DCT, as changes in intake and output of salt are constantly balanced. The phosphorylation of the NCC/NKCC ion transporters by WNK kinases appears to be regulated by two complementary systems: the regulation of total WNK protein levels by CUL3-KLHL3, and the level of WNK activation by phosphorylation. Currently, the sensing mechanisms that lead to WNK phosphorylation or the degradation of the WNK kinases by CUL3-KLHL3 are largely unknown. However, sufficient amounts of CUL3 must be available for signalling-dependent ubiquitylation of the WNK kinases. It is possible that having only half the amount of functional CUL3 within kidney cells is not enough for the system to respond rapidly. The presence in our knock-in mice of WNK4 puncta *in vivo* also suggests that DCT cells may deploy secondary protein degradation pathways to manage excess WNK proteins, such as the autophagy–lysosomal system. However, the nuclear proximity of the large puncta and the absence of lysosomal staining or formation of ubiquitylated aggregates suggest that these excess WNK proteins are shunted to protein quality control compartments such as the aggresome or juxta-nuclear quality control compartment (JUNQ) in an attempt to compensate for loss of proteasomal degradation (Lamark & Johansen, 2012; Wolff *et al*, 2014). Hence, while other CUL3 substrates may be unaffected in the CUL3^WT/Δ403–459^ mice, the rapid and signal-dependent switching of WNK ubiquitylation may be executed abnormally in these mice and PHA2E patients.

Mutations in CUL3, KLHL3, WNK1 and WNK4 are known to cause FHHt with constitutive activation of the NCC co-transporter (Boyden *et al*, 2012; Osawa *et al*, 2013; Tsuji *et al*, 2013; Alessi *et al*, 2014; Glover *et al*, 2014). What has been less clear is why the mutation of CUL3 has resulted in a more severe form of FHHt (PHA2E). Our unexpected discovery of a vascular phenotype in the CUL3^WT/Δ403–459^ mice leads us to speculate that their hypertension may not be driven by salt retention in the DCT alone. It is possible that part of their hypertension originates from an increased contractile state in their vasculature tree. If a similar phenomenon occurs in PHA2E, this may explain the early onset and severity of the blood pressure that occurs in these patients. The CUL3^WT/Δ403–459^ mice have an altered aortic pulse waveform and slowed diastolic relaxation that is consistent with stiffening of their arterial tree. These changes have been reported before in humans with hypertension (Kaess *et al*, 2012), but not previously in a hypertensive mouse model. It is also worth noting that augmentation of the aortic pressure wave is often not detected by brachial cuff BP measurements, so it could have gone unnoticed in the routine clinical assessment of PHA2E patients. The increase in pMYPT1 in the aortae from the CUL3^WT/Δ403–459^ mice and the increase in their *in vivo* pressor responses to phenylephrine and angiotensin II further suggest that the altered contractile state could be a primary phenomenon rather than simply a secondary response to hypertension. If this is the case, the contraction could occur by at least two distinct pathways. Firstly, the WNK1/WNK3/SPAK/OSR1 pathway is thought to be important for regulating vascular tone by controlling the phosphorylation state of the NKCC1 co-transporter and hence the membrane potential of vascular smooth muscle (VSM) cells (Yang *et al*, 2010; Bergaya *et al*, 2011; Zeniya *et al*, 2013). So if either WNK1 or WNK3 accumulates in the vessel wall of the CUL3^WT/Δ403–459^ mice, the contractile tone would be expected to rise through depolarisation of VSM and increased calcium entry. Alternatively, the mutant Cullin-3 protein may directly affect the phosphorylation state of myosin light chain by regulating RhoA/RhoA kinase (ROCK) levels (Ibeawuchi *et al*, 2015). RhoA protein levels in the VSM are thought to be regulated by CUL3 in complex with the substrate adaptor RhoBTB1 (Pelham *et al*, 2012). If these pathways are operational in PHA2E, then they suggest that treatment with a thiazide diuretic or dietary sodium restriction, while effective in reversing the electrolyte disturbances, may be less effective in reversing any central aortic pressure changes in PHA2E. If WNK/SPAK/OSR1 is the predominant pathway causing disease, a loop diuretic such as bumetanide may be more effective than a thiazide, while for the RhoA/ROCK pathway a direct arterial vasodilator or a specific ROCK inhibitor (Liao *et al*, 2007) may be the more appropriate drug of choice. Central aortic pressure elevation is now widely accepted to be an important and independent cardiovascular risk factor (Liao & Farmer, 2014), so if it is elevated in patients with PHA2E, it will be important to show that it is normalised by appropriate pharmacotherapy.

## Materials and Methods

All plasmids, antibodies and recombinant proteins that we have generated for this study are available on request from the MRC-PPU reagents website (http://mrcppureagents.dundee.ac.uk/).

### Plasmids and protein purification

The following plasmids and/or protein purifications have been described elsewhere in Ohta *et al* (2013): KLHL3 (DU23218), DAC-TEV-CUL3-RBX1 (DU23291), His-UBE1 (DU32888) and His-TEV-UBE2D3 (DU15703); and in Kelsall *et al* (2013): Nedd8 (DU20689), UBE2M (DU15804) and APPBP1/UBA3 heterodimer (Nedd8 activating E1) (DU21784).

DAC-TEV-CUL3^Δ403–459^-RBX1 (DU23292) was cloned in a comparable way to DAC-TEV-CUL3^WT^-RBX1 (DU23291). Specifically, the pFastBac Dual DAC-TEV expression system was created by subcloning a BglII-BamHI-flanked PCR product encoding the full-length DAC-tag followed by a TEV protease site into a pFastBac™ Dual (Life Technologies, UK). Human Cullin-3 (Δ403–459; GenBank NM_003590.4) was codon-optimised for expression in insect cells and custom synthesised (GenScript USA Inc.) before being subcloned downstream of the DAC-tag in cassette two of this vector. Human RBX1 (GenBank NM_014248.2) was amplified from an EST IMAGE clone 3138751 and subcloned into cassette one (untagged). Human CAND1 (GenBank NM_018448.3) was amplified as a Not1 flanked ORF from EST IMAGE clone 5265409 and subcloned into pGEX6P-2 (GE Healthcare LifeSciences UK) for bacterial expression with an N-terminal GST tag. All PCRs were carried out using KOD Hot Start DNA Polymerase (Merck Millipore, Germany). All full-length products or fragments were cloned into pSc-B (Agilent) and sequenced in full prior to further subcloning or manipulation. DNA sequencing was performed by the Sequencing Service at the College of Life Sciences, University of Dundee (www.dnaseq.co.uk).

Human recombinant CUL3^Δ403–459^-RBX1 or CUL3^WT^-RBX1 was expressed in a multibac vector with a Dac-TEV-fusion tag on the CUL3 and untagged RBX1 (Lee *et al*, 2012). Proteins were expressed in the baculovirus system in Sf21 cells cultured in Insect-Protein-free Insect Cell Medium (Lonza). The fusion protein was captured from the lysate by incubation with ampicillin-sepharose. After washing, the protein was recovered from the solid phase by incubation with C-terminally His-tagged TEV protease (10 μg per 1 mg substrate). The protease and any free Dac-tag were removed with Ni-agarose and ampicillin–sepharose. The protein was then concentrated and further purified by size-exclusion chromatography (SEC) in 50 mM HEPES pH 7.5, 150 mM NaCl and 10% glycerol.

Human recombinant CAND1 was expressed as an N-terminally GST-tagged fusion protein in BL21 cells. Expression was induced with 50 μM IPTG at 15°C for 16 h. Following cell lysis, GST-CAND1 was captured on glutathione resin, washed and then eluted from the resin with 10 mM reduced glutathione in 50 mM HEPES pH 7.5, 150 mM NaCl, 10% glycerol (w/v) and 1 mM DTT. The protein was then dialysed to remove glutathione.

Nedd8 was expressed as an untagged protein in BL21 cells, and following induction with 1 mM IPTG at 16°C, cells were lysed and frozen. Upon thawing, benzonase and 2 mM Mg(C_2_H_3_O_2_)_2_ were added and cells were disrupted by sonication. Insoluble material was pelleted, and this pellet was resuspended in 50 mM Tris pH 7.5 and 150 mM NaCl and washed thrice. To extract Nedd8, the pellet was resuspended in 8 M urea and mixed overnight. Following clarification by centrifugation the supernatant was diluted fourfold with MQ water and dialysed thrice to remove urea and enable Nedd8 refolding. Contaminants were removed by depletion using a Q-sepharose column. The Nedd8 was concentrated prior to a final purification by SEC in 20 mM HEPES pH 7.5, 150 mM NaCl and 0.03% Brij 35.

### Antibodies

The following antibodies were raised in sheep and affinity-purified on the appropriate antigen by the Division of Signal Transduction Therapy Unit at the University of Dundee: WNK1-total antibody (residues 2,360–2,382 of human WNK1, S62B), WNK4 N-terminal antibody (residues 1–14 of mouse WNK4, S726D), SPAK-mouse N-terminal antibody (2–76 of mouse SPAK, S668D), SPAK-mouse C-terminal antibody (424–556 of mouse SPAK, S669D), SPAK/OSR1 (T-loop) phospho-Thr233/Thr185 antibody (226–238 of human SPAK or residues 178–190 of human OSR1, TRNKVRKpTFVGTP, S204C), SPAK phospho-Thr233 antibody (226–238 of human SPAK, TRNKVRKpTFVGTP, S668B and S668D), NKCC1-total antibody (residues 1–260 of shark NKCC1, S841B), NCC phospho-Thr60 antibody (residues 54–66 of human NCC phosphorylated at Thr60, RTFGYNpTIDVVPT, S995B), NCC phospho-Thr44 antibody (residues 38–52 of mouse NCC phosphorylated at Thr44, SQPSHLTpHGSTLYMRRR, S242C) and KLHL3 N-terminal mouse (1–21 of mouse KLHL3, S740D). The anti-ERK1/2 antibody (9102) was purchased from Cell Signaling Technology. The anti-FLAG antibody (F1804), anti-β-actin antibody and anti-His antibody were purchased from Sigma-Aldrich. The rabbit anti-human CUL3 antibody was a kind gift from Izabela Sumara at the IGBMC, Strasbourg (Sumara *et al*, 2007). The following commercial rabbit anti-human antibodies were obtained and used for immunodetection: KLHL3 antibody (HPA051291) from Atlas Antibodies; KLHL3 (AB196776) (∼14% immunogen peptide homology with KLHL2), NCC-total [SLC12A3] (AB95302) and LAMP1 (AB24170) from Abcam; and PV25 (PVALB) from Swant. The goat anti-GFP (AB5450) and the mouse monoclonal anti-ubiquitin (AB7254) were purchased from Abcam. The MYPT1 phospho-Thr696 mouse monoclonal antibody (MAB0001) was obtained from Abnova. Secondary antibodies coupled to horseradish peroxidase used for immunoblotting were obtained from Pierce. Secondary antibodies conjugated to fluorochromes for LiCor Odyssey Western blot scanning were obtained from Licor (www.licor.com) and Life Technologies. Fluorochrome-conjugated secondary antibodies for immunofluorescent confocal microscopy were obtained from Life Technologies and Abcam.

### *In vitro* assays

Ubiquitin E2 discharge assays were performed based on the method described (Plechanovová *et al*, 2011). Firstly, His-UBE2D2 (150 μM) was incubated in the presence of 2 mM ATP, 5 mM MgCl_2_, 200 μM ubiquitin and 0.25 μM UBE1 in 50 mM HEPES pH 7.5, 150 mM NaCl, 10% glycerol and 1 mM DTT at 16°C for 15 min. To stop the reaction, we depleted ATP by incubating the charging mixture with apyrase (4.5 U/ml; New England Biolabs) at 16°C for 10 min. To this, 1 μM E3 was added and discharge reactions were then allowed to proceed for the time indicated in the figure, at 30°C. Reactions were stopped by the addition of non-reducing SDS–Laemmli sample buffer. 16% bis-acrylamide non-reducing SDS–PAGE, followed by immunodetection with anti-His antibody, allowed visualisation of ubiquitin discharge. E3 ubiquitin-ligase activity assays were based on those described previously in Ohta *et al* (2013). When used as a substrate in assays, full-length WNK kinases were purified from HEK-293 cells, and either 5 μl of immunoprecipitated endogenous WNK1 protein derived from 0.5 mg of HEK-293 cells or 5 μl of immunoprecipitated over-expressed FLAG-WNK4 from 0.05 mg of HEK-293 cells was used for each assay reaction. Ubiquitylation assays typically contained 20 mM HEPES/HCl (pH 7.5), 150 mM NaCl, 2 M DTT, 10% (w/v) glycerol, 8 μM CUL3-RBX1 complex (WT or mutant), 7 μM KLHL3, 7 μM UBE1, 60 μM UBE2D3 and 3,000 μM ubiquitin. The concentrations of some components were altered on occasion (e.g. Fig 3B) to best illustrate a given point. Reactions were initiated by adding ATP and MgCl_2_ to a final concentration of 1 mM, and samples were incubated for the times indicated at 30°C. Reactions were stopped by the addition of SDS sample buffer, and samples were analysed after SDS–PAGE followed by immunoblotting or Coomassie blue gel staining. For the N8-ligase activity assays, 1 μM E3 was incubated at 30°C with 68 μM Nedd8, 8 μM UBE2M and 0.2 μM NAE in the presence of 0.15 mM ATP, 1.5 mM MgCl_2_ 50 mM HEPES pH 7.5, 150 mM NaCl and 20% (w/v) glycerol for the time indicated. Reactions were stopped by the addition of SDS sample buffer, and samples were analysed following SDS–PAGE separation and Coomassie blue staining.

### *In vitro* protein interaction pull downs

For KLHL3-CUL3, anti-KLHL3 resin was made by covalently coupling anti-KLHL3 antibody (S377D) to Protein G sepharose utilising dimethyl pimelimidate. Anti-KLHL3 resin, or control resin (prepared in the absence of any antibody), was incubated with saturating amounts of purified KLHL3 and washed thrice, resulting in KLHL3 resin. This resin was then incubated with CUL3^WT^ or CUL3^Δ403–459^ at 4°C for 1 h, before being washed thrice with PBS 0.02% Tween-20. For CAND1-CUL3, purified GST-CAND1 or GST was incubated in excess with resin at 4°C resulting in GSH-resin-GST or GSH-GST-CAND1 resin. Each resin was then incubated with purified CUL3^WT^ or CUL3^Δ403–459^ for 1 h at 4°C before being washed once with PBS 0.02% Tween-20 and then twice with PBS in the absence of detergent. Following washing, volumes were reduced to a minimum and proteins were eluted from the resin by the addition of SDS sample buffer, prior to analysis by SDS–PAGE and immunoblotting.

### Cell culture

HEK (human embryonic kidney)-293 cells were cultured in 14-cm dishes in DMEM (Dulbecco’s modified Eagle’s medium; Life Technologies) supplemented with 10% (v/v) foetal bovine serum, 2 mM l-glutamine, 100 units/ml penicillin and 0.1 mg/ml streptomycin. Protein expression was induced for 24 h with 1 μg/ml tetracycline (Life Technologies). To obtain endogenous WNK1 for activity assays, we followed the immunoprecipitation protocol described in Ohta *et al* (2013). To obtain wild-type FLAG-WNK4, we utilised the cell lines from Schumacher *et al* (2014) and Ohta *et al* (2013). When FLAG-WNK4 was to be used for *in vitro* ubiquitylation assays, a 14-cm plate of confluent cells was lysed in 0.5 ml ice-cold mammalian lysis buffer A (mammalian lysis buffer A: 50 mM Tris/HCl (pH 7.5), 0.15 M NaCl, 1 mM EGTA, 1 mM EDTA, 1 mM Na_3_VO_4_, 50 mM NaF, 5 mM Na_4_P_2_O_7_, 0.27 M sucrose, 1% (w/v) Nonidet P-40, 1 mM benzamidine, 0.1 mM PMSF, 0.1% 2-mercaptoethanol, and Roche protease inhibitor mix (1 tablet in 50 ml) for M2 affinity purification). Lysates were clarified by centrifugation, and to this, 10 μl M2 resin per 100 μl lysate was added. Incubation of the resin with the cellular lysate for 1 h at 4°C was followed by two washes with mammalian lysis buffer A, and two further washes with 1× PBS to give a pure M2-Flag-WNK4 slurry. For interaction studies with FLAG-CUL3^WT^ and FLAG-CUL3^Δ403–459^, the same method was followed as above, with an alternative mammalian lysis buffer B (50 mM HEPES/KOH pH 7.2, 5 mM Mg(C_2_H_3_O_2_)_2_, 70 mM KC_2_H_3_O_2_, 0.2% (w/v) Triton X-100, 10% (w/v) glycerol, 0.2 mM EDTA, 1 mM Na_3_VO_4_, 50 mM NaF, 5 mM Na_4_P_2_O_7_ and Roche protease inhibitor mix (1 tablet in 50 ml)). When it was desirable to prevent deneddylation, buffers were supplemented with 50 μM 1,10-phenanthrolin.

### Mass spectrometry analysis

Prior to SDS–PAGE, samples were reduced and alkylated in the following way. Reactions were stopped at the time shown and incubated at 95°C in 1× LDS (Invitrogen) 5 mM DTT for 1 min. Alkylation occurred at room temperature for 30 min by the addition of 20 mM iodoacetamide (20 mM) to the samples. To quench the reaction, DTT was added to a final concentration of 20 mM, and the sample was then processed on precast 4–12% gradient gel (Invitrogen). Gel pieces were excised as shown by the boxed areas in the figure and in gel digestion of the proteins with 5 μg/ml trypsin and subsequent analysis. Mass spectrometric analysis was performed by LC-MS-MS on a linear ion trap–orbitrap hybrid mass spectrometer (Orbitrap-VelosPro; Thermo) coupled to a U3000 RSLC HPLC (Thermo). Peptides were trapped on a NanoViper trap column, 2 cm × 100 mm C18 5 mm 100 Å (Thermo, 164564) and then separated on a 15-cm Thermo EasySpray column (ES800) equilibrated with a flow of 300 nl/min of 3% solvent B [solvent A: 2% acetonitrile, 0.1% formic acid and 3% DMSO in H_2_O; solvent B: 80% acetonitrile, 0.08% formic acid and 3% DMSO in H_2_O]. The elution gradient was as follows: time (min): solvent B (%): 0:3, 5:3, 45:35, 47:99, 52:99, 55:3 and 60:3. The instrument was operated with the “lock mass” option to improve the mass accuracy of precursor ions, and data were acquired in the data-dependent mode, automatically switching between MS and MS-MS acquisition. Full-scan spectra (m/z 400–1,600) were acquired in the orbitrap with resolution *R* = 60,000 at m/z 400 (after accumulation to an FTMS Full AGC Target, 1,000,000; FTMS MSn AGC Target, 50,000). The 20 most intense ions, above a specified minimum signal threshold (2,000), based upon a low-resolution (*R* = 15,000) preview of the survey scan, were fragmented by collision-induced dissociation and recorded in the linear ion trap (Full AGC Target, 30,000; MSn AGC Target, 5,000). Data files were analysed by Proteome Discoverer 1.4-SP1 (Thermo), using Mascot 2.4.1 (www.matrixscience.com), and searching an in-house database containing the relevant sequences. Scaffold (www.ProteomeSoftware.com) was also used to examine the Mascot result files. Allowance was made for the following fixed, Carbamidomethyl (C), and variable modifications, Oxidation (M), Dioxidation (M), GlyGly (K), LeuArgGlyGly (K) and Phospho (ST). Error tolerances were 10 ppm for MS1 and 0.6 Da for MS2.

### Generation and genotyping of WT and CUL3^Δ403–459^ mice

Standard housing conditions were used to maintain the mice. Taconic Artemis generated the CUL3^Δ403–459^ mice on a C57BL/6N background, which were backcrossed onto C57BL/6J to maintain the colony. All experiments were performed using littermates that were backcrossed at least once onto C57BL/6J. Genomic DNA from ear biopsies was isolated and used to genotype all mice in the study. Genotyping by PCR utilising primer 1: AAACTTACCCACTTGTTTGCC, and primer 2: AGACATCTCAGGTTACTATGGGC detected the presence of the CUL3^WT^ (683 bp) or CUL3^Δ403–459^ (395 bp) allele. Studies were conducted on groups of approximately equal ratios of male-to-female mice with the exception of *in vivo* cardiovascular phenotyping in which a 1:2 ratio of male-to-female mice was used. All animal studies and breeding were approved by the University of Dundee ethical committee and performed under a UK Home Office-approved licence.

### Mouse kidney and testes lysates

Mouse tissues were rapidly harvested and either snap-frozen in liquid nitrogen and stored at −80°C or incubated in 1 ml RNAlater® solution (Sigma-Aldrich) at 4°C for 16–30 h before being removed from this buffer, patted dry with clean tissue and then stored dry at −80°C. Lysates were prepared by homogenising tissue on ice using a Polytron PT1200C homogenizer (Kinematica) in 2 ml ice-cold mammalian lysis buffer and then clarified by centrifugation at 18,000 *g* for 30 min. The clarified supernatant was then quantified and used immediately. Any excess sample was snap-frozen in liquid nitrogen in single-use aliquots for use as required.

The lysates to be used for immunoprecipitation were prepared in the same way, with the following exceptions, all designed to promote deneddylation of the cullin proteins. Detergent was not included in the lysis buffer. Following homogenisation, samples were incubated on ice for 4 h. Following clarification by centrifugation at 18,000 *g* for 30 min, samples were aliquoted into fractions and either frozen in liquid nitrogen and stored at −80°C or used immediately. Clarified supernatant was incubated at room temperature for 1 h to enable further deneddylation, and samples were then cooled on ice and centrifuged a second time to ensure any precipitate was removed. Anti-CUL3 antibody (S067D) cross-linked (DMP) to Protein G agarose was mixed with saturating amount of lysate and incubated rotating overnight at 4°C. The next morning, the agarose beads were washed thrice with mammalian lysis buffer B and twice with 1× PBS. Proteins were eluted from beads by SDS sample buffer addition and boiling at 95°C for 10 min prior to centrifugation and SDS–PAGE and immunodetection.

### Immunoblotting

Lysates or protein mixes were boiled at 95°C with 1× SDS–Laemmli sample buffer, for 5 min before being subject to SDS–PAGE (8 or 10% Tris–glycine gel, or 4–10% gradient gels (Life Technologies) or self-prepared 16% bis-acrylamide). Gels were transferred to nitrocellulose membrane using the standard wet transfer method (15% MeOH Towbin transfer buffer). Following blocking of the membranes (TBST 5% (w/v) dried skimmed milk), they were then incubated overnight at 4°C with the primary antibody indicated. All DSTT-produced sheep antibodies were used at 2 μg/ml, and phospho-specific antibodies included the addition of 10 μg/ml of the dephospho-peptide used to raise the antibody. Commercial antibodies were diluted 1 in 1,000. Membranes were then washed in TBST five times and incubated with the appropriate secondary HRP antibody at room temperature at 1:5,000 dilution. Membranes were subsequently washed five times, and the HRP signal was detected using chemiluminescence reagent (Pierce). Immunoblots were developed using an automatic film processor (SRX-101; Konica Minolta Medical).

### CUL3 structural docking

Cullin-3 was modelled using Phyre2 server (Kelley & Sternberg, 2009) using human CUL3^Δ403–459^ as the sequence input and Cullin-1 chosen for the docking model (Q13616, PDB:1LDK; Zheng *et al*, 2002). A coil was predicted in place of the missing structural elements. UBE2D∼UB was docked to RBX1 using PDB: 4AP4. Images were modified and made in UCSF Chimera (Pettersen *et al*, 2004; http://www.cgl.ucsf.edu/chimera).

### Immunoprecipitation (mouse tissue and cell extracts)

Lysates of cellular preparations (buffer B) from FLAG-tagged CUL3 were mixed with M2 resin in the presence of 50 μM 1,10-phenanthrolin to prevent deneddylation and were incubated rotating at 4°C for 1 h. Resins were washed twice with mammalian buffer B and twice with 1× PBS, the volume was then reduced to ∼15 μl, and following the addition of SDS gel sample buffer to 1×, samples were boiled and the entire sample was loaded to allow analysis by SDS–PAGE and immunodetection with the antibodies indicated.

### Human and mouse aorta sample preparation

Human aortic tissues were collected from donors through transplant coordinators at Addenbrooke’s Hospital, Cambridge. All samples were handled in accordance with the policies and procedures of the Human Tissue Act and with the approval of the Local and Regional Ethics Committees. Mouse tissues were rapidly harvested, perivascular fat and residual blood were removed, and the samples were incubated in 1 ml RNAlater® solution at 4°C for 16–30 h before being removed from this buffer and stored dry at −80°C. Frozen aortic tissues were homogenised using TissueLyser LT (Qiagen #85600), and protein lysates were extracted in NE-PER (Life Technologies #78833) lysis buffers containing protease and phosphatase inhibitors (Roche #11836170001 and #04906845001). All steps were carried out at 4°C. Protein concentrations were determined with the Pierce™ BCA protein assay (Life Technologies #23225).

### Human and mouse aorta immunoblotting

A total of 10 μg of aortic protein lysates was separated by SDS–PAGE. Prior to loading, samples were heated at 70°C for 10 min in 1× LDS sample buffer and 1× reducing agent (Life Technologies #B0007 and # B0009) to a total volume of 20 μl/well. Samples and 5 μl of Precision Plus molecular weight ladder (Biorad #161-0374) were then loaded onto a Bolt® 4–12% Bis–Tris Plus Gel (Novex® #BG04125BOX) and run at 165 V in 1× MES running buffer (Novex® #B0001), for 35 min or until the dye front reached the end of the gel. Resolved proteins were then transferred to 0.22 μM nitrocellulose membrane (Life Technologies #IB23001) using the iblot2 (Life Technologies) at 20 V for 7 min. Antibodies were incubated in 5% milk. Membranes were incubated in 5% in milk or BSA in TBS for 1 h at room temperature. Primary antibodies were incubated overnight at 4°C in 5% milk or BSA in TBS–Tween (0.1% v/v Tween) and then washed 6× in TBS–Tween. Secondary antibodies were incubated in TBS–Tween for 1 h at room temperature in the dark and then washed 6× in TBS–Tween. Membranes were imaged and integrated intensity values quantified (bands were normalised against β-actin) using the LiCor Odyssey system (www.licor.com).

### Sample preparation and immunostaining for imaging

Formal-fixed, paraffin-embedded human tissue sections were obtained from the Cambridge Human Research Tissue Bank. Harvested mouse tissues were immersion-fixed in fresh 4% w/v formaldehyde–PBS pH 6.9 for 16 h at 37°C and washed 3× in PBS and stored at 4°C until paraffin embedding. Five-micrometre sections were deparaffinised in Histoclear (National Diagnostics) and rehydrated in graded methanol steps. An antigen retrieval step was performed with R-Universal buffer in the 2100 antigen retriever for a single heat–pressure cycle (Aptum Biologics). Sections were permeabilised with 0.05% v/v Triton X-100–PBS for 20 min and blocked for 1 h at 37°C with 2% v/v donkey serum in 0.05% v/v Triton X-100–PBS. Primary antibodies were incubated overnight for 16 h at 4°C at the following concentrations diluted in 1% v/v donkey serum in 0.05% v/v Triton X-100–PBS: 2 μg/ml for pNCC T44, tNCC, CUL3 (Sumara *et al*, 2007); 4 μg/ml for KLHL3 N-terminal, WNK4 N-terminal, SPAK C-terminal and pSPAK T233 (to detect mouse pSPAK T243); and 1 μg/ml for LAMP1, 1:500 for ubiquitin and 1:2,000 for PVALB. Phospho-specific antibodies included the addition of 10 μg/ml of the non-phospho form of the peptide used to raise the antibody per 2 μg/ml of antibody used. Slides were then washed for 20 min in 0.05% v/v Triton X-100–PBS and incubated in secondary antibody for 1 h at 37°C. Pre-absorbed donkey IgG-conjugated Alexa Fluor 488, 568, 633 and 647 secondary antibodies (Life Technologies/Abcam) were used at 1:200 dilution in 1% v/v donkey serum in 0.05% v/v Triton X-100–PBS for immunofluorescent labelling. Slides were washed as described above, mounted using Prolong Gold Antifade (P36930; Life Technologies) and shielded from light. Human IHC DAB staining was performed with ImmPRESS Reagent peroxidase universal anti-mouse/rabbit IgG (MP-7500, Vector) kit, after which slides were then dehydrated through graded methanol steps and mounted with DPX.

### Image acquisition and processing

Immunofluorescence images were acquired on the Leica TCS SP2 laser scanning confocal microscope with 488-, 543- and 633-nm laser lines mounted on an upright Leica DM RXA fluorescence microscope using a HCX PL APO 63×/1.40NA oil immersion objective. Acquisition parameters were as follows: 12-bit, 1,024 × 1,024 pixels, 2.6× digital zoom, 800 Hz scan speed, 4-line Kalman filtering, sequential (by line) channel imaging, and 10-slice *z*-stack of 5 μm. Immunohistochemically stained specimens were imaged in brightfield on an Olympus BX51 upright epifluorescent microscope using a UPLANFL 20×/0.5NA dry objective with the Infinity3 (Lumenera) CCD set to 1,936 × 1,456 pixels.

In FIJI image analysis software (http://fiji.sc/Fiji), fluorescent *z*-stacks underwent background subtraction (1,000-pixel-radius rolling ball, no smoothing) and maximum-intensity *z*-projection. Brightness and contrast were adjusted by linear histogram stretching to enhance visibility. Any images to be compared to one another were processed in parallel. Similarly, brightfield images underwent linear histogram stretching to increase visibility uniformly across the image.

### Morphometric analysis

Body weight was regularly measured two times per week throughout the study to determine differences in mass in age-matched mice. Body length was measured using photographs of mice post-mortem with a reference scale. Full body length was determined as well as tail:body length ratio. Three 5-μm sections of thoracic aorta were taken at upper (heart), mid and lower (near diaphragm) segments per mouse. These were deparaffinised, rehydrated and stained with haematoxylin and eosin, and then dehydrated and mounted. Each section was imaged under brightfield illumination and epifluorescent imaging of autofluorescent elastin laminae with a GFP filter set. Using FIJI image analysis software, macros were scripted to draw eight lines at ∼22.5° to one another that intersect the aortic ring at a total of 16 points. At the point of intersection on the outer circumference, the distance from the external lamina to the inner lumen was measured. The average of the three rings (max of 16 points per ring, with points excluded if they fell on an arterial branching point or incomplete portion of a ring) was used to calculate the average thoracic aorta intima-media wall thickness. The above step was repeated to count the average number of elastic laminae in the thoracic aorta of each mouse. Data collection and analysis were carried out in a blinded fashion throughout.

### *In vivo* cardiovascular physiology

Animals were anaesthetised with isoflurane on 100% O_2_ (induction: 3%, maintenance: 1.75%) and placed on a self-regulating rectal probe-coupled heat mat (TC-1000; CWE) to maintain a body temperature of 37°C. Heart rate was measured by the R-R wave interval from ECG Lead II using an animal bio amp (FE136; AD Instruments) with needle electrodes inserted into fore and hind limbs (MLA1213; AD Instruments). The right carotid artery was catheterised with a 1F Mikro-Tip pressure transducer (SPR-1000; Millar) connected to a bridge amp (FE221; AD Instruments) and powerlab system (PL3504/P; AD Instruments). When the animals had stabilised, measurements were taken at 2,000 samples/s using LabChart version 7/8 Pro (AD Instruments) to record ECG and blood pressure pulse waveforms. *In vivo* dose–responses to vasopressor agents were taken after baseline blood pressure traces were obtained from mice. The right external jugular vein was cannulated for administration of bolus doses in increasing half-log steps of μg/kg body weight (bw); initially, phenylephrine was administered, and after washout and return to baseline, angiotensin II was administered in a similar fashion. Each dose concentration was made up as a half-log serial dilution in sterile 0.9% w/v saline and administered at a volume of 0.2 ml/kg.bw with an accompanying 20 μl 0.9% w/v saline cannula flush between doses.

Data processing and analysis were performed in LabChart 8 Pro. Using the blood pressure add-on, systolic, diastolic and dicrotic notch blood pressures were automatically detected per beat (beats with respiratory-induced artefact were gated out of the analysis using the beats classifier). Mean arterial pressure was calculated as (1/3 peak systolic pressure + 2/3 end diastolic pressure) and pulse pressure as (peak systolic pressure – end diastolic pressure). A macro was scripted to detect the anacrotic notch, by using the third zero value crossing the fourth derivative of the blood pressure, as described in Kelly *et al* (1989). The augmentation pressure was calculated as (peak systolic pressure – anacrotic notch pressure) and augmentation index as (augmentation pressure/pulse pressure). A macro was scripted to measure the slope of the diastolic pressure decay, 30 ms after the dicrotic notch and 20 ms before the end diastolic pressure (to avoid perturbations caused by aortic valve opening/closing). The reciprocal of this slope (ignoring the sign and calculated from the 20% trimmed mean values) was used to determine the time decay constant of the diastolic pressure decay which correlates with vascular resistance, as described previously (Bourgeois *et al*, 1974). Dose–response curves were generated and data analysed (GraphPad Prism 5 and LabChart 8 Pro) using a 4-parameter logistical function to determine the estimated dose producing half-maximal response (ED50) and maximum response (Emax). Data collection and analysis were carried out in a blinded fashion throughout. Sample sizes for the mouse work were decided based on previous experience in a related mouse model (Rafiqi *et al*, 2010). When groups of animals were being compared, procedures were performed on animals selected at random from the groups.

### Blood and urine analytes

To reduce variability introduced by dietary potassium ingestion, animals were fasted for a minimum of 4 h before being anaesthetised with isoflurane on 100% O_2_ (induction: 3%, maintenance: 1.75%) and placed on a self-regulating rectal probe-coupled heat mat (TC-1000; CWE) to maintain a body temperature of 37°C. To minimise air contact of arterial bloods during measurements, the right carotid artery was cannulated with a mouse arterial catheter (MAC-02; SAI Infusion Technologies) pre-flushed with 10 U heparin-0.9% w/v saline to prevent clots. Blood was released via the catheter into the EC8+ cartridge for analysis on the iSTAT (Abaxsis). Post-surgery additional blood was collected by using Microvette® CB 300 LH (Sarstedt) and centrifuged at 2,000 *g* for 5 min to extract plasma before storage at −80°C. Plasma aldosterone was calculated by HTRF (homogeneous time-resolved fluorescence) aldosterone assay (Cisbio, Codolet, France), according to the manufacturer’s protocol using the PheraStar FS (BMG Labtech) plate reader.

To determine plasma and urine electrolyte homoeostasis, animals were placed on a 0.3% w/w Na diet for 2 weeks with urine and plasma time-matched samples collected between days 7 and 10 for normal Na (NNa) diet baseline. On day 14, mice were switched onto a 0.03% w/w Na diet with sampling repeated on days 7–10 for low-Na (LNa) diet measurements. Spot urine was collected from awake mice following spontaneous micturition on handling over a sheet of Saran® wrap or Parafilm®. Samples were then divided, and one sample received acidification with HNO_3_ to a final concentration of 1% v/v to prevent precipitation of electrolytes, before both were stored at −80°C. Blood was collected by saphenous venepuncture in awake restrained animals, and plasma was separated and stored in a similar fashion to that described above. Plasma and urine (non-acidified) creatinine levels were assayed in the Core Biochemical Assay Laboratory, Addenbrooke’s Hospital, Cambridge, UK. Plasma and urine (acidified) samples were diluted 1:1,000 using ultrapure polished water containing 1% v/v HNO3. Cations were then measured by inductively coupled plasma-optical emission spectrometry (Perkin Elmer ICP-OES Analyser) with known concentration standards and preset elemental spectra. Data collection and analysis were carried out in a blinded fashion throughout.

### Statistical analysis

Data presented are mean ± standard error of the mean (SEM), unless otherwise stated. Statistical analyses were performed on either SPSS version 22 or GraphPad Prism® (http://www.graphpad.com/scientific-software/prism/) software. Two-tailed Student’s *t*-tests were performed either paired or unpaired where appropriate. *P* < 0.05 was taken as statistically significant throughout.

For MYPT1 phosphorylation quantification (Appendix Fig S6), ratiometric expression was calculated for CUL3^WT/Δ403–459^ versus CUL3^WT^ on each Western blot of independent biological replicates. The mean of the ratios and bounds of the 95% confidence interval were calculated, where ratio = 1 represents no change in phosphorylation and ratio > 1 represents an increase in the phosphorylation status of CUL3^WT/Δ403–459^ mice. Statistical significance was determined by the ratio *t*-test (http://www.wormbook.org/chapters/www_statisticalanalysis/statisticalanalysis.html).

## For more information

All of the constructs generated in this study, and many of the purified proteins and antibodies can be purchased at the following link: https://mrcppureagents.dundee.ac.uk

MRC-PPU Kurz webpage: http://www.ppu.mrc.ac.uk/research/?pid=1004

EMIT O’Shaughnessy webpage: http://emit.medschl.cam.ac.uk/

For more information regarding the human tissue utilised in this study please go to http://www.cuh.org.uk/tissue-bank/about

OMIN disease database: http://www.omim.org/entry/145260

Protein structures mentioned in this study can be found under the noted PDB identifier online at http://www.rcsb.org

## Supporting Information

Appendix

Expanded View Figures PDF

Review Process File

Source Data for Figure 7

Source Data for Expanded View and Appendix
