# Supplementary material for: Characterisation of the Cullin-3 mutation that causes a severe form of familial hypertension and hyperkalaemia
Source: EMBO Mol Med. 2015 Aug 18;7(10):1285–306. doi: 10.15252/emmm.201505444 (PMC4604684; doi:10.15252/emmm.201505444)
Supplement: Supplementary file 1 [file emmm0007-1285-sd1.pdf]

Appendix

Characterisation of the Cullin-3 mutation that causes a severe form of familial hypertension and hyperkalaemia

Frances-Rose Schumacher[1*], Keith Siew[2*], Jinwei Zhang[1], Clare Johnson[1], Nicola Wood[1], Sarah E Cleary[2], Raya S Al Maskari[2], James T Ferryman[2], Iris Hardege[2], Yasmin[2], Nichola L Figg[3], Radoslav Enchev[4], Axel Knebel[1], Kevin M O'Shaughnessy[2] and Thimo Kurz[1]

[1] MRC Protein Phosphorylation and Ubiquitylation Unit, College of Life Sciences, University of Dundee, Dow Street, Dundee DD15EH, Scotland, UK.
[2] Divisions of Experimental Medicine and Immunotherapeutics and [3] Cardiovascular Medicine, Department of Medicine, University of Cambridge, Cambridge CB2 2QQ, UK.
[4] Institute of Biochemistry, ETH Zürich, Otto-Stern-Weg 3, CH-8093 Zürich, Switzerland

Content:

Appendix Figure S1
Appendix Figure S2
Appendix Figure S3
Appendix Figure S4
Appendix Figure S5
Appendix Figure S6
Appendix Table S1

Appendix Figure S1.

A. Residues encoded for by exon-9 mRNA of Cullin3 are conserved in Cullin1. A Clustal-Omega alignment of full length Cullin1 and Cullin3 was performed, the region shown equates to that encoded by exon-9 mRNA in Cullin3 and highlights the similarity between these two proteins at this region.
B. Structural model of CUL3^{WT} (upper) and CUL3$^{\Delta 403\text{-}459}$ (lower) made based on the structure of full length Cullin1 (1LDK) using Chimera (see methods). The NTD is coloured mauve, the CTD is coloured cyan and the region deleted in CUL3$^{\Delta 403\text{-}459}$ is coloured grey in the CUL3^{WT} model.

Appendix Figure S2.

In vitro ubiquitylation assays as described in Figure 1-3**.**
A. The entire coomassie SDS PAGE (uncropped) are shown in this figure, along with additional reactions to support those in the main document.
B. Entire coomassie stained SDS PAGE of Figure 1E in main text. As described in Figure 1H, cell lines over-expressing either FLAG-CUL3^{WT} or FLAG-CUL3$^{\Delta 403\text{-}459}$ were immunoprecipatated with M2 (anti-FLAG) resin. Input: Cellular extract IP: Immunoprecipated protein sample. Unbound: Protein remaining in extract following IP.
C. Coomassie SDS PAGE of reactions immunoblotted for and shown in Figure 2A.
D. Entire coomassie stained gel of Figure 2B.
E. Full commassie SDS PAGE of reactions immunoblotted for and shown in Figure 3A.

Appendix Figure S3.

The knockout strategy of exon 9 of endogenous Cullin3. The endogenous allele is represented and the target allele with the puromycin cassette (PuroR) removed by Flp recombinase. The black rectangles represent exons and the flippase-recognition target (FRT) sites are indicated.

Appendix Figure S4.

A. Illustrative side-by-side size comparisons of male and female CUL3$^{WT/\Delta 403\text{-}459}$ and CUL3^{WT} littermates. Scale bar = 2cm.
B. CUL3$^{WT/\Delta 403\text{-}459}$ exhibit features of growth retardation when compared with CUL3WT mice. The CUL3WT/Δ403-459 have lower body weight (male: * P=0.0128 // female: *** P=3.3x10^{-5}) and length [measured nose-to-anus] (male: *** P=0.0002 // female: *** P=0.0009), although with no changes in proportionality as measured by tail-to-body ratio (male: P=0.1654 // female P=0.5817). Data are mean ± SEM (male n-values: CUL3^{WT} = 8, CUL3$^{WT/\Delta 403\text{-}459}$ = 11 for body length; CUL3^{WT} = 8, CUL3$^{WT/\Delta 403\text{-}459}$ = 6 for body weight // female n-values: CUL3^{WT} = 16, CUL3$^{WT/\Delta 403\text{-}459}$ = 21 for body length; CUL3^{WT} = 14, CUL3$^{WT/\Delta 403\text{-}459}$ = 12 for body weight). Two-tail unpaired student t-test; data are mean±SEM.

Appendix Figure S5.

A and B. Western blots showing expression of KLHL3 (**A**) or, CUL3 (**B**) in the human thoracic aorta. No obvious sex or age differences were observed. Human kidney were used as positive controls.
C. Western blot of HEK-293 cell lysates over expressing KLHL2-GFP or KLHL3-FLAG. The anti-KLHL3 antibody shows an intense band at the predicted molecular weight of FLAG modified KLHL3, confirming its ability to detect KLHL3.
D. Dual channel multiplex western blot of HEK-293 cell lysates over expressing KLHL2-GFP showing a band at the predicted molecular weight for GFP modified KLHL2 with an anti-GFP antibody (red). The anti-KLHL3 antibody (green) detects a non-specific higher weight band that does not overlap with KLHL2-GFP, therefore confirming specificity for KLHL3 with no cross-reactivity for KLHL2.

Appendix Figure S6.

A. $CUL3^{WT/\Delta 403\text{-}459}$ thoracic aorta have increased phosphorylation of MYPT1 isoforms. Ratiometric expression of quantified MYPT1 phospho-T696 isoforms (normalized against β-actin) were calculated for $CUL3^{WT/\Delta 403\text{-}459}$ vs $CUL3^{WT}$ on each western blot. The mean of the ratios and bounds of the 95% confidence interval are >1, confirming significantly increased phosphorylation (where ratio = 1 represents no change in phosphorylation). Results are from three separate blots containing independent biological replicates of aortic lysates from both genotypes (total n-values across three blots: $CUL3^{WT}$ = 19 / $CUL3^{WT/\Delta 403\text{-}459}$ = 21). Statistical significance was determined by the ratio t-test (see methods for more information); * P = 0.02.
B. A representative western blot of thoracic aorta MYPT1 phospho-Thr696 isoforms and β-actin expression from $CUL3^{WT/\Delta 403\text{-}459}$ and $CUL3^{WT}$ mice run on the same gel.

Appendix Table S1.

The full table of P-values for Fig EV3.

Appendix Figure S1.

A.

```
CUL3 403  LTEQEVETILDKAMVLFRFMQEKDVFERYYKQHLARRLLTNKSVSDDSEKNMISKLK  459
CUL1 437  PEEAELEDTLNQVMVVFKYIEDKDVFQKFYAKMLAKRLVHQNSASDDAEASMISKLK  493
```

B.

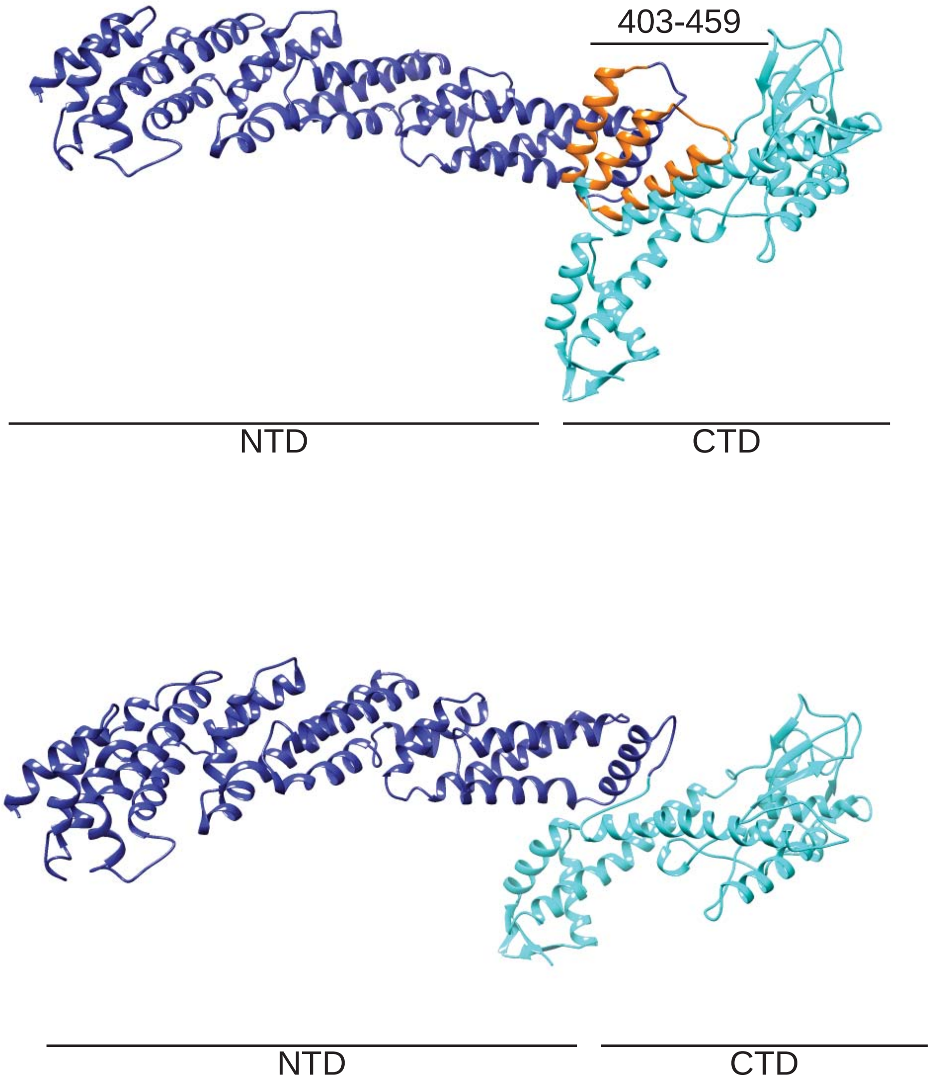

Appendix Figure S2

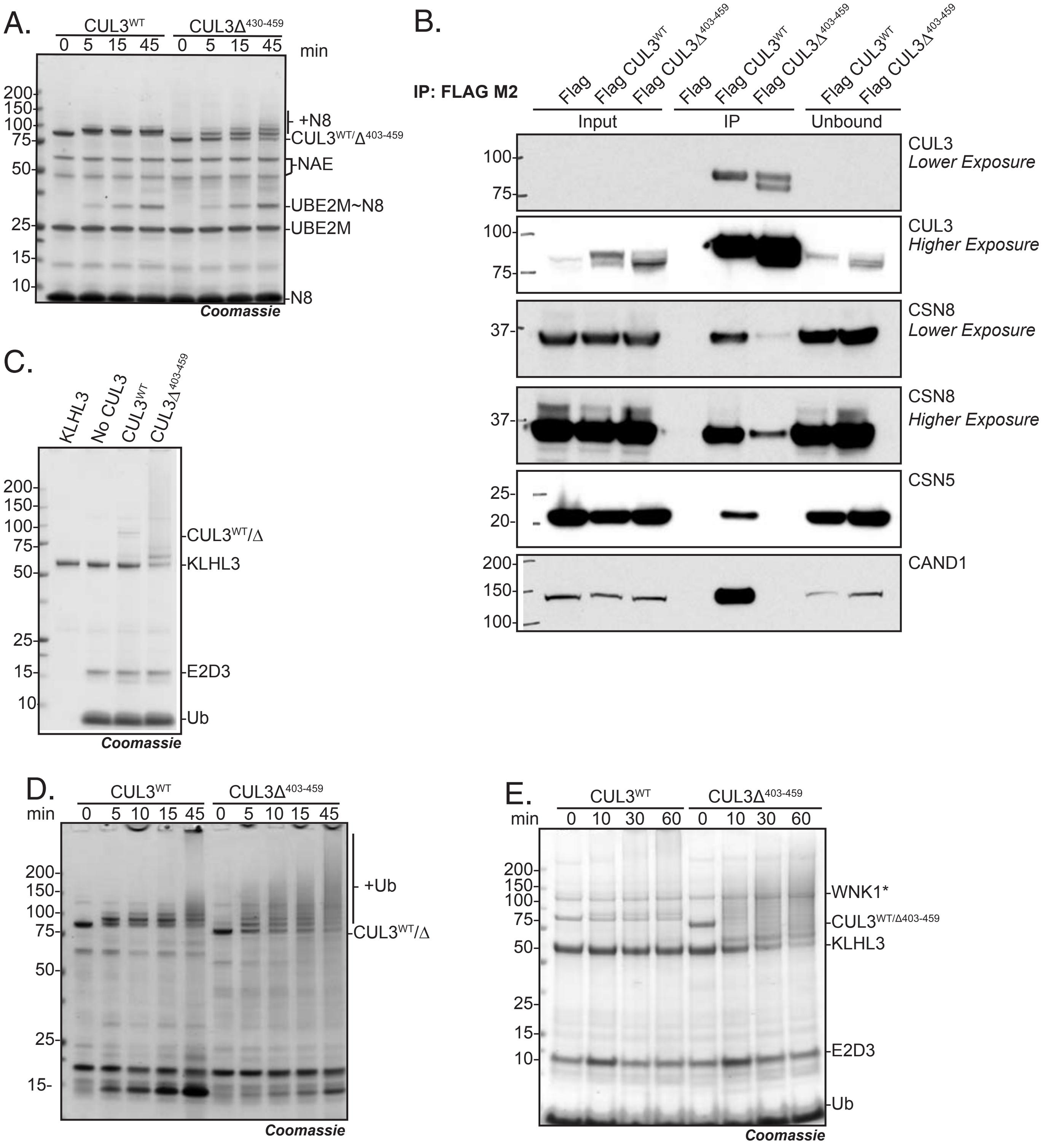

Appendix Figure S3

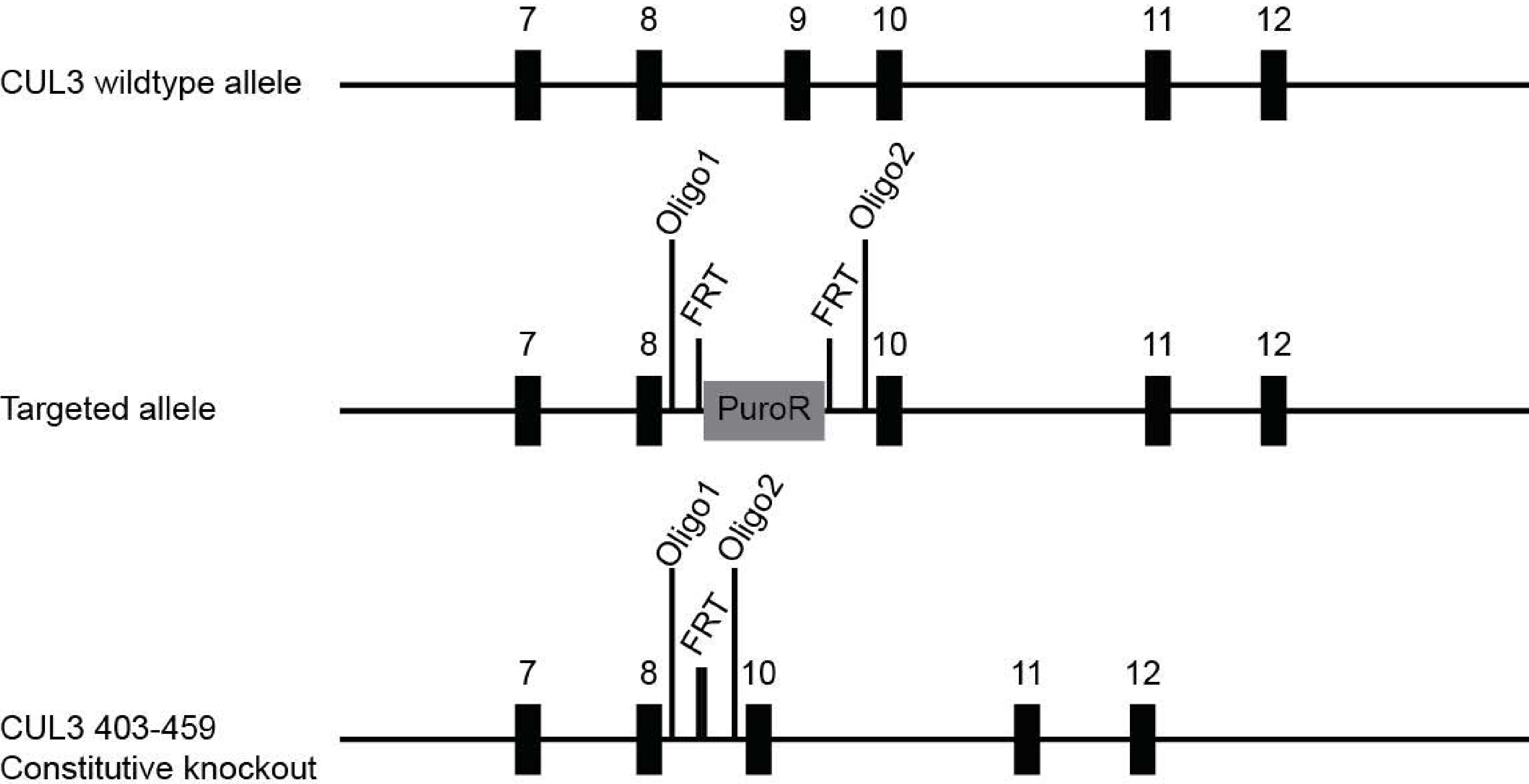

Appendix Figure S4

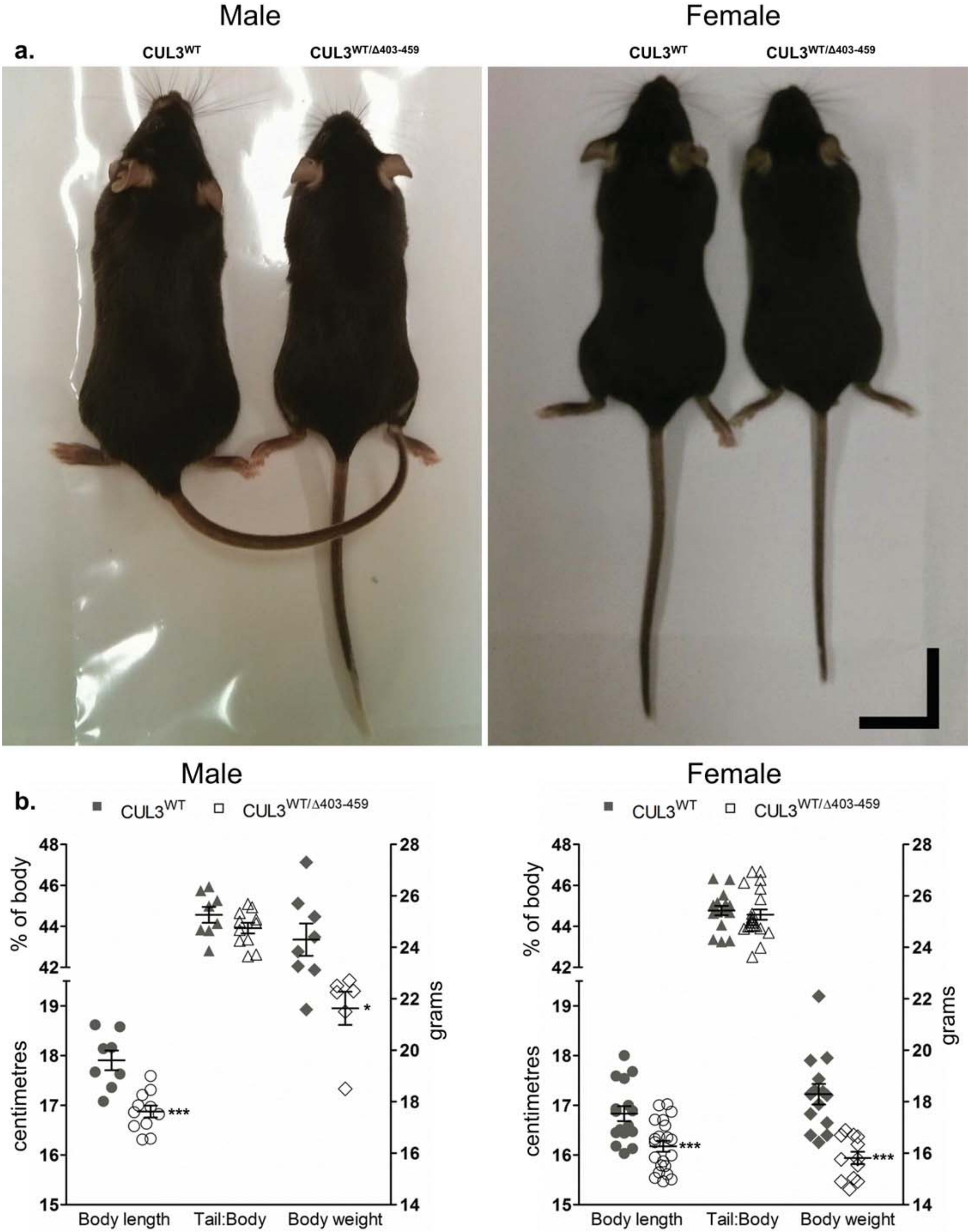

Appendix Figure S5

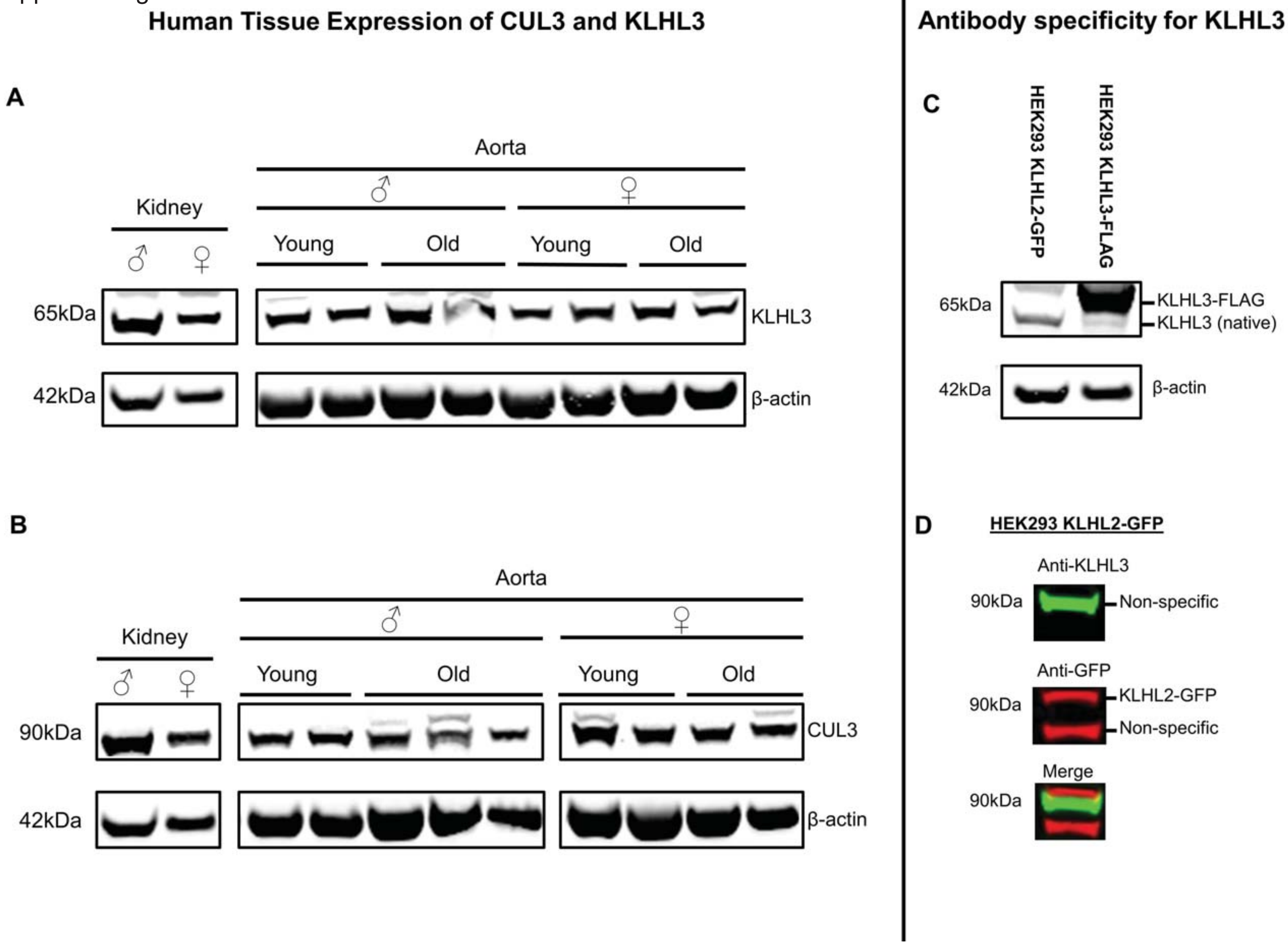

Appendix Figure S6

A.

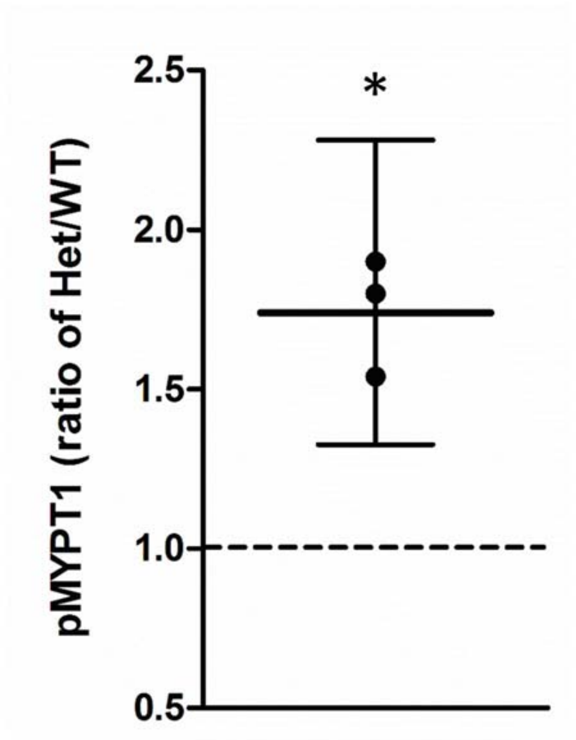

B.

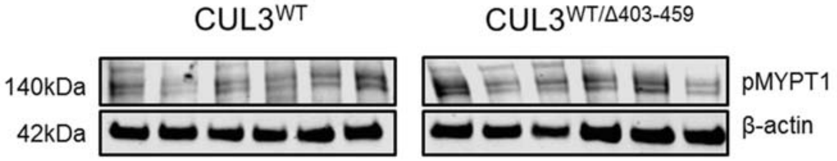

Appendix Table S1

P-values for Fig EV3

Plasma	Cr	K	Mg	Na	Ca	P
NNa $CUL3^{WT}$ vs $CUL3^{WT/Δ403-459}$	0.4326	$4.1x10^{-7}$	0.0110	0.4459	0.0015	0.0129
LNa $CUL3^{WT}$ vs $CUL3^{WT/Δ403-459}$	0.0550	0.0078	0.3749	0.8120	0.8195	0.9470
$CUL3^{WT/Δ403-459}$ NNa vs. LNa	0.9714	0.0004	0.0755	0.0194	0.4859	0.9757
$CUL3^{WT}$ NNa vs. LNa	0.0072	0.6643	0.4043	0.0478	0.0083	0.0493
**Urine**	**Cr**	**K**	**Mg**	**Na**	**Ca**	**P**
NNa $CUL3^{WT}$ vs $CUL3^{WT/Δ403-459}$	0.0424	0.3852	0.2191	0.8236	0.0633	0.4370
LNa $CUL3^{WT}$ vs $CUL3^{WT/Δ403-459}$	0.3864	0.4400	0.4714	0.8700	0.5574	0.2602
$CUL3^{WT/Δ403-459}$ NNa vs. LNa	0.5435	0.4127	0.5515	0.0001	0.0671	0.0031
$CUL3^{WT}$ NNa vs. LNa	0.2503	0.6864	0.0126	$2.5x10^{-6}$	0.1545	0.0395
**Blood**	**Urea**	**Glucose**	**Hct**	**Hb**	**Total CO_2**	**Anion Gap**
$CUL3^{WT}$ vs $CUL3^{WT/Δ403-459}$	0.8914	0.8757	0.8757	0.9045	$3.7x10^{-5}$	0.1022
