# Supplementary material for: Characterisation of the Cullin-3 mutation that causes a severe form of familial hypertension and hyperkalaemia
Source: EMBO Mol Med. 2015 Aug 18;7(10):1285–306. doi: 10.15252/emmm.201505444 (PMC4604684; doi:10.15252/emmm.201505444)
Supplement: Supplementary file 2 [file emmm0007-1285-sd2.pdf]

Expanded View Figures

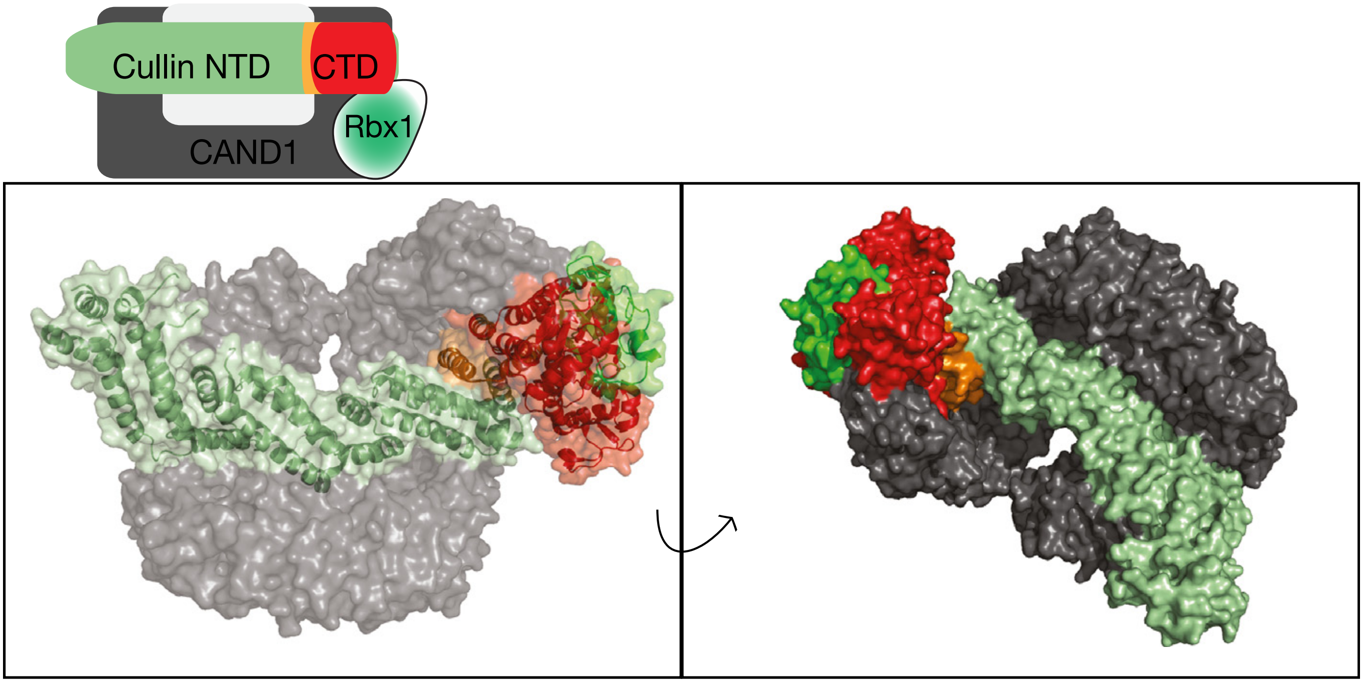

Figure EV1. Structural context of the area deleted by the disease-causing mutation in CUL3.
Top: Schematic representation of the interactions between CRL and CAND1. Bottom: Representation of CAND1 in complex with CUL1-RBX1, PDB 1u6g (Goldenberg *et al*, 2004). Coloured as shown in the schematic representation. Residues 437–493 of CUL1 (equivalent to 403–456 CUL3) are coloured orange.

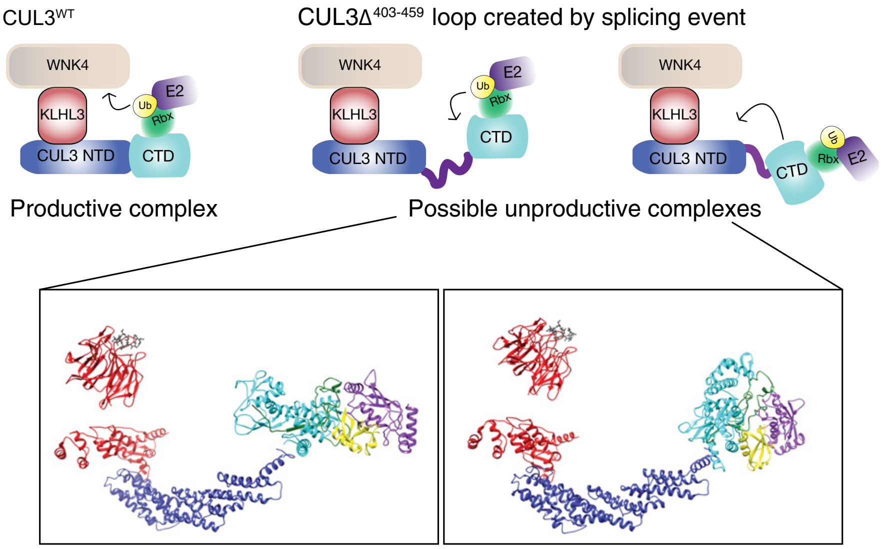

Figure EV2. Schematic representation of the possible structural effects of the mutation.
The top schematics represent a functional CUL3^{WT} complex and show two contrasting positionings for an active CUL3 $^{\Delta 403–456}$ that is unable to modify the WNK kinases. The lower structural representations correspond to the schematics above. These were generated in Chimera, and known structures were utilised as docking references to enable the possible orientations to be explored. KLHL3-KLECH domain (red) bound to a WNK peptide (grey) PDB: 4CH9. KLHL3-BTB domain (red) bound to CUL3-N-terminal domain (blue), CUL3-C-terminal domain (cyan) (based on CUL1 CTD) PDB: 1LDK. RBX1 (green), UBE2D (purple) and ubiquitin (yellow) were docked based on the complex RNF4-E2-UB structure PBD: 4AP4.

© 2015 The Authors

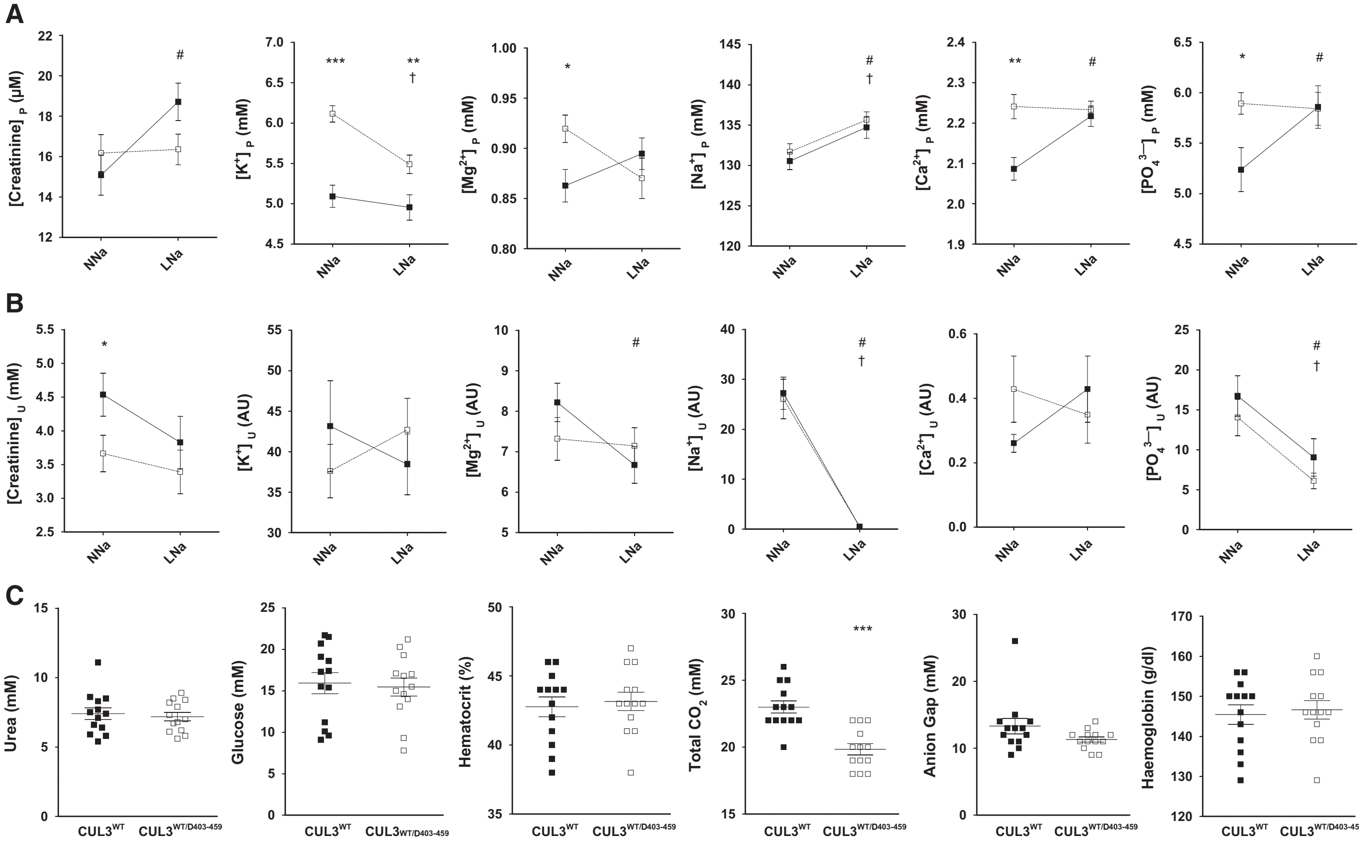

Figure EV3. Plasma and urine electrolyte response to normal and low sodium diets.

A–C The upper (A) and middle panels (B) show plasma (P) and urinary (U) electrolytes, respectively, for CUL3^{WT} mice (■) versus CUL3$^{WT/\Delta403–459}$ (□) on either a normal-salt (NNa) (0.3%) or low-salt (LNa) (0.03%) diet measured by ICP-OES analysis. The urinary values (AU) are individually ratioed to the urinary concentration of creatinine ($[\text{analyte}]_U/[\text{creatine}]_U$). The lower panel (C) shows blood biochemistries not reported in Figure 5D taken after a minimum 4-h fast by iSTAT analysis. The differences between genotypes that are significantly different are shown as *, the differences between NNa and LNa for CUL3^{WT} that are significantly different are shown as #, and the differences between NNa and LNa for CUL3$^{WT/\Delta403–459}$ that are significantly different are shown as † (*n*-values as follows: CUL3^{WT}: plasma NNa = 16; plasma LNa = 17; urine NNa = 18; urine LNa = 16; blood biochemistry = 13; CUL3$^{WT/\Delta403–459}$: plasma NNa = 23; plasma LNa = 23; urine NNa = 21; urine LNa = 19; blood biochemistry = 13). Two-tailed unpaired Student's *t*-test for comparisons between genotypes and two-tailed paired Student's *t*-test for comparisons between diets within the same genotype; data are mean ± SEM. A full table of *P*-values for this figure is shown in Appendix Table S1.

Source data are available online for this figure.

 © 2015 The Authors

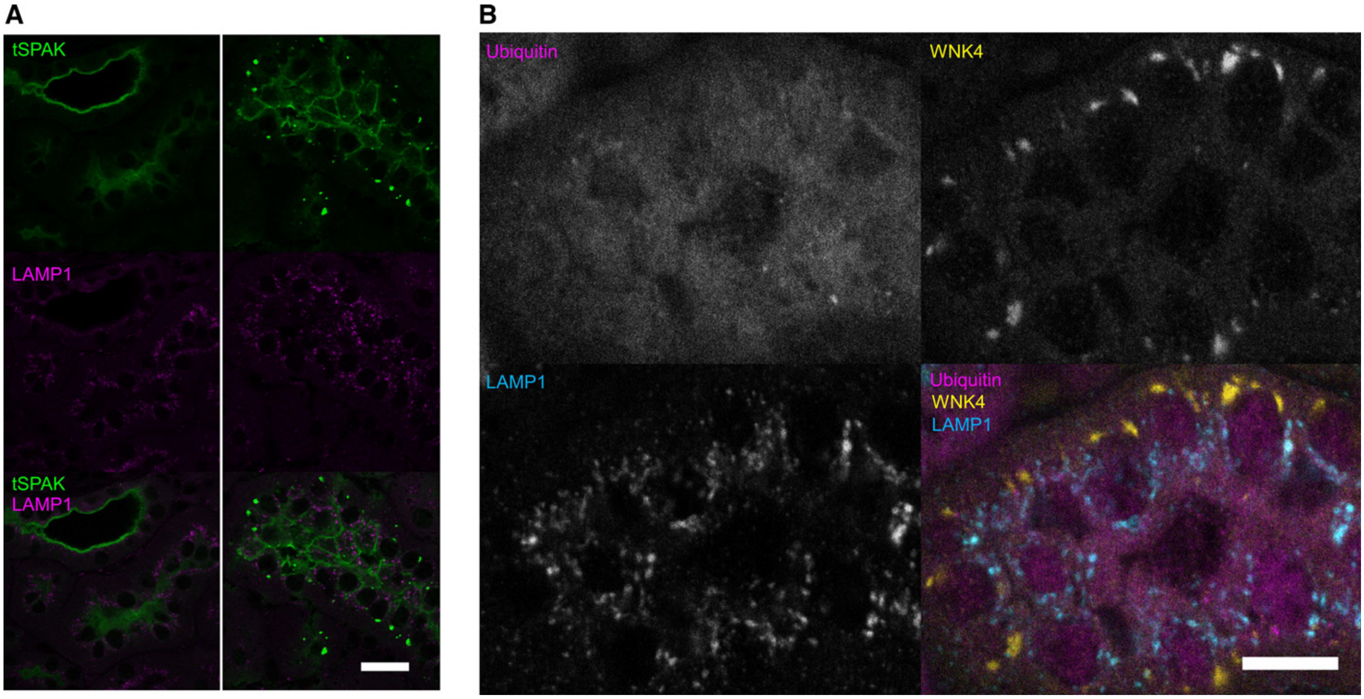

Figure EV4. WNK4 and SPAK puncta do not colocalise with LAMP1 or form ubiquitin-containing aggregates.
Representative pseudocoloured confocal single focal plane images of immunofluorescently stained kidney sections (n = 4 per genotype). WNK4 and SPAK form discrete puncta in the distal convoluted tubule of CUL3$^{WT/\Delta403-459}$ mice. These puncta do not colocalise with lysosomes (LAMP1) or form ubiquitylated aggregates. The puncta have a predominantly basolateral preference with several large juxta-nuclear puncta per cell.

A Immunolocalisation of total SPAK protein (tSPAK) puncta and LAMP1 in the distal convoluted tubule of CUL3$^{WT/\Delta403-459}$ versus CUL3^{WT} mice at a 4-h fasting baseline. Scale bar, 20 μm.

B Immunolocalisation of total WNK4 puncta, ubiquitin and LAMP1 in the distal convoluted tubule of CUL3$^{WT/\Delta403-459}$ versus CUL3^{WT} mice at a 4-h fasting baseline. Scale bar, 10 μm.

© 2015 The Authors
