# Supplementary material for: Characterisation of the Cullin-3 mutation that causes a severe form of familial hypertension and hyperkalaemia
Source: EMBO Mol Med. 2015 Aug 18;7(10):1285–306. doi: 10.15252/emmm.201505444 (PMC4604684; doi:10.15252/emmm.201505444)
Supplement: Supplementary file 3 [file emmm0007-1285-sd3.pdf]

EMBO Molecular Medicine

Characterisation of the Cullin-3 mutation that causes a severe form of familial hypertension and hyperkalaemia

Frances-Rose Schumacher, Keith Siew, Jinwei Zhang, Clare Johnson, Nicola Wood, Sarah E. Cleary, Raya S. Al Maskari, James T. Ferryman, Iris Hardege, Yasmin, Nichola L. Figg, Radoslav Enchev, Axel Knebel, Kevin M. O'Shaughnessy and Thimo Kurz

Corresponding authors: Frances-Rose Schumacher, University of Dundee; Kevin O'Shaughnessy, University of Cambridge; Thimo Kurz, University of Dundee

Review timeline:

Submission date:	27 March 2015
Editorial Decision:	12 May 2015
Manuscript transferred:	18 May 2015
Editorial Decision:	22 May 2015
Revision received:	08 June 2015
Editorial Decision:	06 July 2015
Revision received:	17 July 2015
Accepted:	21 July 2015

Transaction Report:

(Note: This manuscript was originally submitted to The EMBO Journal, and send for peer review. Following the editorial decision, it was transferred to EMBO Molecular Medicine.

With the exception of the correction of typographical or spelling errors that could be a source of ambiguity, letters and reports are not edited. The original formatting of letters and referee reports may not be reflected in this compilation.)

Editors: Hartmut Vodermaier, Céline Carret

1st Editorial Decision 12 May 2015

Thank you for the submission of your manuscript entitled "Cullin-3 mutation (CUL3Δ403-459) causes a severe subtype of familial hypertension and hyperkalaemia (FHHt type PHA2E): characterisation of its molecular basis and a novel vascular phenotype" to The EMBO Journal. Please note that Hartmut Vodermeier is now out of the office attending family issues and will not be able to handle your manuscript. We have now received the full set of reports from the referees, which are copied below, and I regret to say that the outcome is not a positive one.

As you will see from their reports, although referee #3 is more positive, both referees #1 and #2 agree on the unsuitability of your work for The EMBO Journal. In essence, without repeating here their arguments that you will find below, they express concerns regarding both the conceptual novelty and the depth of analysis of your work. With these assessments in hand, after extensive discussions within the editorial team, I am afraid that we cannot offer to further consider your study for publication.

I am sorry that I have to disappoint you at this stage. I hope, however, that the referee comments will help you improve your study and I thank you once more for the opportunity to consider your manuscript.

****
REFEREE COMMENTS

Referee #1:

The authors show that the Cullin-3 mutant no longer ubiquitylates WNK kinases. Instead, this deletion mutant auto-ubiquitylates and loses interactions with two regulators, CSN and CAND1. A knock-in mouse model of Cullin-3, missing the 9th exon, fully recapitulates the human Familial Hyperkalaemia and Hypertension (FHHt) phenotype. The mice also display a change in their arterial pulse waveform, which the authors suggest implies an independent vascular contribution to hypertension. The authors suggest that aortic central pressure augmentation together with WNK signalling can explain the severe phenotype in CUL3Δ403-459 patients.

1. The paper is written in a very succinct fashion. There is an introductory paragraph that presumably serves as an abstract, but the manuscript would greatly benefit from an Introductory section. It would also benefit from a Discussion section. The Results section is also very tersely presented, making it very difficult for the reader to evaluate the data without going back and forth to the figure legends and figures where there is some annotation. So, while the data are substantial, they are not presented in a way that we know what the purpose of the experiments are, how the data support a conclusion and how the conclusion is important given the prior state of knowledge of CUL3. While the mechanisms are of interest the fundamental association of CUL3 and ubiquitylation of WNKs and development of FHHt in humans has been well established.

2. In the absence of completely described experimental rationale and results in the Results section, the figure legends at times are inadequately informative. For example, if one looks at figure legend 2G, one can see the modified mobility, but the rationale that for concluding that it is related to the covalent attachment of ubiquitin is not adequately presented.

3. The novelty of this contribution is somewhat limited since it is already known that the CUL3 mutation that the authors are studying is associated with FHHt type PHA2E in man. It has been known that this mutation abrogates Cul3's ability to ubiquitylate targets normally bound by KLHL3 such as the WNK kinases (e.g. reviewed by Anderica-Romero, Cell Signalling 2014).

4. The authors often do not explain the results or draw specific conclusions. As an example they conclude that multiple NEDD8 molecules are attached to the mutant CUL3 and refer to figures 2a and supplemental figure 3a but how they came to the conclusion regarding NEDD8 from the data presented is not well delineated. This characterized multiple other aspects of the paper. So, while the data is substantial, it is not presented in a way that we know what the purpose of the experiments are, how the data support a conclusion and how the conclusion is important given the prior state of knowledge of CUL3.

5. An important conclusion drawn by the authors is that the knock-in mouse model of CUL3, WT/Δ403-459, has an independent vascular contribution to hypertension unreported in other FHHt models. The systolic waveform is augmented and the diastolic relaxation significantly slowed. The authors interpret this as change consistent with a stiffened vascular wall, and report this as a novel finding representing the previously unrealized vascular phenotype. This is markedly overstatement the data, since the vascular phenotype may all be secondary to hypertension. Hypertension is well known to cause vascular stiffness. There is no reason to propose an independent process to explain the vascular stiffness.

Referee #2:

The Cul3 ubiquitin ligase and its substrate adaptor Klhl3 drive the ubiquitylation and proteasome-dependent degradation Wnk kinases, thereby regulating salt retention in the kidney. This process, identified in part by this PI's laboratory, is inhibited by mutation of Cul3 (deletion of exon 9) or Klhl3, leading to FHHt. It was unclear how mutation of the cullin scaffold contributes to the disease phenotype, as exon 9 does not encode for amino acids predicted to be involved in binding to a BTB-substrate receptor or an E2 enzyme. In this study, the authors show that, indeed, deletion of exon 9

in Cul3 neither prevents Klhl3- or E2-binding nor does it affect ubiquitin discharge from the E2. However, it allows for increased autoubiquitylation of Cul3 and Klhl3, and it inhibits ubiquitylation of Wnks, an observation that the authors interpret as evidence for increased and deleterious conformational flexibility of the Cul3 backbone. Moreover, deletion of exon 9 interferes with binding of Cul3 to Cand1 and the CSN signalosome, two regulators of cullin-dependent RING ligases that have mostly been studied in the context of the SCF/CUL1. The authors then proceed to generate a knock-in mouse model of Cul3-exon 9 deletes, and they show that this mouse model results in accumulation of Wnks, but not Klhl3. Furthermore, the knockin-mice recapitulate phenotypes seen in FHHt, further underscoring the importance of Wnk stabilization for pathology, and illustrating a potential role of Cul3 in regulating arterial function.

The experiments in this manuscript have been executed carefully, and the study reports many interesting findings. However, the two parts of the study, i.e. the mechanistic part investigating the consequences of exon 9-mutation and the mouse in vivo part, are not well connected and don't feed of each other. They are pretty much two independent stories. Furthermore, this reviewer has reservations about the proposed mechanism of Cul3-inactivation (i.e. increased structural flexibility); indeed, the experiments shown here would be consistent with such a mechanism (as well as with several others), but don't test for it directly.

Specific issues:

1. The authors suggest that the Cul3-deletion mutant displays higher conformational flexibility, which promotes Cul3 neddylation at non-physiological sites and Cul3 and Klhl3 autoubiquitylation and prevents substrate modification. I don't find this hypothesis all too convincing. While increased flexibility provides one possible explanation, an alternative (and in my opinion more likely) explanation could be in the architecture of the mutant E3 complex: the active site environment of the Nedd8- or ubiquitin-E2 could be altered in a way that more lysine residues are presented on the Cul3 or Klhl3 (i.e. at a surface away from the substrate binding interface), while the substrate might not be facing the E2 in a productive manner. If conformational flexibility would be the sole reason for the altered catalytic activity, I would have expected increased Cul3- or Klhl3 modification, while at the same time more residues on the substrate might be modified, which, clearly, is not the case. Thus, the authors would need to provide more evidence for "flexibility": can they use mass spectrometry coupled to crosslinking to show that there is increased conformational flexibility between N- and C-terminal domains in mutant Cul3? (maybe an NMR chemical shift experiment would be more straightforward). Can they show that more sites are ubiquitylated on mutant Cul3 or Klhl3 bound to mutant - are they all over the protein or do they cluster in a particular surface? As this model of increased structural flexibility is the major mechanistic contribution of this paper, more work is required for solidify the authors' conclusions.

2. A major limitation of the first part of the study is that it is descriptive, but does not directly test for the proposed model. It would greatly strengthen the manuscript, if the authors could restrict the conformational flexibility of the exon 9 delete (for example, but limiting the length of the loop connecting the N- and C-terminal halves of Cul3) and show that this would restore function towards Wnk kinases.

3. A more minor, but related issue: the experiment in Fig. 2h (methyl-ubiquitin) does not show a dramatic increase in the number of modified lysine residues (it's still mostly one residue in mutant Cul3, with a minor population of CUl3 molecules with two or three ubiquitins). Instead, it shows an acceleration of the modification reaction and an increase in its efficiency. To be consistent with the "increased flexibility" model, the authors would need to show that even in the mostly mono-ubiquitylated mutant Cul3, multiple Lys residues would be target (the reaction would just stop after a single modification). To me, however, it looks much more like an increase in modification rate and efficiency, potentially due to better exposure of lysine residues in Cul3.

Referee #3:

In this paper, the authors investigated the mechanism underlying the pathogenesis of a mutant form of CUL3, Δ403-459 resulting from exon 9 deletion, that has been linked to the development of a severe subtype of familial hypertension and hyperkalaemia (FHHt type PHA2E). Two major

findings are reported in this paper. First, the authors demonstrated that the deletion did not affect the binding of CUL3 with its catalytic subunit, RBX1/ROC1, or BTB-adaptor protein KLHL3 that functions to recruits substrate to CRL3. Instead, the deletion results in hyper-neddylation, leading to the disruption of the binding of CAND1, an exchange factor that regulate the cycle of CRL complex. Second, the authors created knock-in mouse model for Cul3(Δ403-459). Using this model, they went on to demonstrate the functional deficit in promoting the degradation of a key substrate of CUL3-KLHL3, WNK4. Pathological analysis of the Cul3(Δ403-459) mice demonstrate that they develop defects resemble to that seen in FHHt human individuals.

Overall, this is a very nice study that combines the biochemical and genetic analyses of a disease-linked mutant protein. Both biochemical finding and phenotypical analysis are solid. The mechanism of disrupting neddylation by the deletion is novel and the Cul3(Δ403-459) mutant represents a useful mouse model. The paper fits with the EMBO J nicely. I only have two relative minor comments.

Fig. 1a. The identity of the band migrating below 17 kDa need to be confirmed by western to be Rbx1.

Fig. 3a. The Δ403-459 runs apparently slower than the wild-type CUL3 (e.g. Fig. 2a). Why are the wild-type and Δ403-459 CUL3 run as one band in the top panel, instead of two as seen in the bottom panel? This is important as it relates to two alternative models the authors proposed; dominant-negative vs haplo-insufficiency.

Fig 1 and Fig 2. The findings shown in Fig. 1, while necessary, are all 'negative' and thin, while the Fig. 2 is very crowded. It might be better to move Fig. 2a - 2f to Fig. 1.

Manuscript transferred 18 May 2015

We have recently had the above manuscript under evaluation at EMBO Journal. I understand that prior to our submission, Hartmut Vodermaier had already talked to you to gauge if you would potentially be interested in our story if EMBO J were unable to accept it for publication. We have now received the reviews (pasted below), and unfortunately, EMBO J decided to reject the manuscript. We believe that the reviews are not at all bad and suspect that the decision could have gone either way. Reviewer 1 was very upset about the format of the manuscript, as it doesn't adhere to EMBO J style, but this is due to the fact that we directly transferred the paper from Nature Medicine, which as a short format journal has very different requirements. Furthermore, we believe that we can address all concerns raised by the reviewers in a very short time frame by either experimentation or rewriting and hope that you would consider the manuscript for publication at EMBO Mol Med.

Importantly while we agree in part with reviewer 1 (point 3) that it is known that the CUL3 mutation is associated with PHA2E, and we previously demonstrated that WT CUL3 is able to directly ubiquitylate the WNK kinases, we do no think that this affects the novelty of our findings, as we describe for the first time a molecular mechanism that leads to disease, which we would like to explain more fully in our rebuttal as well as in our expanded formatted manuscript. Briefly, we would like to emphasise that our study describes the molecular mechanism that leads to WNK stabilisation and thus PHA2E through CUL3 mutation. Importantly, we describe the first knock-in mouse of a PHA2E CUL3 mutation, which entirely recapitulates the human phenotype. Our combination of a careful in vitro approach together with investigations of our model mouse that phenocopies PHA2E, has provided us with a unique opportunity to clarify what events lead to PHA2E when the CUL3 protein is mutated. Previous papers have utilised indirect cellular systems and mouse kidney-specific CUL3 knockout mouse, rather than a knock-in of the disease mutation, which importantly did NOT recapitulate the human phenotype, but led to massive kidney fibrosis.

Furthermore, we agree with reviewer 2 that the vascular stiffness we are reporting based on the changes in the pulse pressure wave form and the diastolic relaxation could be explained as an indirect affect of raised blood pressure and we are happy to discuss this possibility and a revised manuscript. At the same time we are currently investigating the possibility that the mutation affects

the levels of pMYPT1. Levels of this key phospho-protein (as a biomarker of contraction) could be increased, which would not be expected if the vasculature was simply stiffened as a response to raised BP, as there is good precedent in the literature for this mechanism in a monogenic syndrome caused by mutations in the PPARgamma transcription factor (Pelham et al 2013). These mutations cause hypertension and insulin resistance and the mechanism here is again thought to involve vascular contraction with increase pMYPT1 in the vessel wall. So the discovery of a similar mechanism in FHHt is both novel and exciting and may explain why the vascular phenotype of the CUL3 mutations are more severe (both in terms of age of onset and effect size) than the other mutations that cause the FHHt phenotype.

As the key findings of our paper is ultimately the molecular mechanism underlying the loss of function of CUL3, which reviewer 2 also recognizes as substantial, the vascular contribution, while attractive, is not as critical for the message of the paper.

We are very happy that reviewer 3 considered our work interesting and important and are glad that he/she would have supported publication at EMBO J and are confident that he/she would also support publication at EMBO Molecular Medicine.

Please let us know if you are interested in our work and we'll be happy to transfer the manuscript and start the process.

Reference:
Pelham CJ, Ketsawatsomkron P, Groh S, Grobe JL, de Lange WJ, Ibeawuchi SR, Keen HL, Weatherford ET, Faraci FM, and Sigmund CD. Cullin-3 regulates vascular smooth muscle function and arterial blood pressure via PPARgamma and RhoA/Rho-kinase. Cell Metab 16: 462-472, 2012.

2nd Editorial Decision 22 May 2015

Thank you for providing the rebuttal letter and suggesting to transfer the manuscript to us. I have now received the advice I thought from one of our editorial board adviser.

Our advisor agrees that your article would be a nice contribution to EMBO Molecular Medicine should you revise it according to your letter (form and content-please see below for formatting recommendations), and maybe providing additional translational insights as suggested by this expert who said: "nice manuscript, combining a disease model for FHHt with a very careful and extensive analysis on the mechanisms. [...] Since EMM always emphasises translational aspects, perhaps the authors could attempt to optimize the therapy of hypertension in FHHt patients, using their mouse model, for example by specifically antagonizing vascular constriction (if this assumption is correct). This would certainly be a good selling point to [...] EMM".

We agree that this would make the paper even a better fit for our journal and as such, I really hope that you will be able to revise your article along these lines.

We normally allow 3 months of revision, but should you need more, please do let me know.

I look forward to seeing a revised form of your manuscript as soon as possible.

1st Revision - authors' response 08 June 2015

Point-by-Point response

<u>Response to EMM Advisor:</u>

"nice manuscript, combining a disease model for FHHt with a very careful and extensive analysis on the mechanisms. [...] Since EMM always emphasises translational aspects, perhaps the authors

could attempt to optimize the therapy of hypertension in FHHt patients, using their mouse model, for example by specifically antagonizing vascular constriction (if this assumption is correct). This would certainly be a good selling point to [...] EMM".

We greatly appreciate the kind comments and recognition of our work. We also agree that our results have potential consequences for the treatment of FHHt patients, especially of the PHA2E type. We have now included a discussion of potential translational aspects of our work in the "Discussion section" of the revised manuscript emphasizing how our results may, in the future, lead to an optimized treatment of patients. Extensive additional animal experiments were unfortunately not possible due to the time constraints of the current submission, but we hope to perform these in the future.

Response to EMBO J reviewers:

Referee #1:

General Points:

Referee 1 thought our data was substantial yet was clearly very disappointed about the format of the paper, which we had directly moved as a short format paper to EMBO J. We were assured that it was not necessary to reformat the manuscript prior to review and we were surprised that this fact was not relayed to the reviewers. We believe that most concerns referee 1 raised can be addressed by changing the format to more clearly state the logic behind our experiments. The only major concern of reviewer 1 relates to the fact that we suggest a novel vascular contribution to PHA2E. We have provided additional support for this claim (and new data) as discussed below, and we have discussed in the revised manuscript that although elevated blood pressure can lead to vascular stiffening we propose it is more likely to be a primary change in the vessel wall.

The authors show that the Cullin-3 mutant no longer ubiquitylates WNK kinases. Instead, this deletion mutant auto-ubiquitylates and loses interactions with two regulators, CSN and CAND1. A knock-in mouse model of Cullin-3, missing the 9th exon, fully recapitulates the human Familial Hyperkalaemia and Hypertension (FHHt) phenotype. The mice also display a change in their arterial pulse waveform, which the authors suggest implies an independent vascular contribution to hypertension. The authors suggest that aortic central pressure augmentation together with WNK signalling can explain the severe phenotype in CUL3Δ403-459 patients.

1. The paper is written in a very succinct fashion. There is an introductory paragraph that presumably serves as an abstract, but the manuscript would greatly benefit from an Introductory section. It would also benefit from a Discussion section. The Results section is also very tersely presented, making it very difficult for the reader to evaluate the data without going back and forth to the figure legends and figures where there is some annotation. So, while the data are substantial, they are not presented in a way that we know what the purpose of the experiments are, how the data support a conclusion and how the conclusion is important given the prior state of knowledge of CUL3. While the mechanisms are of interest the fundamental association of CUL3 and ubiquitylation of WNKs and development of FHHt in humans has been well established.

We agree and apologise for this. It is unfortunate the manuscript that was sent for review to EMBO J was left in its original very condensed version (to comply with a tight word limit). We have rectified this and expanded the article in the revised manuscript. Also, while we agree that CUL3 has been linked to the ubiquitylation of WNKs and the development of PHA2E based on mutations identified in FHHT patients and indirectly through the study of the CUL3 adaptor KLHL3, our study is the first to provide a detailed molecular characterization of the defects caused by CUL3 mutation and the first to characterize a mouse model of CUL3 that recapitulates the disease.

2. In the absence of completely described experimental rationale and results in the Results section, the figure legends at times are inadequately informative. For example, if one looks at figure legend 2G, one can see the modified mobility, but the rationale that for concluding that it is related to the covalent attachment of ubiquitin is not adequately presented.

We have addressed this in the revised manuscript so that the thought processes are easier to follow. Specifically in regards to figure 2G, this is an established in vitro assay system containing a set of known and defined components, which we expand and justify in the revised manuscript.

3. The novelty of this contribution is somewhat limited since it is already known that the CUL3 mutation the authors are studying is associated with FHHt type PHA2E in man. It has been known that this mutation abrogates Cul3's ability to ubiquitylate targets normally bound by KLHL3 such as the WNK kinases (e.g. reviewed by Anderica-Romero, Cell Signalling 2014).

We would argue with the statement that it has been established that the mutation of CUL3 abrogates CUL3's ability to ubiquitylate substrates. We agree, it has been hypothesized before and the identity of the mutation itself was reported in *Nature* in 2012. However, the review paper the referee refers to suggest that CUL3 mutations may prevent ubiquitylation of substrate proteins, and the citations within this review relating to this statement refer to studies of KLHL3 and WNK kinase mutations not CUL3. The KLHL3 mutations are considered in the context of a CUL3-KLHL3 ligase complex, but crucially, the CUL3 mutation itself is not investigated by any of these studies. The idea that the CUL3 mutation abrogates its ability to modify WNK kinases has been proposed but never shown. Indeed, one reason our paper is exciting, is because we carefully examine for the first time how the CUL3 mutation acts in the context of the WNK signalling pathway and PHA2E.

Hence, we are the first to a) show the mechanism by which the CUL3 mutation causes the FHHt (PHA2E) phenotype, and b) provide *in vivo* evidence that it can directly cause an FHHt phenotype. Specifically, our paper provides both a molecular basis for the defective function of the exon-9 deleted CUL3 protein and shows the precise recapitulation of the PHA2E phenotype in a mouse expressing this mutant form of CUL3. We think both are novel findings and represent a major step forward in our understanding of the molecular basis of PHA2E.

4. The authors often do not explain the results or draw specific conclusions. As an example they conclude that multiple NEDD8 molecules are attached to the mutant CUL3 and refer to figures 2a and supplemental figure 3a but how they came to the conclusion regarding NEDD8 from the data presented is not well delineated. This characterized multiple other aspects of the paper. So, while the data is substantial, it is not presented in a way that we know what the purpose of the experiments are, how the data support a conclusion and how the conclusion is important given the prior state of knowledge of CUL3.

We would agree that in parts the descriptions of the results lack sufficient detail to make comprehension easy. Again a problem that the reviewed manuscript was the word-constrained original manuscript. We hope the expansion possible in the revised manuscript is easier to follow. In regards to the examples mentioned, this is an established in vitro assay with defined components, given the assay conditions and the fact Nedd8 modifications are covalent, any shift in CUL3 protein mobility can be attributed to Nedd8 attachment. Given the CUL3 WT protein shows a band shift equivalent to one Nedd8 molecule, while the CUL3-delta assay resulted in band shifts equivalent to one, two, or three Nedd8 molecules we concluded multiple Nedd8 molecules were attached to the CUL3-delta protein within our assay. We have made this clearer in the revised manuscript.

5. An important conclusion drawn by the authors is that the knock-in mouse model of CUL3, WT/Δ403-459, has an independent vascular contribution to hypertension unreported in other FHHt models. The systolic waveform is augmented and the diastolic relaxation significantly slowed. The authors interpret this as change consistent with a stiffened vascular wall, and report this as a novel finding representing the previously unrealized vascular phenotype. This is markedly overstatement the data, since the vascular phenotype may all be secondary to hypertension. Hypertension is well known to cause vascular stiffness. There is no reason to propose an independent process to explain the vascular stiffness.

We agree that the changes to the pressure waveform and diastolic relaxation could be explained as secondary to the raised BP in the CUL3 WT/Δ403-459 mice and mention this possibility in the revised discussion. However, we think the changes may also be due to a primary change in the physiology of the vessel wall based on two additional pieces of data that we have included in this manuscript. Firstly, we have data suggesting the contractile state of the vessel (through myosin light chain phosphorylation) is increased in $CUL3^{WT/\Delta403\text{-}459}$ mice. We show in the attached new appendix

figure 6 that the level of phospho-MYPT1 (phosphorylation inhibits the myosin light chain phosphatase MYPT1 to increase the level of activated/phosphorylated myosin LC itself) is increased 1.7-fold ($P<0.02$) in aortic lysates from CUL3 WT/Δ403-459 mice versus the WT vessels (as measured by LiCor quantitative Western blotting using a ratio t-test to determine significance). This would not be expected to occur as a result of a simple elevation of blood pressure (where it would actually represent a positive feedback effect). There is precedent for this, since mice with PPARγ mutations also have hypertension and activation of the same pathway (cited in the manuscript as ref 18). The second piece of data comes from the *in vivo* vasculature contractility experiment (figure 5). We have tested this by administering increasing doses of two vasoconstrictor drugs (Phenylephrine and Angiotensin-II) to generate *in vivo* dose-response curves. The curves are significantly different between $CUL3^{WT/\Delta 403\text{-}459}$ and $CUL3^{WT}$ mice with the CUL3 WT/Δ403-459 mice showing a significantly increased maximum response. Taken together we now believe that the new data presented in figure 6 and appendix figure 6 supports our hypothesis that the pressure wave form effects may reflect a primary change in the vessel wall (through an increased contractile state) not a secondary response to raised BP. This is discussed in full in the revised discussion.

Referee #2:

The Cul3 ubiquitin ligase and its substrate adaptor Klhl3 drive the ubiquitylation and proteasome-dependent degradation Wnk kinases, thereby regulating salt retention in the kidney. This process, identified in part by this PI's laboratory, is inhibited by mutation of Cul3 (deletion of exon 9) or Klhl3, leading to FHHt. It was unclear how mutation of the cullin scaffold contributes to the disease phenotype, as exon 9 does not encode for amino acids predicted to be involved in binding to a BTB-substrate receptor or an E2 enzyme. In this study, the authors show that, indeed, deletion of exon 9 in Cul3 neither prevents Klhl3- or E2-binding nor does it affect ubiquitin discharge from the E2. However, it allows for increased autoubiquitylation of Cul3 and Klhl3, and it inhibits ubiquitylation of Wnks, an observation that the authors interpret as evidence for increased and deleterious conformational flexibility of the Cul3 backbone. Moreover, deletion of exon 9 interferes with binding of Cul3 to Cand1 and the CSN signalosome, two regulators of cullin-dependent RING ligases that have mostly been studied in the context of the SCF/CUL1. The authors then proceed to generate a knock-in mouse model of Cul3-exon 9 deletes, and they show that this mouse model results in accumulation of Wnks, but not Klhl3. Furthermore, the knockin-mice recapitulate phenotypes seen in FHHt, further underscoring the importance of Wnk stabilization for pathology, and illustrating a potential role of Cul3 in regulating arterial function.

The experiments in this manuscript have been executed carefully, and the study reports many interesting findings. However, the two parts of the study, i.e. the mechanistic part investigating the consequences of exon 9-mutation and the mouse in vivo part, are not well connected and don't feed of each other. They are pretty much two independent stories. Furthermore, this reviewer has reservations about the proposed mechanism of Cul3-inactivation (i.e. increased structural flexibility); indeed, the experiments shown here would be consistent with such a mechanism (as well as with several others), but don't test for it directly.

We are pleased the reviewer acknowledges the experiments have been carefully executed and agrees the findings are interesting. We appreciate that, in parts, the biochemical data and the mouse model data may have seemed well connected, but we are confident that we have now rectified this concern with the new extended manuscript. We also feel that the two aspects of the study do inform each other, especially as we can connect the molecular defects we observe in the test tube with *in vivo* phenotypes we observe in the mouse. By way of example, we detect increased CUL3 Δ403-459 auto-ubiquitylation in vitro, which suggest the protein may "auto-degrade" via the proteasome. Consistent with this in vitro finding, we observe a decreased abundance of $CUL3^{\Delta 403\text{-}459}$ protein in the mouse. We discuss this reviewer's reservations regarding CUL3-flexibility below.

Specific issues:

1. The authors suggest that the Cul3-deletion mutant displays higher conformational flexibility, which promotes Cul3 neddylation at non-pyhsiological sites and Cul3 and Klhl3 autoubiquitylation and prevents substrate modification. I don't find this hypothesis all too convincing. While increased flexibility provides one possible explanation, an alternative (and in my opinion more likely) explanation could be in the architecture of the mutant E3 complex: the active site environment of the

Nedd8- or ubiquitin-E2 could be altered in a way that more lysine residues are presented on the Cul3 or Klhl3 (i.e. at a surface away from the substrate binding interface), while the substrate might not be facing the E2 in a productive manner. If conformational flexibility would be the sole reason for the altered catalytic activity, I would have expected increased Cul3- or Klhl3 modification, while at the same time more residues on the substrate might be modified, which, clearly, is not the case.

Thus, the authors would need to provide more evidence for "flexibility": can they use mass spectrometry coupled to crosslinking to show that there is increased conformational flexibility between N- and C-terminal domains in mutant Cul3? (maybe an NMR chemical shift experiment would be more straightforward). Can they show that more sites are ubiquitylated on mutant Cul3 or Klhl3 bound to mutant - are they all over the protein or do they cluster in a particular surface? As this model of increased structural flexibility is the major mechanistic contribution of this paper, more work is required for solidify the authors' conclusions.

This is a valid point and we appreciate the opportunity to address this concern. To further test whether the mutation increases flexibility, we have now performed mass spectrometry analysis of the ubiquitylation sites on CUL3 as suggested by the reviewer. These experiments were performed with methylated ubiquitin (see point 3 below), as methylated ubiquitin is unable to form t ubiquitin chains, this allows for an easier identification of modification sites on CUL3. The outcome of this experiment has been highly informative and supports our model. As shown in the new Figure 3, auto-ubiquitylation of the wildtype form of CUL3 is restricted to maximally five sites, all of which are in the C-terminal half of the protein. The mutant form of CUL3, however heavily auto-ubiquitylates at sites throughout the length of the protein, which we believe can only be explained by increased structural flexibility of the Cullin scaffold, allowing for more flexibility between the N-terminal and C-terminal domains of the Cullin.

We would also like to highlight the fact that it has already been demonstrated that increasing the flexibility between the N- and C-terminal domains of CUL1 (which is also where the Δ403-459 mutation lies in CUL3) leads to a loss of substrate ubiquitylation (Zheng et al. Nature. 2002 Apr 18;416(6882):703-9). The assumption by the reviewer that increased flexibility must lead to increased substrate ubiquitylation is thus incorrect. In fact, if the work of CUL1 is of any guide, increased flexibility is more likely to lead to a loss of substrate ubiquitylation. We now present our idea as a well-supported hypothesis and discuss our rationale in greater detail.

2. A major limitation of the first part of the study is that it is descriptive, but does not directly test for the proposed model. It would greatly strengthen the manuscript, if the authors could restrict the conformational flexibility of the exon 9 delete (for example, but limiting the length of the loop connecting the N- and C-terminal halves of Cul3) and show that this would restore function towards Wnk kinases.

While we agree that we do not directly test for the increased flexibility model, we believe it is the most likely effect of the mutation, which is now further supported by the mass spectrometry experiment outlined above. The experiment the reviewer suggests here, however, is very difficult indeed. We know that the ridigity and orientation of the cullin backbone is critical for substrate ubiquitylation, but reinstating this architecture by reintroducing “stabilizing” mutations is extremely difficult to achieve. It is impossible to predict which mutation to use, as simply limiting the length of the loop is likely not going to produce the desired outcome, as the overall rigid architecture and relative orientation of the subunits towards each other are important for successful substrate ubiquitylation. As such, without a crystal structure of CUL3$^{\Delta 403\text{-}459}$ we would ultimately have to make a series of assumptions regarding how the mutant CUL3 folds to then make predictions regarding how to shorten the loop between the N- and C-terminal domains to limit flexibility, while maintaining the overall architecture of the complex. Most likely, we would alter the overall structure of the scaffold, which would have a plethora of possible effects, which would make it very difficult, if not impossible, to draw meaningful and useful conclusions from such an experiment.

3. A more minor, but related issue: the experiment in Fig. 2h (methyl-ubiquitin) does not show a dramatic increase in the number of modified lysine residues (it's still mostly one residue in mutant Cul3, with a minor population of CUl3 molecules with two or three ubiquitins). Instead, it shows an acceleration of the modification reaction and an increase in its efficiency. To be consistent with the "increased flexibility" model, the authors would need to show that even in the mostly mono-

ubiquitylated mutant Cul3, multiple Lys residues would be target (the reaction would just stop after a single modification). To me, however, it looks much more like an increase in modification rate and efficiency, potentially due to better exposure of lysine residues in Cul3.

We agree with the reviewer and our experiments in the new Fig. 3 now clearly show that the mono-ubiquitylated form of CUL3 can in fact be mono-ubiquitylated at multiple sites.

Referee #3:

In this paper, the authors investigated the mechanism underlying the pathogenesis of a mutant form of CUL3, Δ403-459 resulting from exon 9 deletion, that has been linked to the development of a severe subtype of familial hypertension and hyperkalaemia (FHHt type PHA2E). Two major findings are reported in this paper. First, the authors demonstrated that the deletion did not affect the binding of CUL3 with its catalytic subunit, RBX1/ROC1, or BTB-adaptor protein KLHL3 that functions to recruits substrate to CRL3. Instead, the deletion results in hyper-neddylation, leading to the disruption of the binding of CAND1, an exchange factor that regulate the cycle of CRL complex. Second, the authors created knock-in mouse model for Cul3(Δ403-459). Using this model, they went on to demonstrate the functional deficit in promoting the degradation of a key substrate of CUL3-KLHL3, WNK4. Pathological analysis of the Cul3(Δ403-459) mice demonstrate that they develop defects resemble to that seen in FHHt human individuals.

Overall, this is a very nice study that combines the biochemical and genetic analyses of a disease-linked mutant protein. Both biochemical finding and phenotypical analysis are solid. The mechanism of disrupting neddylation by the deletion is novel and the Cul3(Δ403-459) mutant represents a useful mouse model. The paper fits with the EMBO J nicely. I only have two relative minor comments.

We thank the referee for his/her very positive comments and his/her affirmation that the findings (mechanism and mouse model) are novel.

Fig. 1a. The identity of the band migrating below 17 kDa need to be confirmed by western to be Rbx1.

This Western Blot has now been performed and is included as Fig. 1A.

Fig. 3a. The Δ403-459 runs apparently slower than the wild-type CUL3 (e.g. Fig. 2a). Why are the wild-type and Δ403-459 CUL3 run as one band in the top panel, instead of two as seen in the bottom panel? This is important as it relates to two alternative models the authors proposed; dominant-negative vs haplo-insufficiency.

We acknowledge that this figure was not clearly explained and have corrected this. The top panel in Fig 3a, which in the new version of the manuscript is now Fig. 4A, is a low exposure of a Western Blot of <u>whole kidney lysate</u> that is also shown in a longer exposure immediately below. From these two blots it is apparent that in whole lysate the slower migrating $CUL3^{WT}$ band is the most prominent one and the $CUL3^{\Delta 403-459}$ band is much more difficult to detect and only visible upon longer exposure. The $CUL3^{\Delta 403-459}$ band, however, is much more easily detectable after immunoprecipitation of CUL3 (Western Blot at the very bottom of Fig. 3a – now Fig. 4a). One explanation for the low abundance of $CUL^{3\Delta 403-459}$ in extract could be that it auto-ubiquitylates and degrades itself, which is consistent with our *in vitro* findings that $CUL3^{\Delta 403-459}$ hyper-ubiquitylates. In the manuscript we also discuss the possibility that $CUL3^{\Delta 403-459}$ may be hypermodified with ubiquitin and/or NEDD8, but not degraded. If so, this may still interfere with its detection be Western Blot, as a single modification with either ubiquitin or NEDD8 would lead to the $CUL3^{\Delta 403-459}$ protein migrating directly on top of the WT protein, and as such could not be distinguished from $CUL3^{WT}$.

To clarify this point, we have now explained figure 4A in more detail in the figure legends.

Fig 1 and Fig 2. The findings shown in Fig. 1, while necessary, are all 'negative' and thin, while the Fig. 2 is very crowded. It might be better to move Fig. 2a - 2f to Fig. 1.

We have now re-arranged Fig. 1 to include the suggested panels.

3rd Editorial Decision 06 July 2015

Thank you for the submission of your revised manuscript to EMBO Molecular Medicine and your patience during the review process. We have now received the enclosed reports from the referees who were asked to re-assess it. As you will see the reviewers are now globally supportive and I am pleased to inform you that we will be able to accept your manuscript pending the following final amendment:

As initially agreed, we would not ask you to address referee #1 concerns experimentally, but please discuss the issue adequately in the main article and in your rebuttal letter.

Please submit your revised manuscript within two weeks. I look forward to seeing a revised form of your manuscript as soon as possible.

2nd Revision - authors' response 17 July 2015

Referee #1

The manuscript has been markedly improved. The expansion has added greatly to the readability. The authors argue that their manuscript is the first to provide a detailed molecular mechanism of the CUL3 mutation defect and the first to characterize a mouse model of CUL3 that recapitulates the disease. Most of my concerns have been addressed in the revised manuscript. There are, however, a few residual issues.

1. The authors continue to argue that the hypertension is not a consequence of sodium retention but rather an independent effect on the vascular smooth muscle cells. Since others (including Boyden et al. Nature 482:98-102, 2012) have found that patients with this mutation respond to thiazides it seems that the burden is on the authors to prove that the hypertension is not associated with sodium retention in their model. Furthermore, if it is then it questions their modeling of the phenotype in humans. At the very least, if the authors want to make this point they should show the lack of a response of the animals BP waveforms to thiazide therapy.

We have actually argued that the CUL3 mutation causes the FHHt phenotype primarily through activation of the WNK/SPAK/NCC pathway as with other FHHt causing mutations in KLHL3 and WNK kinases. However, there may be an additional contribution to BP elevation in the CUL3 pedigree from vascular stiffening. This stiffening could itself be a primary phenomenon and not simply secondary to hypertension based on data we have presented. If the same phenomenon exits in PHA2E patients, it would explain the severity of the BP phenotype in PHA2E. We have reworded the Results (pages 13/14) and Discussion (page 17) sections to hopefully make this clearer.

Referee #2

The authors have responded well to my comments on the version I have seen for EMBO J, and the proteomic analysis of ubiquitylation sites in mutant CUL3 is a nice addition. I have no further issues with this study; it is ready for publication.

We thank the referee for his positive comments.

© EMBO
