# Supplementary material for: Characterisation of the Cullin-3 mutation that causes a severe form of familial hypertension and hyperkalaemia
Source: EMBO Mol Med. 2015 Aug 18;7(10):1285–306. doi: 10.15252/emmm.201505444 (PMC4604684; doi:10.15252/emmm.201505444)

SourceData for Figure 1.

1A

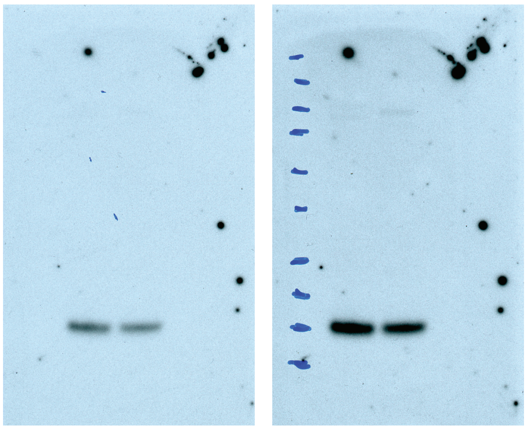

1C

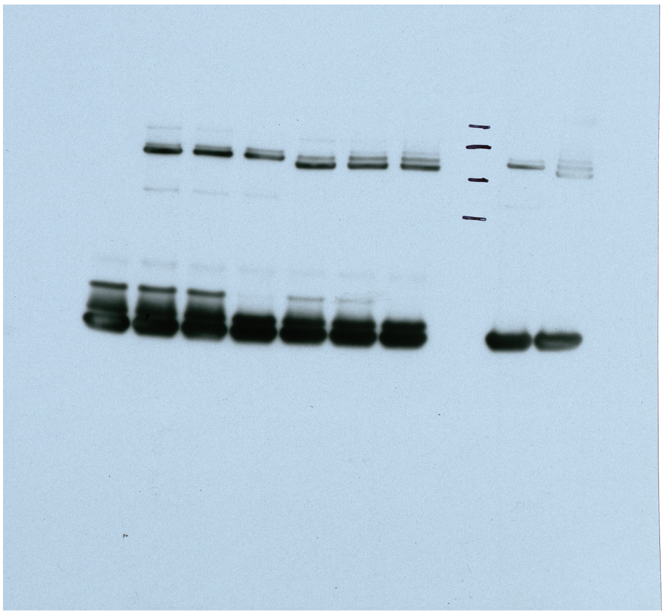

1F

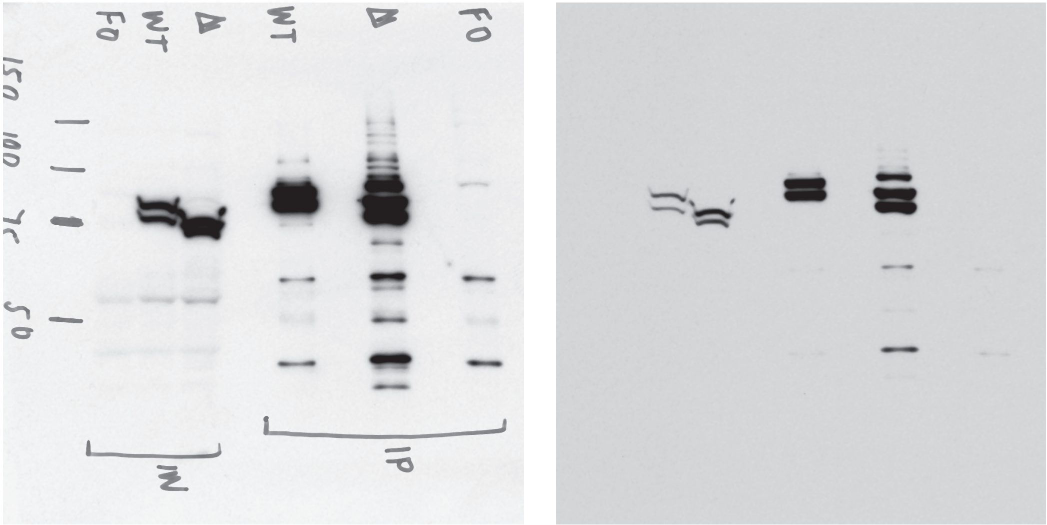

1G

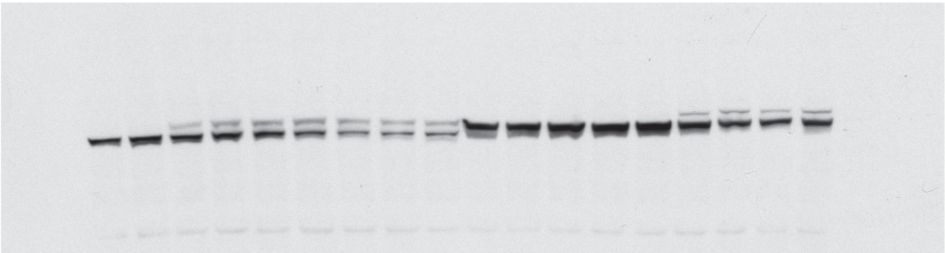

1H IP samples run out on gel, and then membrane cut and probed as shown
All developed on same piece of film and cropped for figure.

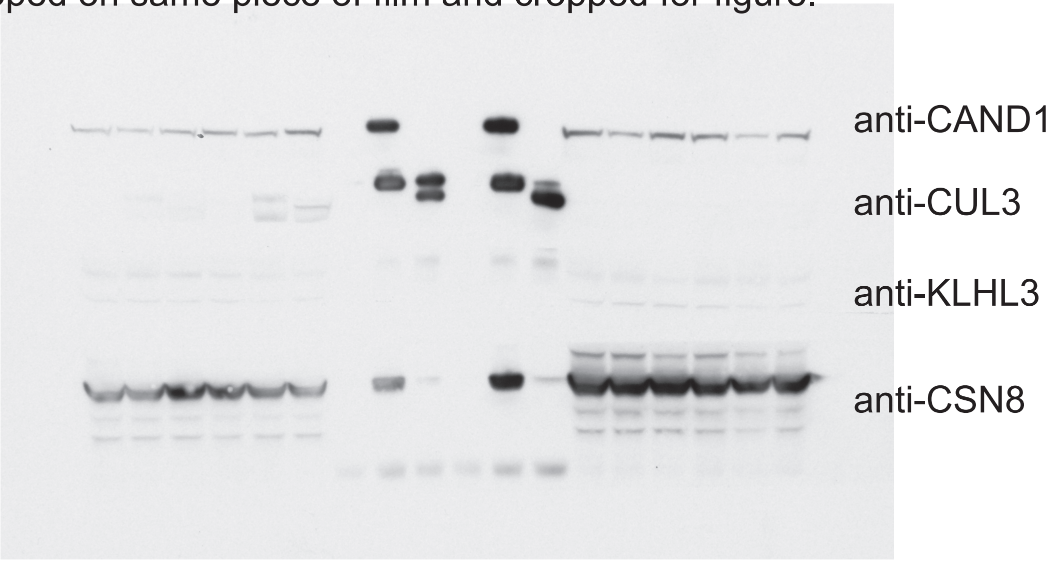

Supplement: Supplementary file 4 [file emmm0007-1285-sd4.pdf]
