# Supplementary figures and images for: Characterisation of the Cullin-3 mutation that causes a severe form of familial hypertension and hyperkalaemia
Source: EMBO Mol Med. 2015 Aug 18;7(10):1285–306. doi: 10.15252/emmm.201505444 (PMC4604684; doi:10.15252/emmm.201505444)

Source Data Fig 2

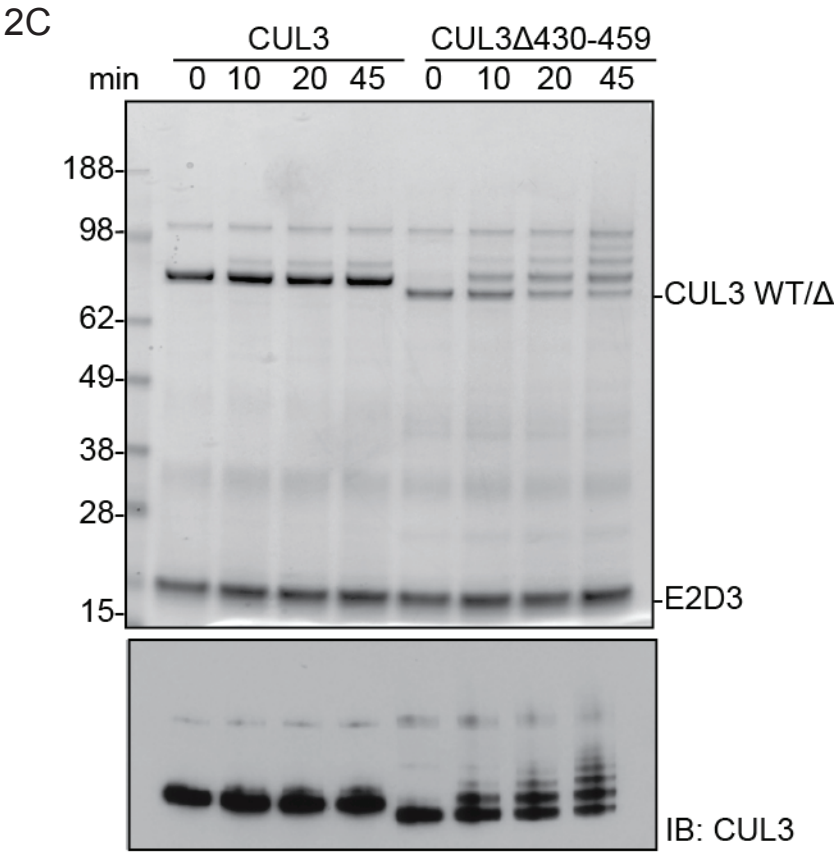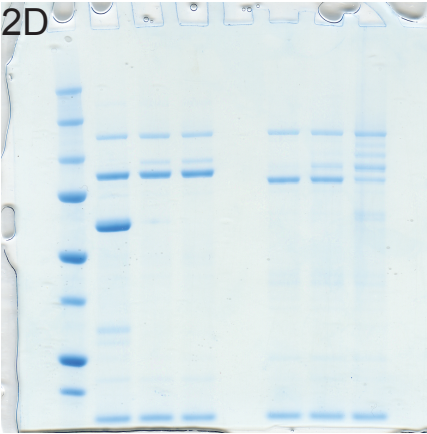

Supplement: Supplementary file 5 [file emmm0007-1285-sd5.pdf]
