# Supplementary material for: Characterisation of the Cullin-3 mutation that causes a severe form of familial hypertension and hyperkalaemia
Source: EMBO Mol Med. 2015 Aug 18;7(10):1285–306. doi: 10.15252/emmm.201505444 (PMC4604684; doi:10.15252/emmm.201505444)
Supplement: Supplementary file 6 [file emmm0007-1285-sd6.pdf]

4C	Aldo level (pg/ml)		
	**Group**	**WT**	**Δ403-459**
		367	162
		387	89
		243	584
		238	1037
		234	813
		305	265
		115	174
		150	659
		395	155
		158	786
		213	510
		374	244
		619	228
		281	452
		166	244
		536	347
		191	1165
		396	369
			328
			227
			851
			695
	**N**	18	22
	**mean**	298	472
	**sem**	33	67

4D	ISTAT Analyte	K	K	Cl
	**Group**	**WT**	**Δ403-459**	**WT**
		4.1	5.4	118
		4.5	4.8	114
		4.3	6.1	116
		4.8	4.7	112
		4.7	5	111
		4.3	6.3	114
		3.7	4.7	118
		4.7	5.6	117
		3.8	5.1	118
		4.2	4.8	117
		4.2	4.1	117
		4.6	5.1	117
		4.1	4.7	116
	**N**	13	13	13
	**mean**	4.31	5.11	115.77
	**sem**	0.10	0.18	0.67

Cl Δ403-460	pH WT	pH Δ403-461	PCO2 WT	PCO2 Δ403-462	HCO3 WT	HCO3 Δ403-463	BE WT	BE Δ403-464
120	7.308	7.278	6.2	5.1	23.3	17.9	-3	-9
118	7.33	7.312	5.61	4.5	22.2	17.1	-4	-9
121	7.293	7.3	5.81	5.7	21.1	21	-5	-5
120	7.228	7.255	7.5	5.65	23.4	18.8	-4	-8
117	7.305	7.248	6.58	6.29	24.6	20.6	-2	-7
121	7.303	7.337	6.07	4.8	22.5	19.3	-4	-7
117	7.286	7.287	5.73	5.64	20.5	20.2	-6	-6
120	7.288	7.296	5.89	4.98	21.2	18.2	-5	-8
118	7.337	7.293	5.52	5.18	22.2	18.8	-4	-8
119	7.284	7.332	5.28	4.37	18.8	17.4	-8	-8
119	7.368	7.338	4.78	5.1	20.6	20.5	-5	-5
120	7.296	7.274	5.68	5.18	20.8	18	-6	-9
122	7.256	7.285	6.43	4.65	21.4	16.6	-6	-10
13	13	13	13	13	13	13	13	13
119.38	7.30	7.30	5.93	5.16	21.74	18.80	-4.77	-7.62
0.45	0.01	0.01	0.19	0.16	0.44	0.41	0.44	0.45
