# Supplementary material for: Characterisation of the Cullin-3 mutation that causes a severe form of familial hypertension and hyperkalaemia
Source: EMBO Mol Med. 2015 Aug 18;7(10):1285–306. doi: 10.15252/emmm.201505444 (PMC4604684; doi:10.15252/emmm.201505444)
Supplement: Supplementary file 7 [file emmm0007-1285-sd7.pdf]

6A		MBP	MBP	SBP	SBP	DBP	DBP	HR	HR
		mmHg		mmHg		mmHg		bpm	bpm
	**Group**	**WT**	**Δ403-459**	**WT**	**Δ403-459**	**WT**	**Δ403-459**	**WT**	**Δ403-459**
		83.3	91.8	105.0	115.0	72.5	80.2	410	538
		82.9	90.0	102.7	112.4	73.1	78.8	569	570
		78.8	93.0	96.5	117.8	69.9	80.6	566	541
		75.9	86.9	96.5	112.9	65.5	73.8	536	485
		84.1	103.6	104.4	127.8	73.9	91.5	572	534
		76.8	91.9	97.6	118.4	66.5	78.7	497	440
		82.5	102.6	103.1	126.2	72.1	90.8	531	503
		79.8	85.4	98.9	110.8	70.2	72.7	568	492
		84.7	96.2	107.7	122.3	73.2	83.2	553	473
		80.6	86.0	101.7	111.6	70.1	73.3	437	397
		84.1	89.9	106.8	113.0	72.8	78.4	453	497
		85.3	97.5	104.7	126.6	75.7	83.0	578	417
		77.9	97.4	97.1	126.1	68.2	83.1	561	475
		79.7	92.5	98.5	119.5	70.3	79.0	577	451
		77.6	93.6	95.3	120.2	68.8	80.3	540	425
		81.9	98.5	104.7	123.3	70.5	86.1	395	561
		81.0	93.9	101.1	117.9	70.9	81.9	563	505
		80.5	99.1	99.6	126.9	70.9	85.1	537	463
	**N**	18	18	18	18	18	18	18	18
	**mean**	81.0	93.9	101.2	119.4	70.8	81.1	525	487
	**sem**	0.7	1.3	0.9	1.4	0.6	1.3	15	12

6B		AIx	AIx	AP	AP	PP	PP	$\tau_{bourgeois}$	$\tau_{bourgeois}$
		%		mmHg		mmHg		s^{-1}	
	**Group**	**WT**	**Δ403-459**	**WT**	**Δ403-459**	**WT**	**Δ403-459**	**WT**	**Δ403-459**
		**	45	10.52	15.58	32.5	34.8	0.57	0.60
		35	38	9.79	12.68	29.6	33.6	0.63	0.57
		37	38	10.42	14.27	26.6	37.2	0.54	0.67
		33	41	9.43	15.99	31.0	39.1	0.59	0.64
		31	45	12.25	16.52	30.5	36.3	0.64	0.75
		39	50	12.74	19.97	31.1	39.7	0.56	0.69
		41	58	12.67	20.69	31.0	35.4	0.51	0.70
		44	50	14.18	19.12	28.7	38.2	0.72	0.67
		41	56	16.42	21.88	34.5	39.1	0.63	0.59
		52	57	17.68	21.85	31.6	38.3	0.55	0.62
		52	53	14.21	18.43	34.0	34.7	0.58	0.71
		49	56	10.86	24.31	29.0	43.6	0.65	0.61
		37	55	12.04	23.77	28.9	43.0	0.56	0.64
		42	50	12.00	20.65	28.2	40.9	0.62	0.67
		45	60	20.03	23.81	26.4	39.9		
		58	47	12.07	17.52	34.3	37.2		
		40	52	14.19	18.68	30.2	36.0		
		49	54	***	22.56	28.8	41.8		
	**N**	17	18	17	18	18	18	14	14

**mean**	42.6	50.3	13.0	19.3	30.4	38.3	0.59	0.65
**sem**	1.8	1.6	0.7	0.8	0.6	0.7	0.02	0.02

6C **Phenylephrine dose-response curve (MAP response to each dose increme**

**Group**	**WT**	**WT**	**WT**	**WT**	**WT**
**Dose µg/kg BW**					
0.001	***	***	105	101	108
0.01	122	108	105	101	108
0.1	122	106	105	101	107
0.316	123	109	103	101	108
1	123	110	107	104	110
3.16	129	113	112	110	117
10	139	122	122	124	124
31.6	148	131	138	134	136
100	166	151	157	155	149
316	167	156	158	166	159
400	162	156	***	158	***

**Group**	**Δ403-459**	**Δ403-459**	**Δ403-459**	**Δ403-459**	**Δ403-459**
**Dose µg/kg BW**					
0.001	***	***	123	119	129
0.01	115	122	122	118	128
0.1	118	123	122	117	125
0.316	118	121	122	124	125
1	120	123	123	125	130
3.16	124	129	130	130	140
10	133	137	136	138	146
31.6	144	146	146	152	153
100	152	156	168	163	166
316	165	170	170	175	179
400	161	171	***	178	182

Averages

**Group**	**WT**	**WT**	**WT**	**Δ403-459**	**Δ403-459**
**Dose µg/kg BW**	**N**	**mean**	**sem**	**N**	**mean**
0.001	13	106	1.1	13	124
0.01	15	107	1.4	15	122
0.1	15	107	1.5	15	122
0.316	15	106	1.7	15	122
1	15	108	1.6	15	125
3.16	15	113	2.0	15	131
10	15	122	1.9	15	140
31.6	15	135	1.8	15	153
100	15	151	1.7	15	165
316	15	160	1.5	15	175
400	6	160	1.7	7	174

nt)

WT	WT	WT	WT	WT	WT	WT	WT
105	103	105	114	109	107	108	102
107	105	105	112	108	106	105	101
105	105	107	112	111	105	106	99
105	105	107	113	110	105	106	97
110	110	108	116	111	105	107	97
114	115	109	124	114	109	110	99
122	126	121	135	122	119	119	111
139	139	130	150	135	131	133	126
154	152	146	161	150	147	146	149
158	167	160	172	157	154	152	161
***	159	160	167	***	***	***	***
**Δ403-459**	**Δ403-459**	**Δ403-459**	**Δ403-459**	**Δ403-459**	**Δ403-459**	**Δ403-459**	**Δ403-459**
130	124	118	128	116	121	131	118
129	123	119	125	117	121	126	117
129	125	118	126	116	119	123	117
131	124	119	126	116	120	129	117
133	122	125	127	118	121	135	118
141	133	128	134	124	128	143	123
156	139	139	145	135	140	156	132
167	157	147	160	153	151	171	145
176	165	162	174	170	166	182	163
188	171	174	180	184	171	190	170
184	***	172	***	***	171	***	***

**Δ403-459 sem**
1.5
1.1
1.1
1.1
1.3
1.6
2.0
2.2
2.2
2.0
3.3

WT	WT
101	108
101	108
101	105
98	104
100	106
102	111
111	121
128	131
145	143
159	153
***	***

Δ403-459	Δ403-459
128	127
127	123
125	123
122	121
125	123
132	126
138	135
152	146
163	155
173	170
***	***

Angiotensin-II (AngII) dose-response curve (SBP response

Group	WT	WT	WT	WT
Dose µg/kg BW				
0.0001	121.3	109.7	106.3	95.72
0.001	121.6	109	103.2	92.9
0.01	121.6	109.1	102.5	91.94
0.0316	121.8	110.1	101.5	89.43
0.1	129	117.8	104.7	99.74
0.316	129.7	119.4	113.5	110.1
1	141.7	131.4	122.2	123.3
3.16	142.1	135.2	130.9	132.4
10	147	136.3	129.6	137.9

Dose µg/kg BW	Δ403-459	Δ403-459	Δ403-459	Δ403-459
0.0001	108.6	117.5	119.4	122.8
0.001	114.5	118	118.3	120.5
0.01	111.7	119.9	121.2	119.2
0.0316	118.2	119.5	119.5	121.5
0.1	125.6	125.1	127.8	124.3
0.316	123.9	129.4	135.1	131.4
1	133.6	144.4	143.8	135.1
3.16	138.6	151	148.6	141.9
10	147.1	155.6	150.2	143.4

Averages

Group	WT	WT	WT	Δ403-459
Dose µg/kg BW	N	mean	sem	N
0.0001	15	106	2.2	15
0.001	15	104	2.3	15
0.01	15	103	2.5	15
0.0316	15	103	3.0	15
0.1	15	107	2.9	15
0.316	15	113	3.0	15
1	15	122	2.6	15
3.16	15	130	2.3	15
10	13	135	2.0	14

to each dose increment)

WT	WT	WT	WT	WT	WT	WT	WT	WT
112.4	109.9	104.6	107.9	116.2	112.1	108	94.26	96.9
109.8	109	102.2	106.6	112.2	108.2	103.7	92.94	91.88
110	108.2	100.3	107.3	113.8	104.2	100.6	92.39	87.39
109.2	107.1	99.12	105	123.1	108.6	99.23	93.02	86.71
113.2	112.8	103.1	104.5	122.7	110.8	103.9	97.3	92.03
120.3	120.2	106.9	108.5	137.9	118.2	110.6	105.4	97.67
127.2	127.2	119.3	115.7	139	121.2	117	117.7	108.2
134.6	130.9	134.8	127.1	149.9	131.3	124.4	120.9	118
140.1	136.3	148.4			132.5	129.9	134.6	125.4

Δ403-459	Δ403-459	Δ403-459	Δ403-459	Δ403-459	Δ403-459	Δ403-459	Δ403-459	Δ403-459
128.7	135.1	125.5	123.8	130.5	111.1	123.1	135.4	121
121.3	130.2	125.4	119	128.6	112.4	118.2	132.8	119
120.9	129	122.7	118	127.8	112.4	117.3	131	117.7
121.9	128.9	129.8	118.4	130.2	113.4	116	130.7	116.7
132	137.2	129.1	124.4	134.2	122.3	124.8	134.6	121.3
140	145.5	140.4	130.3	138.6	128.4	129.8	137.4	124
150.2	145.5	142.3	140.2	143.5	140.4	140.4	145	130.9
153.2	153.7	153.6	146.3	148	146.1	147.1	149.8	137.1
165.1	159.1		152.9	151.3	150.8	151.2	151.5	142.4

Δ403-459 mean	Δ403-459 sem
123	2.1
121	1.6
121	1.5
121	1.6
129	1.4
134	1.7
141	1.4
147	1.3
152	1.6

WT	WT
93.75	101.9
91.52	101.5
91.2	100.4
87.39	98.06
92.12	100.5
98.06	104.7
108.9	113.3
118.9	124.2
129.4	133.1

Δ403-459	Δ403-459
115.5	121.3
115	120.8
121	128.7
113.2	119.8
129.5	137
130.9	137.8
140.4	145.6
146.4	148.3
152.6	154.4
