# Supplementary material for: Characterisation of the Cullin-3 mutation that causes a severe form of familial hypertension and hyperkalaemia
Source: EMBO Mol Med. 2015 Aug 18;7(10):1285–306. doi: 10.15252/emmm.201505444 (PMC4604684; doi:10.15252/emmm.201505444)
Supplement: Supplementary file 8 [file emmm0007-1285-sd8.pdf]

7				
	**Avg - Media Thickness** µm	**Avg - Media Thickness** µm	**Avg - Elastin number**	**Avg - Elastin number**
**Group**	**WT**	**Δ403-459**	**WT**	**Δ403-459**
	93.36	120.54	5.07	5.30
	97.39	137.77	5.08	5.85
	107.14	106.71	5.33	5.48
	107.32	125.26	5.11	5.43
	126.17	132.46	5.68	5.13
	96.92	123.34	5.30	5.26
	91.44	117.02	5.21	5.42
	88.40	133.04	5.54	5.73
	95.16	119.90	4.94	5.35
	93.01	107.21	5.38	5.21
		105.87		5.38
**N**	10	11	10	11
**mean**	99.6	122.3	5.3	5.4
**sem**	3.7	3.5	0.1	0.1
