# Supplementary material for: Characterisation of the Cullin-3 mutation that causes a severe form of familial hypertension and hyperkalaemia
Source: EMBO Mol Med. 2015 Aug 18;7(10):1285–306. doi: 10.15252/emmm.201505444 (PMC4604684; doi:10.15252/emmm.201505444)
Supplement: Supplementary file 9 [file emmm0007-1285-sd9.zip › Source Data for Expanded View and Appendix/EMM_5444_SourceDataAppendixFigS6.pdf]

Appdx_6

		Mean pMYPT1 blot density		
	**Group**	**WT**	**Δ403-450**	**Ratio Δ403-450/WT**
	**Blot 1**	1156597	608056	1.90
	**Blot 2**	1374592	891127	1.54
	**Blot 3**	83408	46284	1.80
