# Supplementary material for: Characterisation of the Cullin-3 mutation that causes a severe form of familial hypertension and hyperkalaemia
Source: EMBO Mol Med. 2015 Aug 18;7(10):1285–306. doi: 10.15252/emmm.201505444 (PMC4604684; doi:10.15252/emmm.201505444)
Supplement: Supplementary file 9 [file emmm0007-1285-sd9.zip › Source Data for Expanded View and Appendix/EMM_5444_SourceDataFigEV3.pdf]

Fig EV3

A PLASMA

Analyte	Cr	Ca	K	Mg	Na	P
Na_diet	NNa	NNa	NNa	NNa	NNa	NNa
Group	WT	WT	WT	WT	WT	WT
	16.70	2.09	4.75	0.88	133	5.71
	14.21	2.13	4.70	0.77	126	4.88
	10.95	1.98	4.68	0.78	133	3.85
	10.22	1.82	4.00	0.81	123	4.98
	21.25	2.04	5.05	0.83	131	3.95
	11.64	2.06	4.27	0.75	133	4.08
	16.35	2.19	5.47	0.87	135	6.30
	9.24	2.35	5.49	0.87	139	7.18
	10.53	2.12	4.87	0.92	131	5.80
	17.57	2.07	5.75	0.92	132	4.66
	19.70	2.09	5.19	0.90	129	5.11
	18.07	2.17	4.87	0.94	126	5.23
	15.45	2.10	5.66	0.99	135	5.84
	17.65	2.05	5.19	0.85	129	5.46
	11.24	2.14	6.00	0.88	131	5.32
	20.53	2.00	5.54	0.84	125	5.44
N	16	16	16	16	16	16
mean	15.08	2.09	5.09	0.86	131	5.24
sem	1.03	0.03	0.14	0.02	1.08	0.22

B URINE

Analyte	Cr	Ca	K	Mg	Na	P
Na_diet	NNa	NNa	NNa	NNa	NNa	NNa
Group	WT	WT	WT	WT	WT	WT
	4.40	0.50	111	26.1	94.7	33.8
	4.56	0.69	157	27.1	79.1	59.5
	6.07	2.06	174	44.7	95.8	66.1
	6.81	0.97	111	43.6	101.9	14.7
	4.32	2.21	206	30.8	180.2	109.4
	3.67	1.86	126	34.4	110.6	30.1
	2.11	0.24	122	16.4	88.7	54.7
	2.06	0.50	115	17.3	78.7	33.5
	3.45	0.87	124	25.2	226.8	50.4
	5.69	1.92	229	67.3	87.2	128.0
	5.90	0.84	108	43.4	113.4	51.8
	6.12	1.27	102	50.8	212.6	82.8
	4.06	1.00	317	42.2	142.1	136.0
	4.50	1.04	457	52.7	84.3	137.5

		5.22	1.26	280	40.1	59.8	86.6
		4.64	1.67	351	55.2	89.9	187.6
		2.89	0.89	115	18.0	86.0	21.2
		5.19	1.26	87	35.9	100.4	21.2
	**N**	18	18	18	18	18	18
	**mean**	4.5	1.2	182.9	37.3	112.9	72.5
	**sem**	0.3	0.1	25.4	3.5	11.4	11.9

**C**	**PLASMA**						
	**Analyte**	**Urea mmol/L**	**Glu mmol/L**	**Hct %**	**TCO2**	**Anion Gap**	**Hb g/L**
	**Na_diet**	**NNa**	**NNa**	**NNa**	**NNa**	**NNa**	**NNa**
	**Group**	**WT**	**WT**	**WT**	**WT**	**WT**	**WT**
		6.4	11.2	45	25	10	153
		5.3	**	46	28	13	156
		6.6	21.5	44	23	11	150
		7.1	20.7	44	22	11	150
		5.9	17.4	46	25	26	156
		8.3	21.7	39	26	13	133
		11.1	18.6	40	24	13	136
		8.5	10.1	41	22	14	139
		5.8	19.1	42	22	9	143
		8.6	9.1	44	23	12	150
		7.4	17.3	44	20	15	150
		5.4	9.6	46	22	12	156
		7.7	15.4	43	22	13	146
		7.5	15.5	38	23	14	129
	**N**	14	13	14	14	14	14
	**mean**	7.3	15.9	43.0	23.4	13.3	146.2
	**sem**	0.4	1.3	0.7	0.6	1.1	2.5

Cr LNa WT	Ca LNa WT	K LNa WT	Mg LNa WT	Na LNa WT	P LNa WT	Cr NNa Δ403-459
15.24	2.22	4.44	0.81	137	5.80	12.03
22.10	2.13	5.48	0.91	137	6.55	10.42
18.04	2.20	5.46	0.99	139	6.79	14.16
18.93	2.41	4.75	1.00	144	7.64	14.64
15.76	2.30	4.52	0.84	138	4.12	10.04
22.92	2.06	3.77	0.83	125	4.58	11.34
11.34	2.47	5.24	0.92	147	6.74	16.94
17.09	2.36	4.67	0.95	140	6.78	10.52
15.96	2.24	4.13	0.75	134	5.49	13.21
22.21	2.33	4.64	0.89	131	5.58	12.46
21.70	2.12	4.24	0.87	132	5.38	13.91
26.27	2.16	6.07	0.94	133	6.28	19.97
18.10	2.42	5.41	0.93	133	5.78	19.00
14.71	2.29	4.86	0.85	132	5.48	17.68
16.35	2.16	5.21	0.89	136	5.40	12.98
18.46	2.14	6.06	0.91	131	5.30	20.92
22.79	2.09	5.29	0.94	131	5.92	17.97
						23.52
						25.55
						17.44
						20.17
						20.92
						16.14
17	17	17	17	17	17	21
18.70	2.24	4.95	0.89	135	5.86	15.95
0.95	0.03	0.16	0.02	1.34	0.22	1.00

Cr LNa WT	Ca LNa WT	K LNa WT	Mg LNa WT	Na LNa WT	P LNa WT	Cr NNa Δ403-459
5.36	0.27	39	6.6	1.7	8.7	4.03
2.96	0.05	39	4.1	2.9	21.2	4.61
2.47	0.35	43	7.1	0.0	2.5	4.27
2.31	0.21	23	4.6	0.0	21.5	3.88
3.68	0.17	43	4.2	1.1	6.7	4.51
6.15	0.25	44	9.3	0.4	21.9	4.64
4.48	0.29	67	8.8	0.3	28.0	3.77
4.98	1.42	61	7.4	2.9	15.8	3.52
5.50	0.51	24	7.8	0.0	1.9	5.92
5.21	0.39	15	8.9	0.9	2.9	3.01
1.92	0.04	45	6.9	0.0	4.8	4.30
2.10	0.44	51	7.3	0.0	1.4	3.22
1.52	1.30	20	4.9	0.0	1.0	5.88
5.34	0.82	49	7.2	0.3	1.2	2.68

4.70	0.28	23	7.9	0.2	4.3		1.79
2.58	0.08	27	3.8	0.0	0.8		4.26
							3.63
							3.10
							0.82
							1.92
							3.18
16	16	16	16	16	16		21
3.8	0.4	38.4	6.7	0.7	9.0		3.7
0.4	0.1	3.9	0.5	0.3	2.4		0.3

**Urea mmol/L NNa Δ403-459**	**Glu mmol/L NNa Δ403-459**	**Hct % NNa Δ403-459**	**TCO2 NNa Δ403-459**	**Anion Gap NNa Δ403-459**	**Hb g/L NNa Δ403-459**
8.2	20.3	43	19	12	146
5.6	19.3	46	18	12	156
7.9	13.1	46	22	9	156
5.8	14.6	47	20	11	160
7	21.2	41	22	11	139
8.5	14	44	20	11	150
6.1	16.8	41	21	11	139
6.2	9.3	43	19	13	146
6.8	17	43	20	12	146
8.9	15	43	18	14	146
6.7	15.5	44	22	9	150
8.4	17.1	42	19	10	143
7.3	7.8	38	18	12	129
13	13	13	13	13	13
7.2	15.5	43.2	19.8	11.3	146.6
0.3	1.1	0.7	0.4	0.4	2.4

Ca NNa Δ403-459	K NNa Δ403-459	Mg NNa Δ403-459	Na NNa Δ403-459	P NNa Δ403-459	Cr LNa Δ403-459	Ca LNa Δ403-459	K LNa Δ403-459
2.22	5.84	0.88	136	5.99	9.81	2.22	5.29
2.08	5.47	0.86	128	5.37	10.98	2.22	6.00
2.25	6.12	0.88	133	6.15	11.59	2.17	4.89
1.95	5.27	0.83	122	5.32	11.73	2.35	5.41
2.39	6.72	0.89	135	5.97	17.67	2.23	5.53
2.16	5.81	0.92	133	6.20	13.34	2.21	5.45
2.36	6.21	1.05	142	6.81	16.08	2.28	4.92
2.39	6.09	1.00	137	6.79	16.04	2.25	5.78
2.21	6.15	0.89	126	6.61	17.86	2.29	4.86
2.35	5.98	0.97	136	5.94	14.28	2.18	4.89
2.33	6.00	0.95	131	6.05	18.49	2.26	5.26
2.16	6.72	0.95	138	5.50	14.60	2.12	5.88
2.07	5.56	0.87	133	5.14	18.10	2.03	4.63
2.13	6.39	0.86	127	4.83	14.32	2.36	5.14
2.13	5.85	0.88	129	5.11	14.64	2.08	4.93
2.22	6.15	0.96	137	6.20	20.15	2.30	6.54
2.23	6.03	0.98	131	5.86	16.25	2.34	5.50
2.31	6.52	1.01	135	6.41	16.26	2.06	6.47
2.24	6.91	1.00	132	6.05	19.02	2.17	5.49
2.06	7.04	0.88	127	5.93	23.72	2.45	6.50
2.32	6.63	0.94	129	5.83	20.87	2.30	6.03
2.34	5.90	0.91	126	5.85	22.97	2.28	5.27
2.10	5.28	0.78	127	5.65	17.34	2.19	5.59
21	21	21	21	21	23	23	23
2.22	6.16	0.93	132	5.91	16.35	2.23	5.49
0.03	0.10	0.01	1.07	0.12	0.78	0.02	0.12

Ca NNa Δ403-459	K NNa Δ403-459	Mg NNa Δ403-459	Na NNa Δ403-459	P NNa Δ403-459	Cr LNa Δ403-459	Ca LNa Δ403-459	K LNa Δ403-459
0.67	133	20	66	23.6	3.33	0.07	41
1.23	186	39	113	95.0	1.62	0.98	63
0.20	86	9	32	69.4	3.40	0.63	51
0.30	188	12	87	111.2	3.57	0.13	64
1.27	267	42	197	93.1	2.77	0.77	43
0.85	113	31	72	52.4	4.36	0.77	29
1.75	287	41	152	102.6	4.09	0.33	42
0.51	113	26	100	57.6	4.75	0.25	51
0.53	244	29	118	181.6	4.61	0.10	47
0.82	100	26	203	22.7	5.13	0.82	47
0.65	103	32	103	105.0	0.98	0.10	75
0.41	93	19	37	6.1	1.82	0.00	16
0.98	120	43	74	11.2	5.07	0.10	24
0.81	91	24	14	6.4	1.44	1.20	70

0.08	58	11	28	15.8	5.03	0.14	27
1.78	101	40	172	45.7	1.51	0.00	40
0.48	153	20	68	54.9	4.96	0.12	19
0.68	94	27	57	5.7	3.47	0.09	26
0.06	32	7	57	5.9	2.50	0.11	37
0.17	69	13	10	3.3			
0.82	224	39	128	109.0			
21	21	21	21	21	19	19	19
0.7	136.1	26.3	89.9	56.1	3.4	0.4	42.7
0.1	15.8	2.6	12.6	10.9	0.3	0.1	4.0

Mg<br>LNa<br>Δ403-459	Na<br>LNa<br>Δ403-459	P<br>LNa<br>Δ403-459
0.84	137	5.93
1.03	141	7.06
0.89	136	6.65
0.95	143	7.15
0.84	137	6.14
1.02	134	6.63
0.81	136	6.39
0.85	140	6.11
0.75	143	6.51
0.73	138	5.67
0.80	129	4.84
0.82	133	5.53
0.72	129	4.46
0.78	140	5.61
0.84	133	6.42
0.87	133	6.55
0.84	134	6.08
1.02	136	4.79
0.82	126	4.64
1.04	137	5.45
0.99	143	5.37
0.90	133	5.65
0.88	130	4.73
23	23	23
0.87	136	5.84
0.02	1.02	0.17

Mg<br>LNa<br>Δ403-459	Na<br>LNa<br>Δ403-459	P<br>LNa<br>Δ403-459
4.6	0.1	10.6
7.7	1.3	14.6
10.2	0.5	9.4
9.4	0.0	10.9
7.4	1.4	6.8
6.5	0.0	1.8
5.6	0.0	9.4
6.9	0.0	10.0
4.5	1.8	11.4
9.8	0.3	5.7
9.8	0.0	1.7
3.0	0.7	2.7
5.8	2.1	4.9
9.0	0.0	3.1

7.2	3.4	6.2
7.9	0.0	1.4
5.9	2.6	3.7
7.7	0.6	1.0
6.8	0.0	0.7

19	19	19
7.1	0.8	6.1
0.5	0.2	1.0
